# A De Novo Luciferase Bioconjugate for the Cas13-Based Detection of Influenza A

**DOI:** 10.1021/jacsau.5c00576

**Published:** 2025-07-28

**Authors:** Mary Canzano, Joseph L. Harman, Yanira Méndez, Alfredo Quijano-Rubio, Daniel-Adriano Silva, Gonçalo J. L. Bernardes

**Affiliations:** † Yusuf Hamied Department of Chemistry, 2152 University of Cambridge, Cambridge CB2 1EW, U.K.; ‡ Monod Bio Inc., Seattle, Washington 98109, United States; § Translational Chemical Biology Group, Spanish National Cancer Research Centre (CNIO), Madrid 28029, Spain

**Keywords:** Cas13, diagnostics, de novo proteins, luciferase, bioconjugation, optical biosensor

## Abstract

Rapid, accurate, and accessible diagnostics for pathogenic infections are of vital importance for the prevention of disease transmission and mitigation of future pandemics. Biosensors employing the CRISPR nuclease Cas13 have enabled robust detection of viral RNA. However, existing Cas13-based diagnostics primarily utilize fluorescent or lateral flow assay (LFA) readouts, impeding detection in complex sample media. Here, we have developed a luciferase-based Cas13 diagnostic containing a protein-nucleic acid conjugate, coined Selective One-pot LUminescent Nucleic Acid Reporter (SOLUNAR). SOLUNAR utilizes RNA which has been site-selectively bioconjugated to block the binding of a de novo heterodimeric protein pair. In the presence of a viral target, the sensor RNA is cleaved by the collateral activity of Cas13, restoring binding and yielding a luminescent signal by the reconstitution of a tagged de novo split luciferase. An integral component of SOLUNAR, the use of de novo proteins enabled precise engineering of site-selective chemical handles and tunable binding affinities to modulate activity. SOLUNAR selectively detects Influenza A H1N1 at room temperature in one-pot with a limit of detection (LOD) of 1.5 nM without amplification and is compatible with samples containing ≤10% serum. Our findings demonstrate the utility of luminescence in Cas13 diagnostics and reveal the potential applications of this platform to activity-based enzymatic sensing.

## Graphical abstract

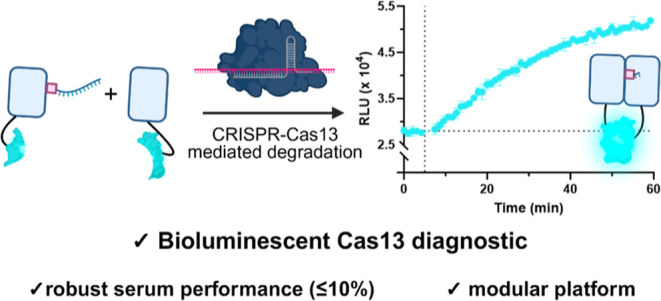

Zoonotic viruses pose a serious threat to human health, particularly for vulnerable populations such as the elderly and immunocompromised.^1^ Prevention of disease transmission has been greatly aided by the development of rapid, sensitive, specific, and accessible diagnostic tools. Recently, CRISPR-Cas13 has been employed to develop molecular diagnostics, allowing for nucleic acid–based detection of viral infections under isothermal conditions.^2^ Various Cas13-based systems against several viruses including Sars-CoV-2,^3–13^ Ebola,^5,9,14^ and Zika^2,5,9,15,16^ viruses have been developed which exhibit exquisite sensitivity (∼2 aM target RNA or DNA),^2,5,6,8–10,15–17^ have been tested in the clinic,^7^ and demonstrate great potential in point-of-care applications.^2,4–6,10,12,15,18–20^ Despite these advances, there remain limitations; notably, most of the systems rely on fluorescent or LFA readouts which require preisolation of nucleic acids from clinical samples and risk sample contamination, respectively. Bioluminescence poses an advantageous alternative and has yet been unexplored for this application.

CRISPR-Cas13 is a class 2 type VI CRISPR system which enables the degradation of single-stranded RNA (ssRNA) by two Higher Eukaryotes and Prokaryotes Nucleotide-binding (HEPN) domains.^21,22^ Cas13 demonstrates both cis and trans cleavage of RNA: cis cleavage refers to the sequence-specific cleavage of ssRNA using designed CRISPR guide RNAs (crRNAs) while trans cleavage, also known as collateral activity, is initiated only upon formation of the Cas13-crRNA-target complex, and involves the nonspecific cleavage of all ssRNA, regardless of sequence.^21–23^ As a robust enzyme, Cas13 is functional at mild temperatures (20–37 °C),^10,24^ benefits from rapid collateral cleavage (*k*_cat_ ≥ 1 s^–1^),^25^ and, when paired with appropriately designed crRNAs, can target any ssRNA of interest.

Harnessing the power of collateral activity, Specific High sensitivity Enzymatic Reporter unLOCKing (SHERLOCK) emerged as the first Cas13-based diagnostic.^2^ The principle of SHERLOCK, and subsequent systems, involves three major steps: (1) recombinase polymerase amplification (RPA) or reverse transcription-RPA (RT-RPA) to amplify the viral target of interest, (2) T7-transcription to transcribe ssRNA from the amplified DNA, and (3) treatment with Cas13 in the presence of a fluorescence-quenched reporter. Commonly, the fluorescence reporter is a 6mer ssRNA with 5′ fluorescein (FAM) and 3′ quencher modifications. Upon cleavage of the RNA by collateral activity, a fluorescent signal is produced from the separation of the fluorophore and quencher, indicating the presence of target ssRNA in the sample (Figure 1a).

**Figure 1:**
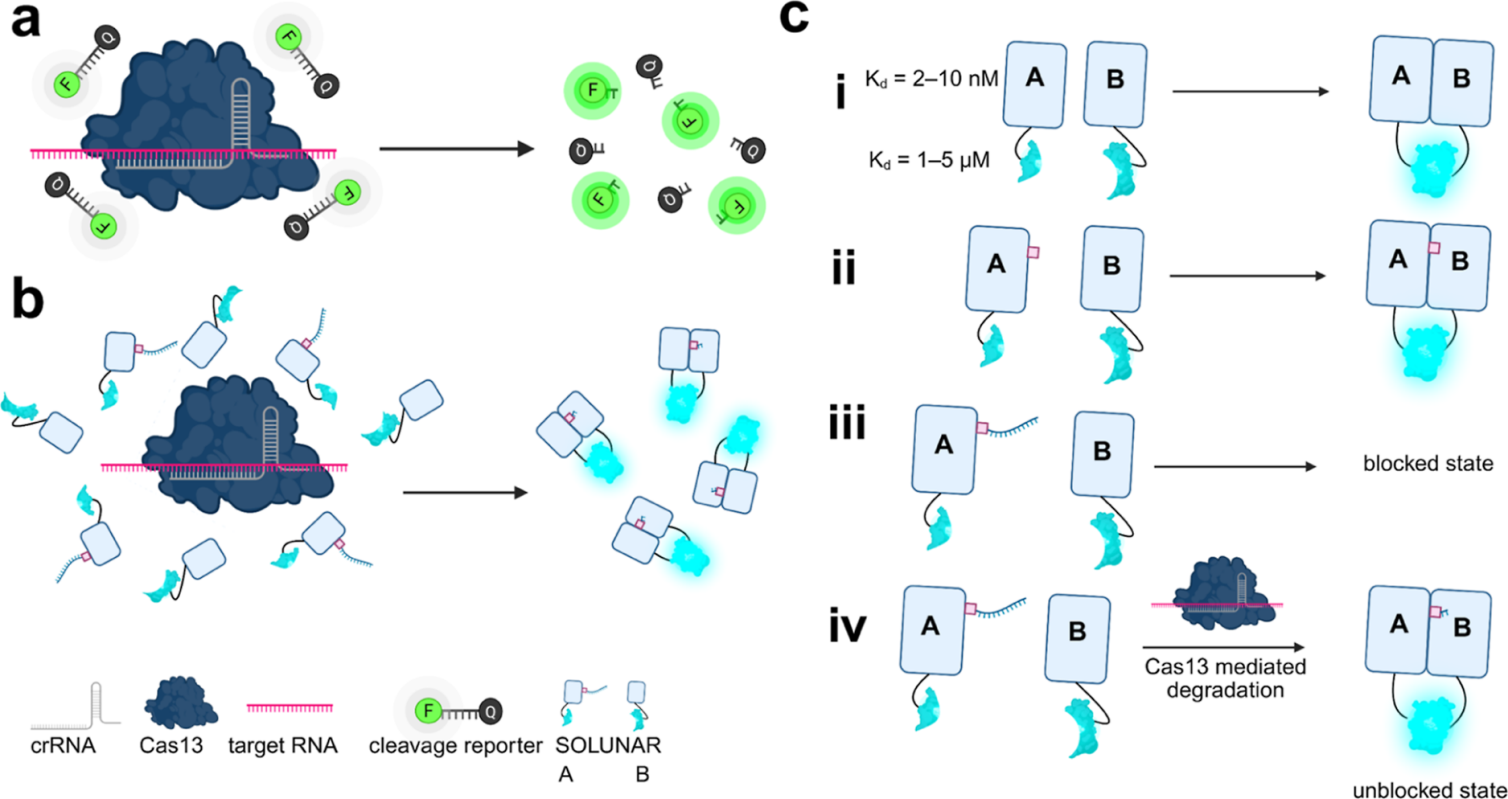
Schematic overview of sensor systems. (a) Previous Cas13 sensors.^2^ (b) This work: proposed sensor system. (c) Proposed function of SOLUNAR. Model interaction of wild type proteins LHD101A and LHD101B tagged to LuxSit s-Pro (i), linker-modified A protein and unmodified B protein (ii), protein A-linker-RNA conjugate with B (SOLUNAR) without Cas13 present (iii), and SOLUNAR with Cas13 present (iv). Created in BioRender. Bernardes, G. (2025) https://BioRender.com/h55s961.

Primarily, advances in Cas13-based diagnostics have focused on developing amplification-free systems, eliminating the need for both steps 1 and 2 of SHERLOCK while maintaining comparable sensitivity.^3–5,11–14,17,20,24,26–30^ However, limited efforts have been made to explore alternative reporting strategies with the exception of a few materials-based approaches.^4,26,31^ While fluorescence can produce robust, wavelength-specific signals, its performance can suffer in complex clinical samples due to features such as autofluorescence, light scatter, and photobleaching.^32^ Therefore, most Cas13-based diagnostics require preisolation and purification of nucleic acids from clinical samples (i.e., saliva, serum, urine, etc.) before amplification, a step which takes time, reagents, and appropriate handling of samples to avoid cross-contamination. Direct interpretation of clinical samples while maintaining high sensitivity remains a challenge, leading us to investigate alternative reporting strategies.

Bioluminescence refers to the production of photons from the enzymatic oxidation of a substrate. Compared to other optical techniques, luminescence is preferable for the analysis of biological samples—notably serum which is highly autofluorescent—due to the production of light in the absence of an external light excitation source, reducing light scatter and eliminating photobleaching. Further, due to the low intrinsic bioluminescence of clinical samples, high sensitivity is achieved with minimal background noise.^33,34^ Luciferase technology has been used for a variety of biosensing applications, namely through the excitation of a proximal fluorophore via bioluminescence resonance energy transfer (BRET) or through the reconstitution of a split luciferase such as NanoLuc.^35,36^ Various split luciferase diagnostic sensors have been developed for the detection of a range of targets including antibodies,^37,38^ viral proteins,^39,40^ and biomarkers^41^ in clinical samples/sample mimics. However, to date, bioluminescence has yet to be applied to Cas13 diagnostics.

Recently, van der Veer and co-workers developed a bioluminescent nucleic acid sensor—termed LUNAS^42^—which uses a dCas9-split NanoLuc fusion to detect RNA. With comparable sensitivities to fluorescence-based Cas13 diagnostics (∼200 copies/μL),^11,12^ LUNAS enables direct interpretation of clinical samples including serum without nucleic acid purification. However, Cas9 sensors can be complex to design due to protospacer adjacent motif (PAM) sequence requirements, and LUNAS does not invoke any catalytic activity, meaning that each viral molecule can only cause the reconstitution of one luciferase.^43,44^ Additionally, LUNAS requires the design of two crRNAs which must anneal to the target at spatially proximal sites, a feature which can be cumbersome and can prevent design for targets with complex secondary structures (i.e., pseudoknots, G-quadruplexes, etc.). Thus, we envisaged that we could apply a similar luciferase technology—given its superior performance in clinical samples—to the development of a Cas13 sensor, an enzyme with no strict sequence requirements and rapid catalytic turnover.^23^

To design a biosensor capable of luminescence upon Cas13 mediated RNA cleavage, we selected a de novo heterodimer protein pair, LHD101A and LHD101B, which spontaneously binds in solution. Previously designed in a library of proteins to selectively heterodimerize over homodimerization or further multimerization, these constructs are small, highly stable, bind with nanomolar affinity, and have performed well as fusions.^45^ The LHD101 pair has been utilized to develop cross-linked hydrogels and study cellular processes,^46,47^ but has yet to be explored for diagnostic biosensing applications. We rationalized that we could modulate the binding interaction between LHD101A and LHD101B by site-selectively attaching RNA to one monomer (A) via a covalent linker to block binding, allowing for a turn-on sensor which exists either in a ‘blocked’ state with intact RNA or an ‘unblocked’ state upon RNA degradation (Figure 1c).

To enable detection of this interaction, we genetically fused components of a de novo split luciferase, split LuxSit Pro (LuxSit s-Pro),^48^ to each monomer. As an optimized derivative of the first de novo luciferase, LuxSit,^49^ LuxSit s-Pro contains two proteins, αLux and βit, which are small, highly stable, and contain no cysteine residues, making them ideal candidates for both genetic manipulation and post-translational bioconjugation. In this work, we selected the βLux_2 variant of βit, which has a reported affinity of 1−5 μM for αLux.^48^ As novel fusion constructs (LHD101A-βit and LHD101B-αLux), we suspected that the low affinity of αLux and βit (∼1–5 μM) compared to the high affinity of LHD101A and LHD101B (∼2–10 nM) would promote significantly greater luciferase reconstitution upon heterodimer binding. In principle, upon Cas13-mediated cleavage of the sensor RNA, heterodimer binding can be restored, yielding a marked increase in luminescence.

Here, we report the development of a de novo protein-RNA conjugate biosensor as a reporter for Cas13-based diagnostics, coined SOLUNAR (Figure 1b). SOLUNAR is capable of detecting Influenza A ssRNA with a limit of detection (LOD) of 1.5 nM in 1 h and is uniquely compatible with up to 10% serum. As a one-pot method with no preamplification technique, the entire system is functional at room temperature and requires only a photon detector for analysis. As the first published biosensor system utilizing split LuxSit Pro, we demonstrate the power of this de novo luciferase and post-translational bioconjugation for the biosensing of viral RNA and point toward future applications in activity-based enzymatic biosensing.

## Results and Discussion

### Design of De Novo Heterodimers with Single Cysteines as Chemical Handles

To develop a multicomponent sensor system, the use of de novo proteins enabled flexible and precise design. De novo proteins benefit from high levels of stability and thus can withstand modification with minimal effects on structure, facilitating engineering of dynamic systems with precise functions.^50,51^ LHD101A and LHD101B were selected as a scaffold for the sensor, in which LHD101A would be site-selectively modified with RNA.^45^ To incorporate a site-selective handle, we introduced a single cysteine at the binding interface. Cysteine, containing a highly nucleophilic thiolate group, is uniquely reactive among canonical amino acids and is thus a common target for bioconjugation strategies.^52^ Further, the amino acid has a low natural abundance (<2% of residues)^53^ and was not found in the wild-type heterodimer sequences, allowing for facile introduction of a single cysteine.

Mutations of interface residues by substitution with cysteines were computationally evaluated. Using AF2-Multimer,^54^ we predicted the structures of the heterodimers incorporating each mutation, applying in silico metrics such as a predicted aligned error (PAE) < 5 and a per-residue local-distance difference test (pLDDT) > 90. These metrics have been shown to partially discriminate binders from nonbinders.^55^ Four single-cysteine substitutions on LHD101A (E60C, I61C, E62C, G65C) were selected for in vitro screening. The wild-type and mutated proteins were genetically fused to the LuxSit s-Pro proteins via a short, flexible linker, and a C-terminal His-Tag was attached. The proteins, LHD101A-βit and LHD101B-αLux (referred to as proteins A and B, respectively, throughout the remainder of the text), were expressed in *E. coli*, purified, and tested to confirm they retained binding affinity by measuring their ability to promote luciferase activity (Figure S60).

### Preparation of Bioconjugates

With the cysteine mutants in hand, heterobifunctional linkers **L1** and **L2** were synthesized for the generation of protein-RNA conjugates. Both linkers contain a maleimide for site-specific Michael addition to the cysteines on protein A and a DBCO functionality for strain-promoted azide–alkyne cycloaddition (SPAAC) with an azide on conjugate RNA, **conjRNA1** (Figure 2a). **L1**, a shorter, more rigid linker, and **L2**, a longer, more flexible linker, were generated to investigate the impact of length and flexibility on sensor performance.

**Figure 2:**
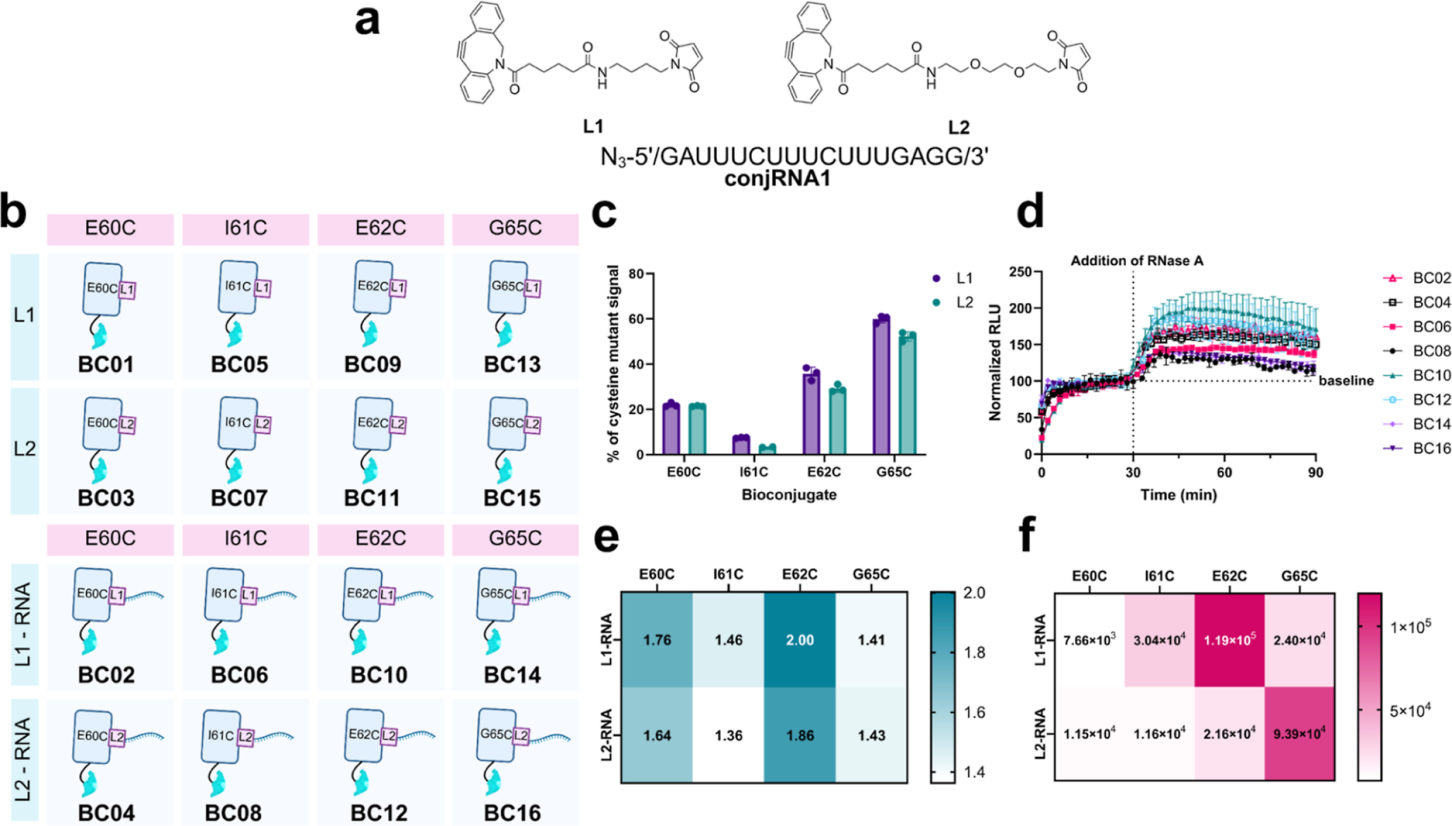
Protein-RNA bioconjugate library. (a) Synthesized bifunctional linkers for protein-RNA bioconjugation and commercial N_3_-functionalized RNA. (b) Library of bioconjugates produced. (c) Relative luminescence of protein-linker conjugates. *N* = 3 for all sets except BC07 (I61C-L2) where *n* = 2. (d) Time-course luminescence assay of each protein-RNA conjugate paired with B protein treated with RNase A after 30 min. Data sets normalized to baseline signal, defined as average signal from time = 26–30 min. *N* = 3 for all sets except BC08 where *n* = 2. (e,f) Performance of protein-RNA bioconjugates according to S/B changes (e) and maximum signals (f) following addition of RNase A. Created in BioRender. Bernardes, G. (2025) https://BioRender.com/n40z061.

Conjugating large biomolecules together can be kinetically challenging and often requires a large excess of one reagent, limiting feasibility relative to protein-small molecule conjugation.^56^ Thus, selection of the thiol-maleimide Michael addition was a logical choice to attach the linker to the protein, given the impressively rapid kinetics (*k*_2_ = 734 M^–1^ s^–1^) and simplicity of the reaction (short reaction times, minimal equivalents needed, etc.).^52,57^ Bioconjugation with native RNA is more challenging given the absence of robust reactive handles. Thus, the selection of the SPAAC reaction was partially driven by the commercial availability of azide-tagged RNA. The SPAAC reaction also benefits from reasonably quick kinetics (*k*_2_ = 0.9 M^–1^ s^–1^) and high product stability, and, unlike copper-catalyzed azide–alkyne cycloaddition (CuAAC), does not require any additional reagents (i.e., sodium ascorbate, Cu­(II), and THPTA), simplifying conjugations.^57^ Additionally, because of SPAAC and Michael addition reaction orthogonality, bioconjugations could be performed in any order and in one-pot.

**ConjRNA1** was designed as a 17mer sequence with high uracil (U) content and no discernible secondary structure (Figure 2a). Although lengthier RNA could theoretically produce a more substantial blocking effect, the length of the oligo was limited to 17 nt to keep costs reasonable given the high cost of azide-modified RNA. The sequence of **conjRNA1** was designed with several U rich regions to enhance the collateral cleavage given the enzyme’s preferential cleavage between sequential Us.^58^ We refrained from incorporating secondary structures such as hairpins or bulge loops to avoid introducing an additional variable which might complicate interpretation.

With these components, a library of 16 bioconjugates was produced, half of which were protein-linker-RNA conjugates (4 proteins × 2 linkers × 1 RNA) and half of which were protein-linker conjugates (4 proteins × 2 linkers) as controls (Figure 2b). Protein-linker conjugation proceeded to completion in 2 h as confirmed by LC–MS, and conjugates were purified by size exclusion chromatography (SEC) before incubation with **conjRNA1** overnight. Conversion to the final product was confirmed by LC−MS prior to a final round of SEC purification, upon which SDS-PAGE was performed to assess sample purity. Importantly, the SPAAC reaction successfully proceeded at room temperature, protecting the sensor RNA which is hydrolytically unstable at higher temperatures.^59^

Given the high isoelectric point (pI) of the A proteins (Table S2), conjugation to negatively charged RNA proved favorable, and all conjugates were successfully generated. However, due to the complex nature of these chimeric biomolecules, some were challenging to characterize. G65C RNA conjugates suffered from poor ionization and were visualized as two adjacent bands by SDS-PAGE (Figures S25, S27, and S48). E60C RNA conjugates displayed the same band pattern by SDS-PAGE yet ionized well and were clearly visualized as single products by LC–MS (Figures S13, S15, and S48). Thus, we suspect that the gel band doubling behavior was due to real-time, partial refolding of the protein during electrophoresis due to the high stability of the protein tertiary structure.

### Luminescence Screening of Protein-RNA Conjugate Library

The protein-linker bioconjugates were first screened in luminescence assays to assess the effect of covalent modification at each residue (Figure 2c). The signal of the protein-linker conjugates can also be imagined as a theoretical maximum for the performance of the corresponding protein-linker-RNA conjugate, given that the linker remains on the protein following RNA cleavage. All conjugates expectedly demonstrated a decrease in signal relative to the naked cysteine mutants. Notably, I61C conjugates suffered from a dramatic reduction in signal, hence indicating that this site may be less amenable to modification. Fortunately, the other conjugates retained at least 20% of their respective mutant’s signal. Generally, **L1** conjugates benefitted from slightly higher signals than corresponding **L2** conjugates, suggesting an overall preference for smaller modifications.

The protein-linker-RNA conjugates were then screened with RNase A, a general RNA cleavage agent. RNase A is extremely robust and does not require additional components for activity,^60^ simplifying testing. In time-course luminescence assays, proteins A and B were allowed to preincubate until a baseline signal was achieved, upon which RNase A was manually added. All conjugates exhibited an increase in signal upon RNA cleavage (Figure 2d).

To assess the efficacy of each conjugate, the signal-to-baseline (S/B) change was determined, defined as the maximum signal following RNA cleavage divided by the baseline signal before the addition of RNase A. Here, all conjugates achieved an S/B change above 1.3, with conjugate E62C-L1-RNA (**BC10**) exhibiting the greatest change at 2.0 (Figure 2e). Comparing conjugates produced from the same cysteine mutants, similar S/B changes were observed, with **L1** conjugates generally slightly outperforming their **L2** counterparts.

The maximum signal of each conjugate was also used to assess conjugate performance (Figure 2f). **BC10** produced the largest signal (∼100,000 RLU), thus making it the best overall hit from the screen. While the other conjugate from the same cysteine mutant, E62C-L2-RNA (**BC12**), exhibited the second highest S/B change at 1.89, its maximum overall signal was significantly lower than that of **BC10**, indicating that while the linker appears to have modest effects on the S/B change, it can have more dramatic effects on the overall signal. Further, preferences for short attachments versus flexible attachments varied with different sites of attachment, as **L1** was found to be preferable for E62C conjugates whereas **L2** was more favorable for G65C conjugates (Figures 2f and S74).

Considering the efficacy of **BC10**, an alternative model using E62C-L1-RNA with a brighter split luciferase (*K*_d_ ∼ 100–500 nM) was also tested (**BC18** and BH-BWT). While a larger signal was achieved, the S/B change was not superior to **BC10**, indicating that the affinities of the sensor components tune the sensor’s performance (Figure S80). Given the importance of having a sizable S/B change, we thus decided to proceed with **BC10**.

### CRISPR-Cas13 System Targeting H1N1[2009]

Considering the success of **BC10** in RNase screens, a CRISPR-Cas13 system was then developed against Influenza A H1N1[2009] using Cas13a from *Leptotrichia wadei* (lwCas13a).^2^ H1N1[2009] was a notably infectious strain of the flu which became pandemic in 2009 due to significant antigenic variation of the hemagglutinin (HA) protein.^61,62^ As a result of widespread transmission, the pandemic strain continues to circulate annually.^63^ Given the range of viruses which can cause similar, nonspecific, flu-like symptoms, diagnostics which can distinguish between these viruses, including different strains of the flu, are vitally important.

To design a CRISPR-Cas13 system against H1N1, we targeted the nucleocapsid protein (NP) transcript from a 2009 clinical sample from New York, USA, a high copy transcript resistant to mutation which, when translated, is essential for viral replication.^64,65^ Open-source crRNA design software for Cas13d, a variant with similar crRNA requirements to Cas13a, was used to design spacer sequences.^66–68^ The three spacer sequences with the highest predicted efficacy were selected and combined with the direct repeat (DR) loop sequence for lwCas13a (Figure 3a).^2^

**Figure 3:**
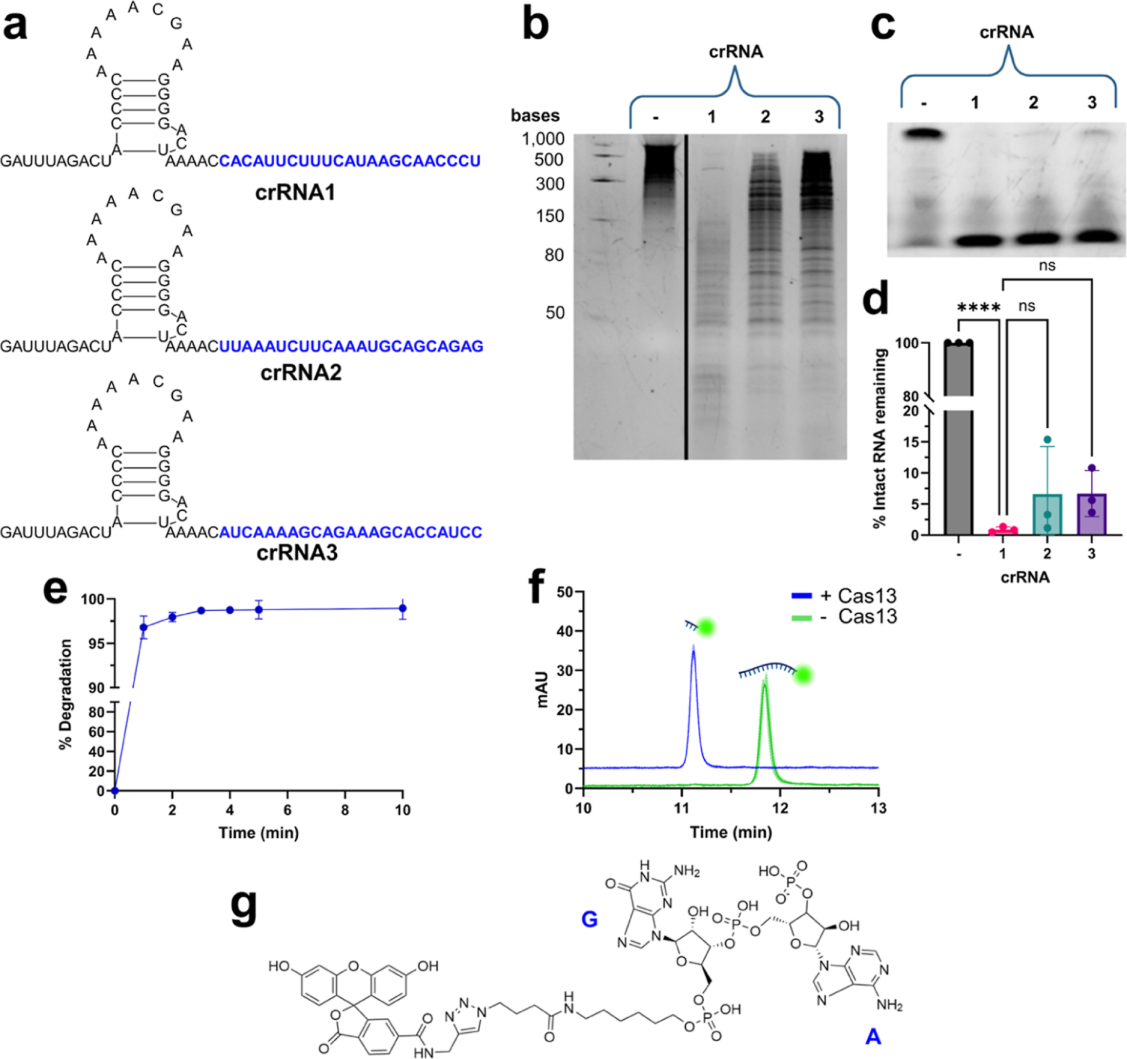
Development and characterization of a CRISPR-Cas13 system against H1N1. (a) crRNA sequences. (b) SYBR Gold stained 15% TBE-urea PAGE gel of degradation of target RNA by Cas13 equipped with three different crRNAs. Black line indicates where gel images have been spliced together. See Figure S52 for full gel. (c) Fluorescence-imaged 15% TBE-urea PAGE gel of conjRNA2 degraded by the collateral activity of activated Cas13 equipped with three different crRNAs. (d) Quantified conjRNA2 degradation by gel images. Quantification performed with ImageJ. *N* = 3. A one-way ANOVA revealed that there was a statistically significant difference in % intact RNA between at least two groups (*F*(3, 8) = [376.5], *p* < 0.0001). Dunnet’s Test for multiple comparisons found that the mean value of % intact RNA was significantly different between the negative control and crRNA1 (*p* < 0.0001, 95% C.I. = [−109.1, −89.10]). No statistical difference was found between crRNA1 and crRNA2 (*p* = 0.3012, C.I. = [−15.72, 4.307]) or between crRNA1 and crRNA3 (*p* = 0.2923, C.I. = [−15.80, 4.229]). (e) Quantified time-dependent degradation of conjRNA2 by Cas13 equipped with crRNA1. Quantification of gel images performed with ImageJ. *N* = 3. (f) RP-HPLC chromatograms (absorbance λ = 498 nm) of conjRNA2 degradation by Cas13 equipped with crRNA1. *N* = 2. Error shown by transparent shaded regions. (g) Identity of conjRNA2 5′ degradation product by HRMS. Letters in blue indicate nucleobase identities. Created in BioRender. Bernardes, G. (2025) https://BioRender.com/l61a423.

As an initial test of the system, the H1N1 target transcript was treated with Cas13 equipped with various crRNAs and analyzed by denaturing PAGE analysis. All crRNAs induced degradation of the target, with **crRNA1** causing the greatest signal depletion (Figure 3b). All Cas13-crRNA systems also produced smearing bands of RNA down the length of the gel, a clear indicator that collateral activity is present.

To test whether our conjugate RNA, **conjRNA1**, would be degraded by the collateral activity of Cas13, we generated a fluorescent version with a 5′ FAM, **conjRNA2** (Figure S2), and incorporated it into the same assays. By gel, degradation of **conjRNA2** was readily seen using all Cas13-crRNA pairs, with **crRNA1** demonstrating >99% depletion (Figure 3c-d). Pleasingly, Cas13 equipped with **crRNA1** rapidly degraded >95% of **conjRNA2** in just 1 min at room temperature (Figure 3e).

While the site of RNA cleavage is unimportant for fluorophore-quenched RNA reporters, as any RNA cleavage will yield a fluorescent signal, the site of cleavage is vitally important for our system, as at least one nucleotide will remain on the LHD101A mutants following cleavage. While a clear degradation product of **conjRNA2** could be seen by gel electrophoresis, its size could not be precisely determined. Thus, to identify the 5′ product of cleavage, analytical reverse-phase high-performance liquid chromatography (RP-HPLC) studies were performed (Figure 3f). The 5′ fluorescent cleavage product appeared as a single Gaussian peak with an earlier retention time relative to the intact RNA, matching expectations, and high-resolution mass spectrometry (HRMS) revealed the product peak contained the 5′ fluorophore attached to 2 nucleotides (Figures 3g and S91). We were optimistic that the degradation of a 17mer intact RNA to a 2mer RNA fragment would be sufficient to recover heterodimer binding for our sensor system.

### Characterization of SOLUNAR with CRISPR-Cas13

Combining **BC10** with the H1N1-targeting Cas13 system, we developed SOLUNAR. We first evaluated the degradation of the RNA conjugated on **BC10** by Cas13 using SDS-PAGE analysis. Here, we found that the Cas13-treated conjugate dropped significantly in molecular weight, nearly to the molecular weight of **BC09**, the E62C-L1 conjugate (Figure 4a). This aligned well with our findings that only two nucleotides remain after Cas13 cleavage (Figure 3g), considering that the negatively charged backbone may also contribute to the migration of the band.

**Figure 4:**
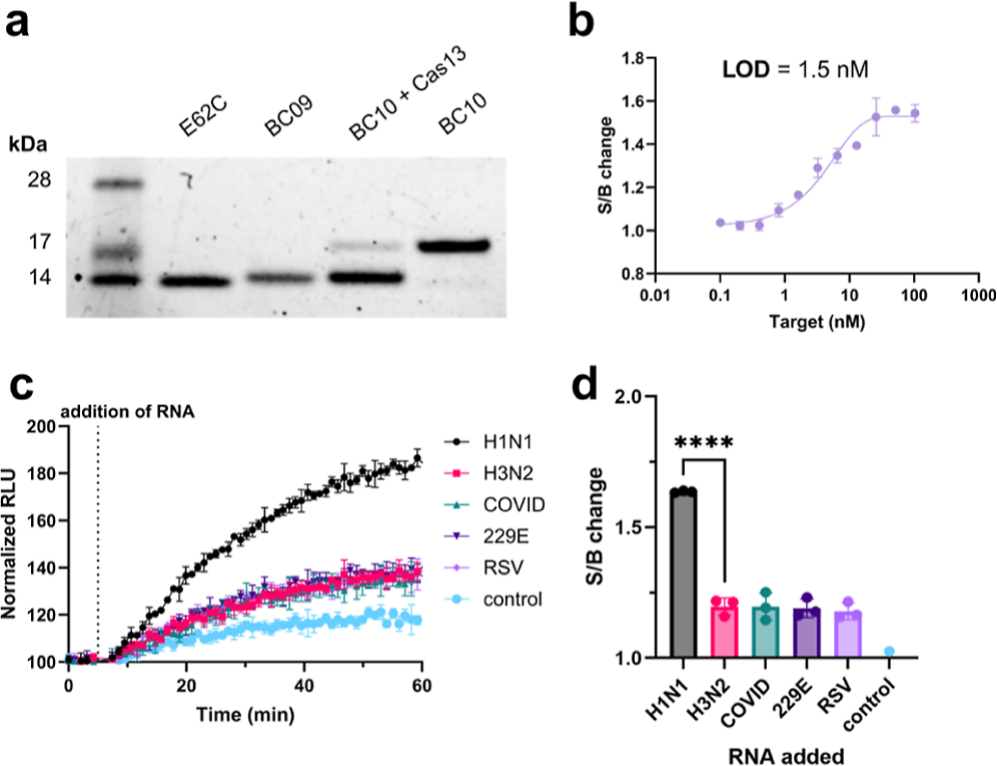
Characterization of SOLUNAR. (a) InstantBlue-stained SDS-PAGE gel of BC10 treated with Cas13 system. (b) Sensor S/B change relative to concentration of target RNA added. *N* = 3. The LOD was determined from the linear regression of the middle 7 points. (c,d) Sensor selectivity for H1N1 over other viral NP transcripts. *N* = 3. Luminescence data normalized to baseline signal, defined as average signal before addition of RNA, (c) and calculated S/B changes (d). A one-way ANOVA revealed that there was a statistically significant difference in S/B change between at least two groups (*F*(5, 12) = [118.6], *p* < 0.0001). Dunnet’s Test for multiple comparisons found that the mean value of S/B was significantly different between H1N1 and H3N2 (*p* < 0.0001, 95% C.I. = [0.3593, 0.5188]). Created in BioRender. Bernardes, G. (2025) https://BioRender.com/e47e494.

To assess sensor efficacy in luminescence assays, **BC10** was treated with Cas13 with various concentrations of activating target RNA. Briefly, in a 96-well plate, 2.5 nM **BC10** and 2.5 nM B protein were treated with preincubated (10 min, RT) 100 nM Cas13 and 50 nM **crRNA1** in 1× HBS-EP+ buffer (Cytiva). 25 min following the addition of 50 μM LuxSit Pro Substrate, luminescence was recorded via plate reader (1 min increments). Different amounts of the target sequence were manually added after 5 min of baseline acquisition, and data was collected for an additional hour at room temperature. S/B changes were evaluated following 1 h of data collection for all conditions.

SOLUNAR demonstrated a dose-dependent response to varying concentrations of target RNA, yielding an LOD of 1.5 nM (9 × 10^8^ copies/μL) (Figure 4b). While altering the concentrations of A and B protein in these assays could not lower the LOD further, single digit nanomolar LODs were produced from all conditions, highlighting the robustness of our technique (Figures S82 and S83). Although attomolar sensitivity is often required for diagnostic use,^69–71^ given that we have not used a preamplification step, a nanomolar LOD is not unexpected nor unreasonable. Enhanced sensitivity of SOLUNAR could potentially be achieved by introducing a preamplification step,^6,8–10,18,19,72,73^ incorporating a postamplification step,^13,17,20,29^ or enhancing the activity of Cas13 ^3,5,12^ as done by others extensively in the literature.

In further studies, SOLUNAR selectively detected the H1N1 NP transcript over other NP transcripts from viruses which cause similar symptoms including Influenza A H3N2, Sars-CoV-2, hCoV-229E (a strain of the common cold), and respiratory syncytial virus (RSV) (Figure 4c,d). While H1N1 detection was statistically significant compared to all nontarget RNAs (*p* < 0.0001), a slight increase in signal relative to the control (water only) was found for all nontargets. Given the absence of any sequence similarity (with or without mismatches), we suspect this is not Cas13-dependent and may instead be due to a mild intercalating effect from the luciferase substrate, a heterocyclic and hydrophobic molecule with a planar core.^49,74^

Interestingly, the maximum S/B ratio with Cas13 was found to be only 1.6 compared to 2.0 in the RNase studies (Figure 2e). However, there are several factors which may contribute to this. Notably, Cas13 and RNase A are known to have different sequence cleavage preferences,^58,60^ a feature which we clearly visualized by HPLC for **conjRNA2** (Figure S90D,F). Further, Cas13 requires additional components for activity (crRNAs, MgCl_2_, NaCl, etc.) which may contribute to quenching effects. Nonetheless, the 1.6 S/B change was distinct and reproducible.

Moreover, SOLUNAR was found to perform well in samples containing up to 5% serum with unaffected performance (Figure 5). SOLUNAR was also capable of discriminating between samples with and without target (200 ng) in 10% serum, albeit with a reduced S/B change (Figure 5a,g). This is a considerable improvement compared to the original SHERLOCK system, where samples could be spiked with up to 0.29% serum with no effect and up to 2% serum with a significant reduction in signal.^2^ The improved performance of our system highlights the strength of bioluminescence in serum relative to fluorescence.^33^

**Figure 5:**
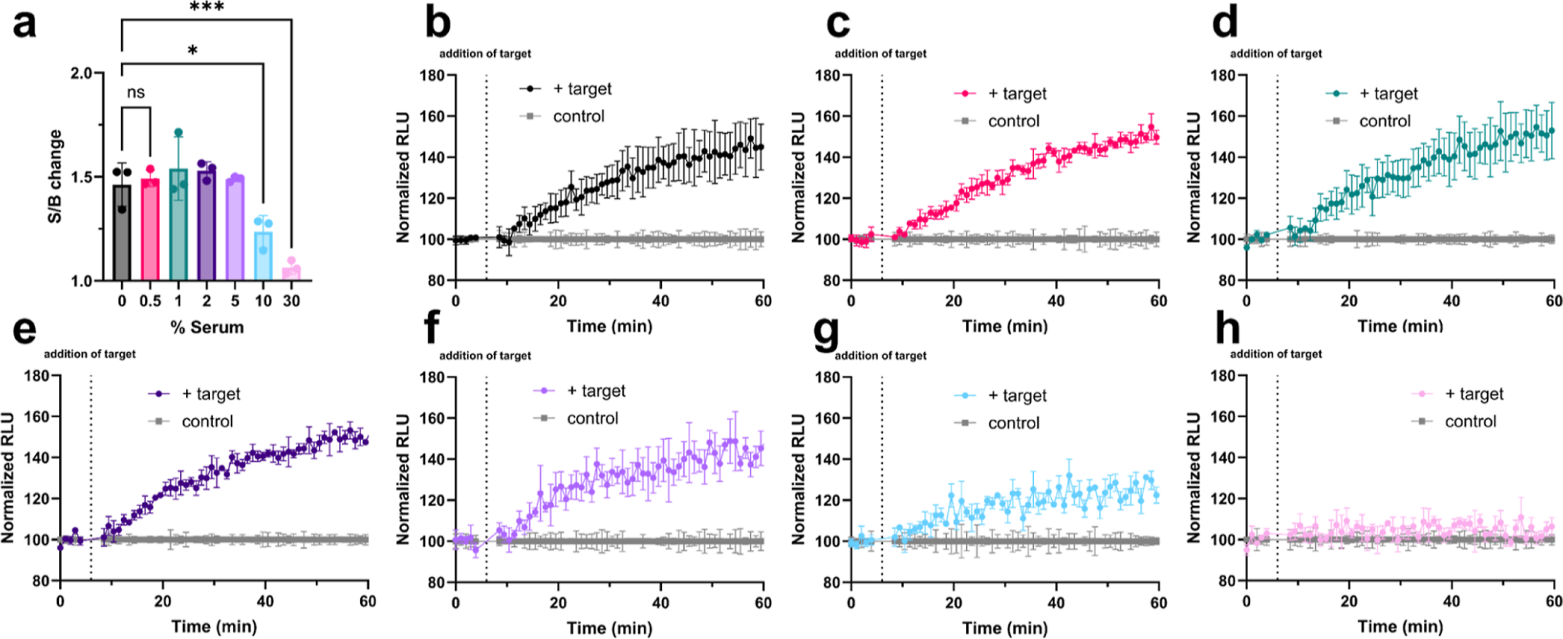
SOLUNAR serum efficacy. (a) Average S/B change of samples containing various percentages of serum. *N* = 3. A one-way ANOVA revealed that there was a statistically significant difference in S/B change between at least two groups (*F*(6, 14) = [15.43], *p* < 0.0001). Dunnet’s Test for multiple comparisons found that the mean S/B value was significantly different between 0% and 30% (*p* < 0.0001, 95% C.I. = [0.2090, 0.5885]) and 0% and 10% (*p* = 0.0172, 95% C.I. = [0.03697, 0.4164]). There was no significant difference between 0% and 0.5% (*p* = 0.9937, 95% C.I. = [-0.2193, 0.1602]). (b–h) Individual time-course luminescence data of SOLUNAR in serum with or without the target RNA (200 ng). *N* = 3 for all sets. (b) 0% serum. (c) 0.5% serum. (d) 1% serum. (e) 2% serum. (f) 5% serum. (g) 10% serum. (h) 30% serum. Created in BioRender. Bernardes, G. (2025) https://BioRender.com/bxaht6t.

An additional strength of SOLUNAR is its compatibility at room temperature during all stages of preparation and use, invoking benefits in point-of-care applications due to ease of operation. To our knowledge, all other published Cas13 sensors require at least one incubation period at 37 °C or higher (Table 1). While these incubations are generally required for preamplification, even amplification-free sensors include this higher temperature for crRNA-Cas13 loading and/or Cas13 cleavage.^3–5,11–14,17,20,24,26–30^ However, our findings reveal that this elevated temperature, while often considered the standard, is unnecessary for SOLUNAR and may also be unnecessary for other amplification-free systems given efficient Cas13-crRNA complexes (Figure 3). Thus, to decrease the LOD of our system, amplification-free techniques would be preferable to preserve room temperature compatibility. Such techniques include combining several crRNAs in a single assay,^3,12^ using a Cas13 variant with enhanced activity,^5^ using tandem CRISPR nucleases,^11^ or incorporation of the system into a microfluidic chip.^14^

**Table 1:** Comparison of Literature-reported Cas13a-based Biosensors

sensor name	preamplification method	LOD (copies/μL)	RNA purification required from serum? (yes/no)	target RNA or DNA	temperature(s) (°C)	readout signal(s)
SOLUNAR (this work)	none	9 × 10^8^	no	Influenza A	20	bioluminescence
SHERLOCK^2,7,72^	RPA	1–2	yes	Dengue, Zika, + Sars-CoV-2	37	fluorescence + LFA
SHERLOCKv2 ^16^	RPA	<1	yes	Dengue + Zika	37	fluorescence + LFA
HUDSON^15^	RPA	10 (urine), ∼1 (saliva), + 45 (blood)	no	West Nile, Yellow Fever, Dengue, + Zika	37–50, 64–95	fluorescence + LFA
SHINE^6^	RPA	5 (saliva)	no	Sars-CoV-2	37, 40, 70, +95	fluorescence + LFA
SATCAS^8^	SAT	0.060	yes	Sars-CoV-2, Epstein-Barr, + Hepatitis B	37, 42, +60	fluorescence
CARMEN^9^	RPA or PCR	variable, ∼9	yes	all known human-associated viruses	41, 55, 70 + variable PCR conditions	fluorescence
mCas13 ^10^	LAMP	4	yes	Sars-CoV-2	37 + 62	fluorescence
PECL-CRISPR^18^	EXPAR	600	NR^a^	miRNA	37 + 55	electrochemiluminescence
RC-LFA^19^	RPA	10	yes	Human Papillomavirus (HPV)	37	LFA
environmental detection^73^	RPA	10	yes	RNA of *C. carpiio* + *O. latipes*	–15, 37	fluorescence + LFA
SATORI^3^	none	3000	NR^a^	Sars-CoV-2	25 + 37	fluorescence
hydrogel-based^4^	none	60	saliva-compatible	Sars-CoV-2	37 + 95	hydrogel capillary migration distance
RBD-LwaCas13a^5^	none	<1	no (up to 5% serum)	Dengue, Zika, Sars-CoV-2, Ebola, + miRNA	20 + 37	fluorescence + electrochemical
FIND-IT^11^	none	31	yes	Sars-CoV-2	20 + 37	fluorescence
combined crRNA^12^	none	100	yes	Sars-CoV-2	37	fluorescence
autocatalytic-based^13^	none	23	yes	Sars-CoV-2	37	fluorescence
microfluidics-based^14^	none	5.5 × 10^4^	yes	Ebola + Marburg	37	fluorescence
Cas13a-bHCR^17^	none	5	no	miRNA	37	SERS
paper-supported PEC^20^	none	46	no	miRNA	20 + 40	photoelectrochemical
aquaculture detection^24^	none	10^6^	yes	Taura syndrome virus	37	fluorescence
CRISPR-chip^26^	none	1.2 × 10^6^	yes	miRNA	37	electrochemical
QD-RHP^27^	none	6 × 10^7^	yes	*lcrV*transcriptof *Y. pestis*	25 + 37	fluorescence
passivating quantum dots^28^	none	NR^a^	yes	35 nt model RNA	37	fluorescence
MnO_2_ nanosheet approach^29^	none	2 × 10^5^	no	miRNA	37	fluorescence + LFA
NiCo_2_O_4_ nanozyme^30^	none	1.4 × 10^3^	NR^a^	miRNA +lncRNA	37	fluorescence + colorimetric

a Data not reported^4,17,20,29^ Table 1).^6,15^

Among the various strengths of SOLUNAR, there also remain several opportunities for sensor optimization in future generations. The sensitivity of the sensor relies on several key factors including: (i) efficient blocking of A and B when A is conjugated to intact RNA, (ii) efficient binding of A and B post RNA cleavage, and (iii) a significant difference in binding affinities between the LHD101 and LuxSit s-Pro components. Potential strategies to increase the blocking effect (i) include increasing the length of or introducing secondary structure to the sensor RNA, functionalizing the 3′ end of the sensor RNA with something bulky (a peptide, protein, etc.), or conjugating RNA to both proteins A and B. Opportunities to improve efficient binding postcleavage (ii) include further exploration of the chemical space of the linker to investigate sensor preferences and altering the sequence composition of the sensor RNA. Alternatively, use of a protein pair with even higher affinity (iii) than LHD101 (nM) and/or lower affinity of LuxSit s-Pro (μM) could theoretically decrease the background of the system. These potential alterations must be weighed in the context of all three factors but may enable even more sensitive detection for future sensing applications.

## Conclusions

Here, we report the development of SOLUNAR, a novel Cas13-based biosensor using a protein-RNA bioconjugate. Unlike previously developed systems, SOLUNAR utilizes a robust de novo luciferase, allowing for detection in ≤10% serum. Despite a modest LOD of 1.5 nM, SOLUNAR is fully functional at room temperature, and this LOD could potentially be reduced in the future using various techniques as explored by others.^3,5,12^ Although only tested with H1N1 in this study, SOLUNAR could be readily applied to any RNA-based virus or microRNA (miRNA) of interest through the simple design of a single crRNA per target. The SOLUNAR platform could also be readily translated for the detection of DNA using Cas12 by swapping the sensor RNA modification for DNA,^16^ which may improve the performance of the system due to the bulk of DNA relative to RNA.

To our knowledge, only a few Cas13 diagnostics achieve direct analysis in serum, four which require material-based approaches^4,17,20,29^ and two which require high temperature (>60 °C) incubations (Table 1).^6,15^ Thus, our serum-compatibility at room temperature with a simple luciferase uniquely reduces the need for a prepurification step, simplifying testing and preventing cross-contamination. As a one-pot, room-temperature compatible system which requires only an appropriate photon detector, SOLUNAR shows great promise in point-of-care applications.

Beyond diagnostic applications, SOLUNAR could be utilized for the quantitative detection of sequence-specific RNA, with potential applications in gene expression analysis and RNA purification. Further, due to the modular design of the platform presented here, we envisage SOLUNAR could have additional applications in activity-based enzymatic sensing for enzymes beyond nucleases. Simply by post-translationally substituting the RNA modification with other enzyme substrates, SOLUNAR could be redesigned to detect other enzymes which cleave bonds and may demonstrate superior performance. Given the success of SOLUNAR when paired with Cas13, we believe that this platform allows for the flexible and precise design of biosensors, highlighting the strengths of both bioconjugation and de novo protein design.

## Supplementary Material



## Notes

Detailed methods, protein and RNA sequences, characterization data, and additional figures (PDF)

## ∥

A-Alpha Bio, Seattle, WA 98121 U.S.A

## Abbreviations

BRET: bioluminescence resonance energy transfer
CRISPR: clustered regularly interspaced short palindromic repeats
crRNA: CRISPR RNA
CuAAC: copper-catalyzed azide–alkyne cycloaddition
*E. coli*: *Escherichia coli*
EXPAR: exponential amplification reaction
DBCO: dibenzocyclooctyne
DR: direct repeat
FAM: fluorescein
HA: hemagglutinin
HRMS: high-resolution mass spectrometry
LAMP: loop-mediated isothermal amplification
LC–MS: liquid chromatography–mass spectrometry
LFA: lateral flow assay
lncRNA: long noncoding RNA
LOD: limit of detection
lwCas13a: Cas13a from *Leptotrichia wadei*
miRNA: microRNA
NP: nucleocapsid protein
nt: nucleotide(s)
PAE: predicted aligned error
PAGE: polyacrylamide gel electrophoresis
PAM: protospacer adjacent motif
pI: isoelectric point
pLDDT: per-residue local-distance difference test
RPA: recombinase polymerase amplification
RP-HPLC: reverse-phase high-performance liquid chromatography
RT: room temperature
RT-RPA: reverse transcription-recombinase polymerase amplification
S/B: signal-to-baseline
SAT: simultaneous amplification and testing
SDS-PAGE: sodium dodecyl sulfate-polyacrylamide gel electrophoresis
SEC: size exclusion chromatography
SERS: surface-enhanced Raman spectroscopy
SHERLOCK: Specific High-sensitivity Enzymatic Reporter unLOCKing
SOLUNAR: Selective One-pot LUminescent Nucleic Acid Reporter
SPAAC: strain-promoted azide–alkyne cycloaddition
ssRNA: single-stranded RNA
THPTA: tris-hydroxypropyltriazoylmethylamine
U: uracil
