# Supplementary material for: A De Novo Luciferase Bioconjugate for the Cas13-Based Detection of Influenza A
Source: JACS Au. 2025 Jul 28;5(8):3914–25. doi: 10.1021/jacsau.5c00576 (PMC12381745; doi:10.1021/jacsau.5c00576)
Supplement: Supplementary file 1 [file au5c00576_si_001.pdf]

Supporting Information

A *De novo* Luciferase Bioconjugate for the Cas13-based Detection of Influenza A

Mary Canzano,[‡] Joseph L. Harman,[ϕ] Yanira Méndez,[‡] Alfredo Quijano-Rubio,[ϕ,*] Daniel-Adriano Silva,[ϕ,*] and Gonçalo J. L. Bernardes[‡,Ω,*]

[‡]Yusuf Hamied Department of Chemistry, University of Cambridge, Lensfield Road, CB2 1EW Cambridge, UK

[ϕ]Monod Bio Incorporated, 700 Dexter Ave N, Suite 700, Seattle, Washington 98109, USA

[Ω]Translational Chemical Biology Group, Spanish National Cancer Research Centre (CNIO), Madrid 28029, Spain

*corresponding authors:

Gonçalo J. L. Bernardes: gb453@cam.ac.uk, Alfredo Quijano-Rubio: aquijano@monod.bio, Daniel-Adriano Silva: dadriano@monod.bio

Contents

1. Experimental procedures 8
1.1 General biology procedures 8
1.2 General chemical procedures 8
1.3 General LC-MS procedure for analysis of proteins 9
1.4 Analytical HPLC procedure for analysis of RNA 9
1.5 Protein expression & purification 9
1.6 Protein luminescence characterization 10
1.7 Bioconjugate library construction 10
1.7.1 BC01 (E60C-L1) 11
1.7.2 BC02 (E60C-L1-RNA) 11
1.7.3 BC03 (E60C-L2) 11
1.7.4 BC04 (E60C-L2-RNA) 11
1.7.5 BC05 (I61C-L1) 11
1.7.6 BC06 (I61C-L1-RNA) 11
1.7.7 BC07 (I61C-L2) 12
1.7.8 BC08 (I61C-L2-RNA) 12
1.7.9 BC09 (E62C-L1) 12
1.7.10 BC10 (E62C-L1-RNA) 12
1.7.11 BC11 (E62C-L2) 12
1.7.12 BC12 (E62C-L2-RNA) 12
1.7.13 BC13 (G65C-L1) 13
1.7.14 BC14 (G65C-L1-RNA) 13
1.7.15 BC15 (G65C-L2) 13
1.7.16 BC16 (G65C-L2-RNA) 13
1.7.17 BC17 (BH-E62C-L1) 13
1.7.18 BC18 (BH-E62C-L1-RNA) 13
1.7.19 Conjugate identities 14
1.8 Screening of conjugate library with RNase A 14

1.9 Viral transcript library construction 15
1.10 crRNA design 15
1.11 conjRNA2 preparation 15
1.12 Cas13 crRNA efficacy screen 16
1.13 Cas13 time trial 16
1.14 Cas13 degradation of BC10 by SDS-PAGE 16
1.15 Sensor matrix screen 16
1.16 Sensor limit of detection (LOD) for target RNA 17
1.17 Sensor selectivity for target RNA 18
1.18 Sensor efficacy in serum 18
1.19 Long-term luminescence of BC10 19
1.20 Substrate solvent preference for sensor 19
1.21 Effect of RNase inhibitor on sensor signal 19
1.22 Linker Synthesis 20
1.22.1 1, *tert*-butyl (4-(2,5-dioxo-2,5-dihydro-1*H*-pyrrol-1-yl)butyl)carbamate 20
1.22.2 2, *tert*-butyl (2-(2-(2-(2,5-dioxo-2,5-dihydro-1*H*-pyrrol-1-yl)ethoxy)ethoxy)ethyl)carbamate 21
1.22.3 3, 1-(4-aminobutyl)-1*H*-pyrrole-2,5-dione 21
1.22.4 4, 1-(2-(2-(2-aminoethoxy)ethoxy)ethyl)-1*H*-pyrrole-2,5-dione 22
1.22.5 5, Linker 1 (L1) 22
1.22.6 6, Linker 2 (L2) 23
2. Protein, DNA, and RNA sequences 24
2.1 Protein Sequences 24
2.1.1 E60C 24
2.1.2 I61C 24
2.1.3 E62C 24
2.1.4 G65C 24
2.1.5 AWT 24
2.1.6 BWT 24
2.1.7 BH-E62C 24

2.1.8 BH-AWT 24
2.1.9 BH-BWT 25
2.1.10 Extinction coefficients, molecular weights, and isoelectric points (pI) 25
2.2 Viral Sequences: 25
2.2.1 H1N1 NP template (Target RNA) 25
2.2.2 H3N2 NP template 26
2.2.3 COVID NP template 26
2.2.4 hCoV-229E NP template 27
2.2.5 RSV NP template 28
2.2.6 Shortened H1N1 NP RNA (short Target RNA) 28
2.3 crRNA sequences 28
2.3.1 crRNA1 28
2.3.2 crRNA2 28
2.3.3 crRNA3 28
2.4 Conjugate RNA 29
2.4.1 conjRNA1 29
2.4.2 conjRNA2 29
3. LC-MS Spectra 30
3.1 LC-MS Spectra of Proteins 30
3.1.1 E60C LC-MS 30
3.1.2 I61C LC-MS 31
3.1.3 E62C LC-MS 32
3.1.4 G65C LC-MS 33
3.1.5 AWT LC-MS 34
3.1.6 BWT LC-MS 35
3.1.7 BH-E62C LC-MS 36
3.1.8 BH-AWT LC-MS 37
3.1.9 BH-BWT LC-MS 38
3.2 LC-MS spectra of bioconjugates 39

3.2.1 BC01 (E60C-L1) LC-MS 39
3.2.2 BC02 (E60C-L1-RNA) LC-MS 40
3.2.3 BC03 (E60C-L2) LC-MS 41
3.2.4 BC04 (E60C-L2-RNA) LC-MS 42
3.2.5 BC05 (I61C-L1) LC-MS 43
3.2.6 BC06 (I61C-L1-RNA) LC-MS 44
3.2.7 BC07 (I61C-L2) LC-MS 45
3.2.8 BC08 (I61C-L2-RNA) LC-MS 46
3.2.9 BC09 (E62C-L1) LC-MS 47
3.2.10 BC10 (E62C-L1-RNA) LC-MS 48
3.2.11 BC11 (E62C-L2) LC-MS 49
3.2.12 BC12 (E62C-L2-RNA) LC-MS 50
3.2.13 BC13 (G65C-L1) LC-MS 51
3.2.14 BC14 (G65C-L1-RNA) LC-MS 52
3.2.15 BC15 (G65C-L2) LC-MS 53
3.2.16 BC16 (G65C-L2-RNA) LC-MS 54
3.2.17 BC17 (BH-E62C-L1) LC-MS 55
3.2.18 BC18 (BH-E62C-L1-RNA) LC-MS 56
4. Size exclusion chromatography (SEC) of protein-RNA bioconjugates 57
4.1 BC02 (E60C-L1-RNA) SEC trace 57
4.2 BC04 (E60C-L2-RNA) SEC trace 57
4.3 BC06 (I61C-L1-RNA) SEC trace 58
4.4 BC08 (I61C-L2-RNA) SEC trace 58
4.5 BC10 (E62C-L1-RNA) SEC trace 59
4.6 BC12 (E62C-L2-RNA) SEC trace 59
4.7 BC14 (G65C-L1-RNA) SEC trace 60
4.8 BC16 (G65C-L2-RNA) SEC trace 60
4.9 BC18 (BH-E62C-L1-RNA) SEC trace 61
5. Gel electrophoresis 62

5.1 SDS-PAGE characterization of proteins 62
5.1.1 Gel of E60C and AWT purifications 62
5.1.2 Gel of BWT purification 63
5.1.3 Gel of I61C purification 63
5.1.4 Gel of E62C purification 64
5.1.5 Gel of G65C purification 64
5.2 SDS-PAGE characterization of protein-RNA bioconjugates 65
5.2.1 Gel of BC06 (I61C-L1-RNA) 65
5.2.2 Gel of BC08 (I61C-L2-RNA) 65
5.2.3 Gel of BC10 (E62C-L1-RNA) 66
5.2.4 Gel of BC12 (E62C-L2-RNA) 66
5.2.5 Gel of BC02 (E60C-L1-RNA), BC04 (E60C-L2-RNA), BC14 (G65C-L1-RNA), and BC16 (G65C-L2-RNA) 67
5.2.6 Gel of BC18 (BH-E62C-L1-RNA) 67
5.3 SDS-PAGE characterization of Cas-13 degradation of BC10 68
5.4 RNA denaturing PAGE 68
5.4.1 Viral transcripts 68
5.4.2 crRNA efficacy screen 69
5.4.3 Cas13 time trial 70
6. Luminescence Data 72
6.1 Luminescence data from pure, unmodified proteins 72
6.1.1 E60C raw signal 72
6.1.2 I61C, E62C, and G65C raw signal 73
6.1.3 Mutant signals normalized to wild type 74
6.1.4 BH-E62C raw signal 74
6.1.5 BH-E62C signal normalized to wild type 75
6.2 Luminescence data from RNase A screen 76
6.2.1 BC02 raw luminescence with RNase 76
6.2.2 BC04 raw luminescence with RNase 77
6.2.3 BC06 raw luminescence with RNase 78

6.2.4 BC08 raw luminescence with RNase 79
6.2.5 BC10 raw luminescence with RNase 80
6.2.6 BC12 raw luminescence with RNase 81
6.2.7 BC14 raw luminescence with RNase 82
6.2.8 BC16 raw luminescence with RNase 83
6.2.9 BC18 raw luminescence with RNase 84
6.2.10 Combined analysis of RNase screen 85
6.3 Matrix screen 87
6.3.1 BC10 matrix raw data 87
6.3.2 BC18 matrix screen 91
6.4 Sensor Limit of Detection (LOD) for target RNA 93
6.4.1 LOD using 2.5 nM BC10 and 2.5 nM BWT 93
6.4.2 LOD using 10 nM BC10 and 5 nM BWT 94
6.4.3 LOD using 10 nM BC10 and 0.75 nM BWT 95
6.5 Viral transcript selectivity 96
6.6 Sensor efficacy in serum 97
6.7 Long-term luminescence of BC10 98
6.8 Substrate solvent preference for sensor 98
6.9 Effect of RNase inhibitor on sensor signal 99
7. Analytical HPLC 100
8. NMR 102
9. References 108

1. Experimental procedures

1.1 General biology procedures

All buffers and solutions were prepared using Ultrapure Milli-Q (MQ) water and were vacuum filtered using Millipore Express PLUS 0.22 μm filter paper (Sigma).

All SDS-PAGE gels were run using NuPAGE™ Bis-Tris Mini Protein Gels, 4–12%, 1.0–1.5 mm (Thermo Fisher) and 1X (from 20X stock) NuPAGE™ MES SDS Running Buffer (Thermo Fisher). Samples were prepared with 1X (from 4X stock) NuPAGE™ LDS sample buffer (Thermo Fisher) and 1X (from 10X stock) NuPAGE™ Sample Reducing Agent (Thermo Fisher) and were boiled at 80 °C for 5–10 minutes prior to gel loading. Gels were run for 30–45 minutes at 200 V and stained with InstantBlue (Abcam). The SeeBlue™ Plus2 pre-stained protein ladder (Invitrogen) was used with all samples

All RNA denaturing page gels were run using Novex™ TBE-Urea gels, 15% (Invitrogen) and 1X TBE buffer prepared from a 10X solution made from tris base (Merck), boric acid (Merck), and ethylenediaminetetraacetic acid (EDTA) tetrasodium salt tetrahydrate (Fisher Scientific). Samples were prepared with 1X (from 2X stock) Novex™ TBE-Urea sample buffer (Invitrogen) and were boiled at 80 °C for 10 minutes prior to gel loading. Gel wells were thoroughly washed with running buffer to remove excess urea prior to gel loading. Gels were run for 45–75 minutes at 180 V. The low range ssRNA ladder (New England Biolabs) was used.

1.2 General chemical procedures

Dry solvents (dichloromethane) were prepared in-house. All reagents were purchased from commercial suppliers and used without further purification. LC-MS hypergrade acetonitrile (ACN), LC-MS grade formic acid and MQ water were used to prepare mobile phases for LC-MS analysis. HPLC grade ACN, HPLC grade triethylammonium acetate (TEAA) (Merck), and MQ were used to prepare mobile phases for analytical HPLC analysis. ^{1}H-NMR and ^{13}C-NMR were recorded using a Bruker 400 MHz Avance II HD Smart Probe spectrometer.

MestreNova (ver. 14.3.1) was used to process all NMR raw data. Thin-layer chromatography (TLC) was performed using silica gel 60 on glass with indicator F_{254} (Merck) and Preparative TLC (Prep-TLC) was performed using Silica gel GF, UV 254, 20 × 20 cm, 1500 micron (Uniplate™).

1.3 General LC-MS procedure for analysis of proteins

Protein samples were analyzed with a Waters SQD2 mass spectrometer with an Acquity UPLC BEH300 C4 column (130 Å 1.7 µm, 2.1 x 50 mm) using an Acquity UPLC system. Mobile phase A contained 99.9% MQ and 0.1% formic acid, and mobile phase B contained 99.9% ACN with 0.1% formic acid. The following gradient was used for all samples with a constant flow rate of 0.2 mL/min: 5–72% B (0–6 minutes), 72% B (6–7.5 minutes), 72–5% B (7.5–7.75 minutes), 5% B (7.75–9 minutes). Nitrogen was used as the desolvation gas at a flow rate of 800 L h^{-1}, and a capillary voltage of 3.0 kV was maintained for the electrospray source and a cone voltage of 30 V. The column temperature was kept at 40 °C. The ion series were first deconvoluted with the MaxEnt1 algorithm on the MassLynx software (ver. 4.1). Further deconvolution was performed in UniDec (ver. 6.0.4),[1] and all data was plotted in GraphPad Prism (ver. 10.4.1).

1.4 Analytical HPLC procedure for analysis of RNA

Analytical reverse phase HPLC (RP-HPLC) was performed on an Agilent 1260 Infinity system equipped with an ACE 5 C18-300 column (5.0 µm, 150 x 2.1 mm). Mobile phase A contained 100 mM TEAA in MQ, and mobile phase B contained 100 mM TEAA in 80% ACN and 20% MQ. The following gradient was used for all samples with a constant flow rate of 0.4 mL/min: 5–40% B (0–30 mins), 40–100% B (30–31 mins), 100% B (31–36 mins), 100–5% B (36–37 mins), 5% B (37–45 mins). Absorbance data was collected for 260, 280, and 498 nm. 5–20 µL injections were used. The column temperature was kept at 40 °C.

1.5 Protein expression & purification

Plasmids encoding all proteins were transformed into BL21 (DE3) *E. coli* competent cells (Thermo Fisher) via standard heat shock protocols. Single colony cultures were grown in 10 mL of LB media supplemented with 50 µg/mL Kanamycin overnight while shaking at 180 rpm and 37 °C for approximately 16 hours. To prewarmed 1 L flasks of 2xYT media supplemented with 50 µg/mL Kanamycin, all 10 mL of the single colony culture were added. The culture was allowed to grow while shaking at 200 rpm at 37 °C until reaching an OD of 0.6–0.8 (approximately 3–4 hours). Then, the cultures were induced with the addition of 1 mM Isopropyl β-D-1-thiogalactopyranoside (IPTG) (final) and allowed to grow for 16 hours at 18 °C. The cultures were harvested via centrifugation at 8,000 rpm for 30 minutes at 4 °C (~3–4 grams pellet/L media)

The cell pellet was lysed by three consecutive freeze-thaws with dry ice and a 37 °C water bath. Following this, the cells were further lysed with the addition of 20 mL lysis buffer (1X PBS pH 7.4, 500 mM NaCl, 1 mM DTT supplemented with DNase I and protease inhibitor (cOmplete™, Mini, EDTA-free protease inhibitor cocktail (Roche)) and sonicated for 5

minutes, amplitude 60, 20 seconds on and 40 seconds off. The lysate was harvested via centrifugation at 18,000 rpm for 30 minutes at 4 °C.

The lysate (20 mL) was incubated with 1 mL of Ni Sepharose™ Excel (Cytiva) resin for one hour at room temperature while rolling. The protein was then purified via gravity flow with the addition of 1X PBS pH 7.4 supplemented with increasing concentrations of imidazole (20 mM: 5 CVs, 40 mM: 5 CVs, 250 mM: 1 CV, and 500 mM: 5 CV). Elution fractions were pooled and concentrated. Samples were either dialyzed overnight in 1X PBS pH 7.4 at 4 °C using Slide-a-Lyzer™ dialysis cassettes (3.5 MWCO) (Thermo Scientific), desalted using HiPrep™ 26/10 Desalting column (Cytiva) using 1X PBS pH 7.4, or loaded directly onto a Superdex™ 75 Increase 10/300 GL (Cytiva) and purified via Äkta* fast protein liquid chromatography (FPLC). Dialyzed or desalted samples were also purified by size exclusion. Sample purity was confirmed via SDS-PAGE. Samples were flash frozen in liquid nitrogen and stored at -80 °C. Protein yield was ~ 0.5–5 mg pure / L media.

*Note, for any use of an Äkta system with these proteins (especially Äkta's which are shared with others), we recommend flushing the entire system (including the fraction collector) extensively with 0.5–1 M NaOH to attempt to avoid RNase contamination. We also washed the Superdex column with 0.5 M NaOH followed by RNase free H_2O prior to equilibrating with buffer for the same reason. If proteins do acquire RNase contamination, a second round of IMAC appeared to sufficiently remove the nuclease.

1.6 Protein luminescence characterization

Directly following purification, all proteins were screened for luminescence activity. To a white-bottomed 96 well plate (Corning, CLS3605), protein A was added (45 µL of 20 nM, 0.9 pmol) to a final concentration of 9 nM. Protein B was added to a final concentration of 9 nM (45 µL of 20 nM, 0.9 pmol). To this, the LuxSit Pro substrate (Fisher Scientific, Cat no. NC3136279) was added to a final concentration of 50 µM (10 µL of 500 µM, 5 nmol). The plate was immediately analyzed in the plate reader for 1 hour with 2-minute increments, emission wavelength 300–850 nm. Data was processed using GraphPad Prism (ver. 10.4.1). **Final assay conditions:** 9 nM A, 9 nM B, 50 µM substrate, 1% DMF, LuxSit Pro Assay buffer (Fisher Scientific, Cat no. NC3136279).

1.7 Bioconjugate library construction

For all conjugations, L1 and L2 were dissolved in DMF. The overall final concentration of DMF in each bioconjugation did not exceed 10%.

1.7.1 BC01 (E60C-L1)

To 200 µL of 187 µM E60C (37 nmol), 1 equivalent of tris(2-carboxyethyl)phosphine (TCEP) (3.74 µL of 10 mM, 37 nmol) and 4 equivalents of L1 (7.48 µL of 20 mM, 150 nmol) were added. The sample was allowed to react for 1 hour at room temperature. Complete conversion to **BC01** was confirmed via LC-MS, and the sample was immediately SEC purified. Fractions were pooled and concentrated to yield 270 µL of **BC01** at 93 µM.

1.7.2 BC02 (E60C-L1-RNA)

To 250 µL of 93 µM **BC01** (23 nmol), 10 equivalents of **conjRNA1** were added (23 µL of 10 mM, 230 nmol). The sample was allowed to react overnight at room temperature. Complete conversion to **BC02** was confirmed via LC-MS, and the sample was immediately SEC purified. Sample purity was confirmed via SDS-PAGE.

1.7.3 BC03 (E60C-L2)

To 200 µL of 187 µM E60C (37 nmol), 1 equivalent of TCEP (3.74 µL of 10 mM, 37 nmol) and 4 equivalents of L2 (7.48 µL of 20 mM, 150 nmol) were added. The sample was allowed to react for 1 hour at room temperature. Complete conversion to **BC03** was confirmed via LC-MS, and the sample was immediately SEC purified. Fractions were pooled and concentrated to yield 270 µL of **BC03** at 76 µM.

1.7.4 BC04 (E60C-L2-RNA)

To 250 µL of 76 µM **BC03** (19 nmol), 10 equivalents of **conjRNA1** were added (19 µL of 10 mM, 190 nmol). The sample was allowed to react overnight at room temperature. Complete conversion to **BC04** was confirmed via LC-MS, and the sample was immediately SEC purified. Sample purity was confirmed via SDS-PAGE.

1.7.5 BC05 (I61C-L1)

To 200 µL of 175 µM I61C (35 nmol), 1 equivalent of TCEP (3.5 µL of 10 mM, 35 nmol) and 4 equivalents of L1 (7 µL of 20 mM, 140 nmol) were added. The sample was allowed to react for 1 hour at room temperature. Complete conversion to **BC05** was confirmed via LC-MS, and the sample was immediately SEC purified. Fractions were pooled and concentrated to yield 240 µL of **BC05** at 52 µM.

1.7.6 BC06 (I61C-L1-RNA)

To 220 µL of 52 µM **BC05** (11 nmol), 9 equivalents of **conjRNA1** were added (10 µL of 10 mM, 100 nmol). The sample was allowed to react overnight at room temperature. Complete conversion to **BC06** was confirmed via LC-MS, and the sample was immediately SEC purified. Sample purity was confirmed via SDS-PAGE.

1.7.7 BC07 (I61C-L2)

To 200 µL of 175 µM I61C (35 nmol), 1 equivalent of TCEP (3.5 µL of 10 mM, 35 nmol) and 4 equivalents of L2 (7 µL of 20 mM, 140 nmol) were added. The sample was allowed to react for 1 hour at room temperature. Complete conversion to **BC07** was confirmed via LC-MS, and the sample was immediately SEC purified. Fractions were pooled and concentrated to yield 160 µL of **BC07** at 82 µM.

1.7.8 BC08 (I61C-L2-RNA)

To 140 µL of 82 µM **BC07** (11 nmol), 9 equivalents of **conjRNA1** were added (10 µL of 10 mM, 100 nmol). The sample was allowed to react overnight at room temperature. Complete conversion to **BC08** was confirmed via LC-MS, and the sample was immediately SEC purified. Sample purity was confirmed via SDS-PAGE.

1.7.9 BC09 (E62C-L1)

To 150 µL of 140 µM E62C (21 nmol), 1 equivalent of TCEP (2.1 µL of 10 mM, 21 nmol) and 4 equivalents of L1 (4.2 µL of 20 mM, 84 nmol) were added. The sample was allowed to react for 1 hour at room temperature. Complete conversion to **BC09** was confirmed via LC-MS, and the sample was immediately SEC purified. Fractions were pooled and concentrated to yield 270 µL of **BC09** at 17.5 µM.

1.7.10 BC10 (E62C-L1-RNA)

To 250 µL of 17.5 µM **BC09** (4.4 nmol), 11 equivalents of **conjRNA1** were added (5 µL of 10 mM, 50 nmol). The sample was allowed to react overnight at room temperature. Complete conversion to **BC10** was confirmed via LC-MS, and the sample was immediately SEC purified. Sample purity was confirmed via SDS-PAGE.

1.7.11 BC11 (E62C-L2)

To 200 µL of 148 µM E62C (30 nmol), 1 equivalent of TCEP (3 µL of 10 mM, 30 nmol) and 6 equivalents of L2 (8.9 µL of 20 mM, 177 nmol) were added. The sample was allowed to react for 1 hour at room temperature. Complete conversion to **BC11** was confirmed via LC-MS, and the sample was immediately SEC purified. Fractions were pooled and concentrated to yield 270 µL of **BC11** at 65 µM.

1.7.12 BC12 (E62C-L2-RNA)

To 250 µL of 65 µM **BC11** (16 nmol), 10 equivalents of **conjRNA1** were added (16.2 µL of 10 mM, 160 nmol). The sample was allowed to react overnight at room temperature. Complete conversion to **BC12** was confirmed via LC-MS, and the sample was immediately SEC purified. Sample purity was confirmed via SDS-PAGE.

1.7.13 BC13 (G65C-L1)

To 200 µL of 190 µM G65C (38 nmol), 1 equivalent of TCEP (3.8 µL of 10 mM, 38 nmol) and 4 equivalents of L1 (7.6 µL of 20 mM, 152 nmol) were added. The sample was allowed to react for 1 hour at room temperature. Complete conversion to **BC13** was confirmed via LC-MS, and the sample was immediately SEC purified. Fractions were pooled and concentrated to yield 270 µL of **BC13** at 80 µM.

1.7.14 BC14 (G65C-L1-RNA)

To 250 µL of 80 uM **BC13** (20 nmol), 10 equivalents of **conjRNA1** were added (20 µL of 10 mM, 200 nmol). The sample was allowed to react overnight at room temperature. Complete conversion to **BC14** was confirmed via LC-MS, and the sample was immediately SEC purified. Sample purity was confirmed via SDS-PAGE.

1.7.15 BC15 (G65C-L2)

To 200 µL of 190 µM G65C (38 nmol), 1 equivalent of TCEP (3.8 µL of 10 mM, 38 nmol) and 4 equivalents of L2 (7.6 µL of 20 mM, 152 nmol) were added. The sample was allowed to react for 1 hour at room temperature. Complete conversion to **BC15** was confirmed via LC-MS, and the sample was immediately SEC purified. Fractions were pooled and concentrated to yield 270 µL of **BC15** at 68 µM.

1.7.16 BC16 (G65C-L2-RNA)

To 250 µL of 68 µM **BC15** (17 nmol), 10 equivalents of **conjRNA1** were added (17 µL of 10 mM, 170 nmol). The sample was allowed to react overnight at room temperature. Complete conversion to **BC16** was confirmed via LC-MS, and the sample was immediately SEC purified. Sample purity was confirmed via SDS-PAGE.

1.7.17 BC17 (BH-E62C-L1)

To 300 µL of 129 µM BH-E62C (39 nmol), 1 equivalent of TCEP (3.9 µL of 10 mM, 39 nmol) and 4 equivalents of L1 (7.7 µL of 20 mM, 155 nmol) were added. The sample was allowed to react for 2 hours at room temperature. Complete conversion to **BC17** was confirmed via LC-MS, and the sample was immediately SEC purified. Fractions were pooled and concentrated to yield 200 µL of **BC17** at 65 µM.

1.7.18 BC18 (BH-E62C-L1-RNA)

To 8 equivalents of **conjRNA1** (26 µL of 10 mM, 260 nmol), 4 equivalents of L1 (6.5 µL of 20 mM, 130 nmol) were added on ice and allowed to react for 30 minutes. Then, 1 equivalent of BH-E62C (260 µL of 125 µM, 33 nmol) was added with 1 equivalent of TCEP (3.25 µL of 10 mM, 33 nmol). Complete conversion to **BC18** was confirmed via LC-MS, and the sample was immediately SEC purified. Sample purity was confirmed via SDS-PAGE.

1.7.19 Conjugate identities

Table S1: Library of Bioconjugates

Conjugate Name	Protein	Modification
BC01	E60C	L1
BC02	E60C	L1-RNA
BC03	E60C	L2
BC04	E60C	L2-RNA
BC05	I61C	L1
BC06	I61C	L1-RNA
BC07	I61C	L2
BC08	I61C	L2-RNA
BC09	E62C	L1
BC10	E62C	L1-RNA
BC11	E62C	L2
BC12	E62C	L2-RNA
BC13	G65C	L1
BC14	G65C	L1-RNA
BC15	G65C	L2
BC16	G65C	L2-RNA
BC17	BH-E62C	L1
BC18	BH-E62C	L1-RNA

1.8 Screening of conjugate library with RNase A

To a white-bottomed 96 well plate (Corning, CLS3605), protein A was added (45 µL of 20 nM, 0.9 pmol) to a final concentration of 9 nM. Protein B was added to a final concentration of 9 nM (45 µL of 20 nM, 0.9 pmol). To this, the LuxSit Pro substrate (Fisher Scientific, Cat no. NC3136279) was added to a final concentration of 50 µM (10 µL of 500 µM, 5 nmol). In the case of conjugate **BC18** (BH-E62C-L1-RNA), the substrate was added to a final concentration of 67 µM (10 µL of 667 µM, 6.7 nmol). Each condition was measured in triplicate (n = 3) except for **BC08** which was measured in duplicate (n = 2). The plate was immediately analyzed with appropriate controls in the plate reader for 30 minutes with 2-minute increments, emission wavelength 300–850 nm. Following 30 minutes, 2 µL of RNase A (20 µg) (Thermo Scientific) were manually added to the bioconjugates, and the samples were immediately reanalyzed in the plate reader for an additional hour. Data was processed using GraphPad Prism (ver. 10.4.1). **Final assay conditions:** 9 nM A, 9 nM B, 20 ug RNase A, 50 or 67 µM substrate, 1% DMF, LuxSit Pro Assay Buffer (Fisher Scientific, Cat no. NC3136279).

1.9 Viral transcript library construction

DNA templates were designed by attaching the T7 RNA polymerase promoter sequence along with two Gs to improve transcription efficacy[2] to the 5' end of gene sequences taken from GenBank for the nucleocapsid protein gene: Respiratory Syncytial Virus (RSV) strain RSV2177 (accession code: AB245475.1); human coronavirus (hCoV) 229E (accession code: NC_002645.1); COVID-19 isolate from Wuhan-Hu-1 (accession code: NC_045512.2), Influenza A H3N2 from 2015 in Washington, USA (accession code: KT844387.1); and Influenza A H1N1 from 2009 in New York, USA (accession code: GQ338407.1).[3] All sequences were ordered as DNA genes from Twist Biosciences without adaptors.

DNA templates were transcribed into RNA using the HiScribe T7 High Yield RNA Synthesis Kit (New England Biolabs). Briefly, 1000 ng of template was incubated with 10 mM ATP, 10 mM GTP, 10 mM UTP, 10 mM CTP, 5 mM DTT, 1X reaction buffer, and the recommended amount of T7 RNA Polymerase mix for 2 hours at 37 °C in a thermocycler. The reactions were then purified with MEGAclear™ Transcription Clean-Up Kit (Invitrogen) according to the manufacturer's protocol. The purity of the transcripts was assessed by denaturing RNA PAGE.

1.10 crRNA design

The designed DNA template for Influenza A H1N1 from 2009 in New York, USA (Genbank accession code: GQ338407.1) was input into the Sanjana lab software.[4,5] The three guide RNAs with the highest guide scores within the fourth quartile were selected. Sequences were further cross-checked in CHOPCHOP v3, an alternative Cas13 software.[6] These spacer sequences were combined with the DR loop sequence for lwCas13a.[7] All sequences were ordered as CRISPR custom guide RNAs from Integrated DNA Technologies (IDT).

1.11 conjRNA2 preparation

In a total volume of 50 µL, 20 µM **conjRNA1** (Sigma) and 200 µM 6-FAM-alkyne (Jena Bioscience) were reacted in the presence of 5 mM sodium ascorbate, 1 mM $CuSO_4$, 3 mM THPTA, and 20 mM HEPES for 2 hours at 37 °C while shaking. The reaction was quenched by the addition of 5 µL of 100 mM EDTA. The RNA was precipitated overnight, and the dry pellet was reconstituted in 50 µL of nuclease free water. The concentration of **conjRNA2** was confirmed by A260, and the purity was confirmed via analytical RP-HPLC and denaturing PAGE.

1.12 Cas13 crRNA efficacy screen

In a total volume of 20 µL, 100 nM of Cas13 (Genscript, Z03486) was treated with 50 nM crRNA in 1X (final from 10X stock) Cas13a reaction buffer (Genscript, Z03486) for 10 minutes at room temperature. To this, 20 ng of **conjRNA2** were added. To initiate degradation, 200 ng of target RNA was added, and the samples were incubated for 30 minutes at room temperature. The reaction was quenched by the addition of 1X (final from 2X stock) RNA loading dye. The samples were boiled at 80 °C for 10 minutes prior to gel loading. The gels were run for 70 minutes at 180 V and were imaged first for fluorescein pre-stained. They were then stained with 1X (final from 10,000X stock) SYBR Gold (Invitrogen) for 10–15 minutes and imaged again for SYBR Gold. Degradation of the fluorescent RNA was quantified in ImageJ, averaged (n = 3), and plotted in GraphPad Prism (ver. 10.4.1) where an ordinary one-way ANOVA was performed with Dunnett's multiple comparisons test.

1.13 Cas13 time trial

In a total volume of 20 µL, 100 nM of Cas13 was treated with 50 nM **crRNA1** in 1X (final from 10X stock) Cas13a reaction buffer for 10 minutes at room temperature. To this, 20 ng of **conjRNA2** was added. To initiate degradation, 200 ng of target RNA was added. To quench the reaction, 1X RNA loading dye (final from 2X stock) was added and the sample was placed on ice at the following time points: 0 mins, 1 min, 2 mins, 3 mins, 4 mins, 5 mins, 10 mins. The samples were boiled at 80 °C for 10 minutes prior to gel loading. The gels were run for 70 minutes at 180 V and were imaged first for fluorescein pre-stained. They were then stained with 1X (final from 10,000X stock) SYBR Gold (Invitrogen) for 10–15 minutes and imaged again for SYBR Gold. Degradation of the fluorescent RNA was quantified in ImageJ, averaged (n = 3), and plotted in GraphPad Prism (ver. 10.4.1).

1.14 Cas13 degradation of BC10 by SDS-PAGE

200 nM Cas13 was pre-incubated with 100 nM **crRNA1** in 1X Cas13a reaction buffer for 5 minutes at room temperature. To this, 0.5 µg of **BC10** (16 µL of 1.8 µM) was added. 400 ng of target RNA was added to activate Cas13, and the sample was incubated for 2 hours at room temperature. The reaction was quenched by the addition of 1X loading dye (from 4X stock) and 1X reducing agent (from 10X stock). The samples were boiled for 5 minutes at 80 °C and loaded onto a protein gel. The gel ran for 35 mins at 200 V. After staining with InstantBlue, the degradation of the conjugate was visualized.

1.15 Sensor matrix screen

To a white-bottomed 96 well plate (Corning, CLS3605), 45 µL of protein A was added at various concentrations (final assay concentration = 20, 10, 5, 2.5, 1, or 0.5 nM). 45 µL of

protein B was added at various concentrations (final assay concentration = 20, 10, 5, 2.5, 1, 0.75, 0.5, or 0.3 nM) such that each well contained a different combination of A and B concentrations. To each well, the LuxSit Pro Substrate was added to a final concentration of 50 µM (10 µL of 500 µM, 5 nmol). In the case of conjugate **BC18** (BH-E62C-L1-RNA), the substrate was added to a final concentration of 67 µM (10 µL of 667 µM, 6.7 nmol). The plate was immediately analyzed in the plate reader for 30 minutes with 1-minute increments, emission wavelength 300–850 nm. Following 30 minutes, 2 µg (**BC18**) or 4 µg (**BC10**) of RNase A were manually added, and the samples were immediately reanalyzed in the plate reader for an additional hour. Data was processed using GraphPad Prism (ver. 10.4.1). The wells with the largest signal change before vs after RNase addition and with the largest overall signal were selected for further use. **Final assay conditions:** 20-0.5 nM A, 20-0.3 nM B, 2 or 4 µg RNase A, 50 or 67 µM substrate, 1% DMF, 1X HBS-EP+ buffer (**BC10**) or LuxSit Pro Assay Buffer (**BC18**), room temperature.

1.16 Sensor limit of detection (LOD) for target RNA

500 nM Cas13 (14 µL of 28.6 µM) was pre-incubated with 250 nM **crRNA1** (2 µL of 100 uM) in 1X Cas13a reaction buffer (80 µL of 10X) and water (704 µL) for 10 minutes at room temperature. To a white-bottomed 96 well plate (Corning, CLS3605), 20 µL of the Cas13 mix was added per well (final 100 nM Cas13, 50 nM **crRNA1**). 35 µL of **BC10** and 35 µL of B protein were added to final assay concentrations of 2.5 nM A + 2.5 nM B, 10 nM A + 5 nM B, or 10 nM A + 0.75 nM B. For the 10 nM A + 5 nM B and 10 nM A + 0.75 nM B conditions, the concentration of Cas13 and **crRNA1** were prepared at 100 nM and 50 nM respectively. To this, the LuxSit Pro Substrate was added to a final concentration of 50 µM (10 µL of 500 uM, 5 nmol). The plate was left in the dark for 25 minutes. Then, the plate was analyzed for 5 minutes to acquire baseline data before 4 µL of **short target RNA** at various concentrations or water were added (200, 100, 50, 25, 12.5, 6.25, 3.13, 1.56, 0.78, 0.39, 0.20, or 0 ng). Each condition was measured in triplicate (n = 3). The plate was then analyzed for an additional hour. The plate was analyzed in 1-minute increments, emission wavelength 300–850 nm, and the data was processed using GraphPad Prism (ver. 10.4.1). **Final assay conditions:** 2.5 nM A + 2.5 nM B or 10 nM A + 5 nM B or 10 nM A + 0.75 nM B, 100 (2.5A+2.5B) or 20 nM Cas13 (10A+5B and 20A+0.75B), 50 (2.5A+2.5B) or 10 nM **crRNA1** (10A+5B and 20A+0.75B), 0–200 ng short target, 50 µM substrate, 1% DMF, 1X HBS-EP+ buffer, room temperature. To determine the LOD, the following equation was used:

$$LOD = 3.3 \times \frac{S_y}{S}$$

Where S_y is the standard is the standard deviation of the y-intercept and S is the slope. For each data set analyzed, seven data points were plotted to generate the regression of the linear range.

1.17 Sensor selectivity for target RNA

100 nM Cas13 (1.4 µL of 28.6 µM) was pre-incubated with 50 nM **crRNA1** (2 µL of 10 µM) in 1X Cas13a reaction buffer (40 µL of 10X) and water (357 µL) for 10 minutes at room temperature. To a white-bottomed 96 well plate (Corning, CLS3605), 20 µL of the Cas13 mix was added per well (final 20 nM Cas13, 10 nM **crRNA1**). **BC10** was added (35 µL of 7.14 nM, 0.25 pmol) to a final concentration of 2.5 nM. Protein B was added to a final concentration of 2.5 nM (35 µL of 7.14 nM, 0.25 pmol). To this, the LuxSit Pro Substrate was added to a final concentration of 50 µM (10 µL of 500 uM, 5 nmol). The plate was left in the dark for 25 minutes. Then, the plate was analyzed for 5 minutes to acquire baseline data before 4 µL of 50 ng/µL target RNA, non-target RNA or water were added. Each condition was measured in triplicate (n = 3). The plate was then analyzed for an additional hour. The plate was analyzed in 1-minute increments, emission wavelength 300–850 nm, and the data was processed using GraphPad Prism (ver. 10.4.1) where an ordinary one-way ANOVA was performed with Dunnett's multiple comparisons test. **Final assay conditions:** 2.5 nM A, 2.5 nM B, 20 nM Cas13, 10 nM **crRNA1**, 200 ng target or non-target RNA, 50 µM substrate, 1% DMF, 1X HBS-EP+ buffer, room temperature.

1.18 Sensor efficacy in serum

100 nM Cas13 (4.5 µL of 28.6 µM) was pre-incubated with 50 nM **crRNA1** (3.15 µL of 10 µM) in 1X Cas13a reaction buffer (90 µL of 10X) and water (802 µL) for 10 minutes at room temperature. To a white-bottomed 96 well plate (Corning, CLS3605), 30 µL of various concentrations of serum (Sigma, H4522) were added (100, 33.3, 16.7, 6.7, 3.3, 1.7, or 0% in 1X HBS-EP+ buffer for a final assay concentration of 30, 10, 5, 2, 1, 0.5, or 0%). Then, 2.5 µL RNase inhibitor was added to each well. 20 µL of the Cas13 mix was added per well (final 20 nM Cas13, 10 nM **crRNA1**). **BC10** was added (20 µL of 12.5 nM, 0.25 pmol) to a final concentration of 2.5 nM. Protein B was added to a final concentration of 2.5 nM (20 µL of 12.5 nM, 0.25 pmol). To this, the LuxSit Pro Substrate was added to a final concentration of 50 µM (7.5 µL of 666 µM, 5 nmol). The plate was left in the dark for 25 minutes. Then, the plate was analyzed for 5 minutes to acquire baseline data before 4 µL of 50 ng/µL target RNA or water were added. Each condition was measured in triplicate (n = 3). The plate was then analyzed for an additional hour. The plate was analyzed in 1-minute increments, emission wavelength 300–850 nm, and the data was processed using GraphPad Prism (ver. 10.4.1) where an ordinary one-way ANOVA was performed with Dunnett's multiple comparisons test. **Final assay conditions:** 2.5 nM A, 2.5 nM B, 20 nM

Cas13, 10 nM **crRNA1**, 200 ng target, 0–30% serum, 1 U/μL RNase inhibitor, 50 μM substrate, 1% DMF, 1X HBS-EP+ buffer, room temperature.

1.19 Long-term luminescence of BC10

100 nM Cas13 (1.58 μL of 28.6 μM) was pre-incubated with 50 nM **crRNA1** (2.25 μL of 10 μM) in 1X Cas13a reaction buffer (45 μL of 10X) and water (401 μL) for 10 minutes at room temperature. To a white-bottomed 96 well plate (Corning, CLS3605), 27.5 μL HBS-EP+ buffer was added followed by 2.5 μL of RNase inhibitor (NEB, M0314L). Then, 20 μL of the Cas13 mix was added per well (final 20 nM Cas13, 10 nM **crRNA1**). **BC10** was added (20 μL of 12.5 nM, 0.25 pmol) to a final concentration of 2.5 nM. Protein B was added to a final concentration of 2.5 nM (20 μL of 12.5 nM, 0.25 pmol). To this, the LuxSit Pro Substrate was added to a final concentration of 50 μM (10 μL of 500 μM, 5 nmol). The plate was left in the dark for 25 minutes. Then, the plate was analyzed for 5 minutes to acquire baseline data before 4 μL of 50 ng/μL target RNA (200 ng) or water were added. Each condition was measured in triplicate (n = 3). The plate was then analyzed for a further 4.5 hours. The plate was analyzed in 2-minute increments, emission wavelength 300–850 nm, and the data was processed using GraphPad Prism (ver. 10.4.1). **Final assay conditions:** 2.5 nM A, 2.5 nM B, 20 nM Cas13, 10 nM **crRNA1**, 50 μM substrate, 1% DMF, 1 X HBS-EP+ buffer, room temperature.

1.20 Substrate solvent preference for sensor

The LuxSit Pro Substrate was dissolved in DMSO or DMF to a stock concentration of 5 mM. To a white-bottomed 96 well plate (Corning, CLS3605), **BC10** was added (45 μL of 20 nM, 0.9 pmol) to a final concentration of 9 nM. Protein B was added to a final concentration of 9 nM (45 μL of 20 nM, 0.9 pmol). To this, the substrate dissolved in DMF or DMSO was added to a final concentration of 50 μM (10 μL of 500 μM, 5 nmol). Each condition was measured in duplicate (n = 2). The plate was immediately analyzed in the plate reader for 10 minutes with 2-minute increments, emission wavelength 300–850 nm. 2 μL of RNase A were manually added (20 μg) and the samples were immediately reanalyzed in the plate reader for an additional hour. Data was processed using GraphPad Prism (ver. 10.4.1). **Final assay conditions:** 9 nM A, 9 nM B, 20 μg RNase A, 50 μM substrate, 1% DMF or DMSO, LuxSit Pro Assay buffer, room temperature.

1.21 Effect of RNase inhibitor on sensor signal

100 nM Cas13 (2.8 μL of 28.6 μM) was pre-incubated with 50 nM **crRNA1** (5 μL of 10 μM) in 1X Cas13a reaction buffer (100 μL of 10X) and water (892 μL) for 10 minutes at room temperature. To a white-bottomed 96 well plate (Corning, CLS3605), 27.5 μL of HBS-EP+ buffer was added. Then, 2.5 μL RNase inhibitor (or water) was added. 20 μL of the Cas13

mix was added per well (final 20 nM Cas13, 10 nM **crRNA1**). **BC10** was added (20 µL of 12.5 nM, 0.25 pmol) to a final concentration of 2.5 nM. Protein B was added to a final concentration of 2.5 nM (20 µL of 12.5 nM, 0.25 pmol). To this, the LuxSit Pro Substrate was added to a final concentration of 50 µM (10 µL of 500 µM, 5 nmol). The plate was left in the dark for 25 minutes. Then, the plate was analyzed for 5 minutes to acquire baseline data before 4 µL of 50 ng/µL target or water were added. Each condition was measured in triplicate (n = 3). The plate was then analyzed for an additional hour. The plate was analyzed in 1-minute increments, emission wavelength 300–850 nm, and the data was processed using GraphPad Prism (ver. 10.4.1). **Final assay conditions:** 2.5 nM A, 2.5 nM B, 20 nM Cas13, 10 nM **crRNA1**, 200 ng target, 1 or 0 U/µL RNase inhibitor, 50 µM substrate, 1% DMF, 1X HBS-EP+ buffer, room temperature.

1.22 Linker Synthesis

1.22.1 1, *tert*-butyl (4-(2,5-dioxo-2,5-dihydro-1*H*-pyrrol-1-yl)butyl)carbamate

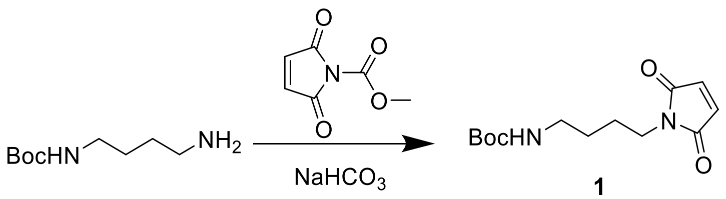

165 mg (1.06 mmol) of N-(methoxycarbonyl)maleimide was added to a 0 °C solution of 200 mg (1.06 mmol) *tert*-butyl (4-aminobutyl)carbamate saturated with $NaHCO_3$ (4 mL). 2 mL of dichloromethane (DCM) were added to aid solubility, and the solution was stirred at 0 °C for 40 minutes. The reaction was brought to room temperature and stirred for an additional 50 minutes. The reaction pH was brought to 5 with the dropwise addition of HCl, and the solution was extracted with ethyl acetate (3 x 5 mL). The organic layer was dried with anhydrous $MgSO_4$, and the solution was concentrated *in vacuo*. The sample was then purified via Preparative Thin Layer Chromatography (Prep-TLC) with a 20:1 DCM:Methanol (MeOH) mobile phase. 51.6% yield was achieved.

LC-MS m/z $[M+Na]^+$ calculated = 291.30. Found = 291.2.

^{1}H NMR (700 MHz, $CDCl_3$) δ 6.68 (s, 2H), 4.53 (br s, 1H), 3.53 (t, *J* = 7.2 Hz, 2H), 3.13 (m, *J* = 6.8 Hz, 2H), 1.64 – 1.57 (m, 2H), 1.47 (m, 2H), 1.43 (s, 9H). **^{13}C NMR** (176 MHz, $CDCl_3$) δ 170.76, 155.87, 134.05, 79.16, 39.95, 37.42, 28.37, 27.34, 25.87.

1.22.2 2, *tert*-butyl (2-(2-(2-(2,5-dioxo-2,5-dihydro-1*H*-pyrrol-1-yl)ethoxy)ethoxy)ethyl)carbamate

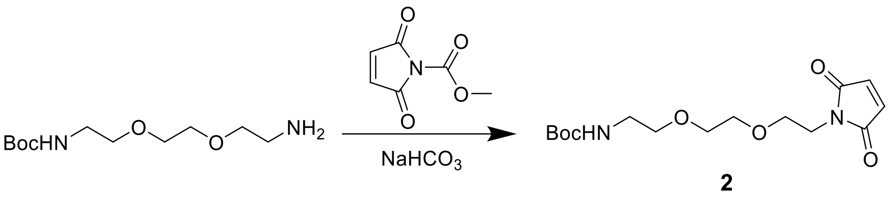

To a 0 °C solution of 250 mg (1.01 mmol) *tert*-butyl (2-(2-(2-aminoethoxy)ethoxy)ethyl)carbamate saturated with $NaHCO_3$ (4 mL), 156 mg (1.01 mmol) of N-(methoxycarbonyl)maleimide was added. 2 mL of DCM were added to aid solubility, and the solution was stirred at 0 °C for 40 minutes. The reaction was brought to room temperature and stirred for an additional 50 minutes. The reaction pH was brought to 5 with the dropwise addition of HCl, and the solution was extracted with ethyl acetate (3 x 5 mL). The organic layer was dried with anhydrous $MgSO_4$, and the solution was concentrated *in vacuo*. The sample was then purified via Prep-TLC with 20:1 DCM:MeOH mobile phase. 36.3% yield was achieved.

LC-MS m/z $[M+Na]^+$ calculated = 351.15. Found = 351.3.

^{1}**H NMR** (400 MHz, $CDCl_3$) δ 6.71 (s, 2H), 5.03 (br s, 1H), 3.73 (t, *J* = 6.1 Hz, 2H), 3.69 – 3.62 (m, 2H), 3.61 – 3.52 (m, 4H), 3.49 (t, *J* = 5.1 Hz, 2H), 3.29 (m, 2H), 1.44 (s, 9H). ^{13}**C NMR** (101 MHz, $CDCl_3$) δ 170.81, 156.15, 134.31, 79.33, 70.42, 70.06, 67.97, 52.44, 40.54, 37.19, 28.57.

1.22.3 3, 1-(4-aminobutyl)-1*H*-pyrrole-2,5-dione

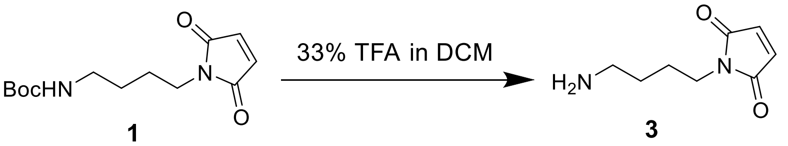

Approximately 147 mg (548 μmol) of **1** was dissolved in 2 mL of dry DCM and 1 mL TFA. The solution was stirred for 2 hours at room temperature. Yield was quantitative.

LC-MS m/z $[M+H]^+$ calculated = 169.20. Found = 169.0.

^{1}**H NMR** (400 MHz, DMSO) δ 7.71 (br s, 3H), 7.04 (s, 2H), 3.41 (t, *J* = 6.4 Hz, 2H), 2.77 (m, *J* = 6.0 Hz, 2H), 1.69 – 1.37 (m, 4H). ^{13}**C NMR** (101 MHz, DMSO) δ 170.50, 133.92, 37.77, 35.89, 24.43, 23.75.

1.22.4 4, 1-(2-(2-(2-aminoethoxy)ethoxy)ethyl)-1*H*-pyrrole-2,5-dione

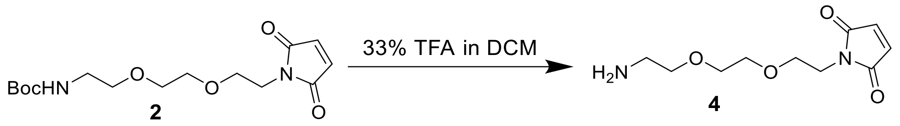

Approximately 120 mg (365 µmol) of **2** was dissolved in 2 mL of dry DCM and 1 mL TFA. The solution was stirred for 2 hours at room temperature. Yield was quantitative.

LC-MS m/z $[M+H]^+$ calculated = 229.11. Found = 229.1.

^{1}H NMR (400 MHz, DMSO) δ 7.82 (br s, 3H), 7.03 (s, 2H), 3.84 – 3.27 (m, 10H), 2.96 (m, *J* = 5.6 Hz, 2H). **^{13}C NMR** (101 MHz, DMSO) δ 170.95, 134.60, 69.60, 69.21, 67.00, 66.68, 38.59, 36.76.

1.22.5 5, Linker 1 (L1)

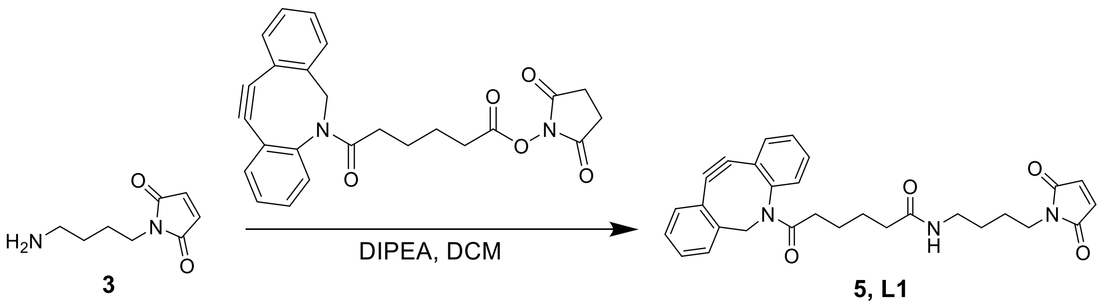

A solution containing DBCO-NHS ester (Lumiprobe) (25 mg, 58 µmol) and **3** (16 mg, 57 µmol) in dry DCM (2 mL) was treated with N-ethyl-N-isopropylpropan-2-amine (DIPEA) (20 µL, 0.12 mmol) and was stirred for one hour at room temperature. The reaction was concentrated *in vacuo* and purified via Prep-TLC with a 20:1 DCM:MeOH mobile phase. 79% yield was achieved.

LC-MS m/z $[M+H]^+$ calculated = 484.57. Found = 484.4.

^{1}H NMR (400 MHz, $CDCl_3$) δ 7.70 (d, *J* = 7.5 Hz, 1H), 7.45 – 7.26 (m, 6H), 6.69 (s, 2H), 5.69 (br s, 1H), 5.15 (d, *J* = 13.8 Hz, 1H), 3.67 (d, *J* = 13.8 Hz, 1H), 3.52 (t, *J* = 7.1 Hz, 2H), 3.16 (m, 2H), 2.25 (dt, *J* = 15.7, 6.3 Hz, 1H), 2.00 – 1.81 (m, 3H), 1.43 (h, *J* = 6.8 Hz, 5H), 1.31 – 1.22 (m, 3H), 0.83 (s, 1H).

1.22.6 6, Linker 2 (L2)

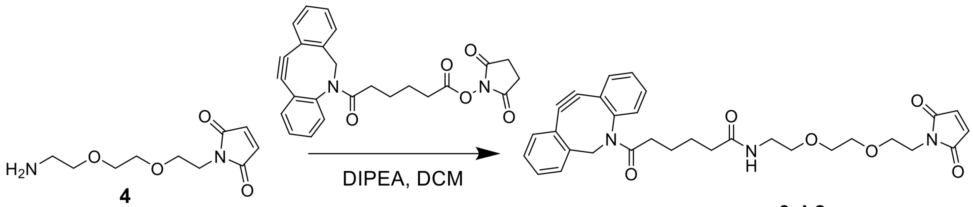

A solution containing DBCO-NHS ester (Lumiprobe) (25 mg, 58 μmol) and **4** (20 mg, 58 μmol) in dry DCM (2 mL) was treated with DIPEA (20 μL, 0.11 mmol) and was stirred for one hour at room temperature. The reaction was concentrated *in vacuo* and purified via Prep-TLC with a 20:1 DCM:MeOH mobile phase. 94% yield was achieved.

LC-MS m/z $[M+H]^+$ calculated = 544.62. Found = 544.5.

^{1}**H NMR** (400 MHz, CDCl3) δ 7.69 (d, *J* = 7.5 Hz, 1H), 7.44 – 7.21 (m, 7H), 6.68 (s, 2H), 6.03 (br s, 1H), 5.14 (d, *J* = 13.8 Hz, 1H), 3.75 – 3.47 (m, 11H), 3.45 (t, *J* = 5.1 Hz, 2H), 3.42 – 3.28 (m, 2H), 2.23 (dt, *J* = 15.1, 6.6 Hz, 1H), 1.99 (t, *J* = 6.7 Hz, 2H), 1.88 (dt, *J* = 15.2, 6.8 Hz, 1H), 1.43 (dtd, *J* = 10.5, 6.4, 3.4 Hz, 2H). ^{13}**C NMR** (101 MHz, $CDCl_3$) δ 173.31, 172.95, 170.85, 148.26, 134.33, 132.48, 129.13, 128.54, 128.40, 128.17, 127.85, 127.26, 125.59, 123.25, 122.68, 77.48, 77.36, 77.16, 76.84, 70.35, 70.04, 68.02, 55.46, 39.19, 37.30, 36.25, 34.63, 29.85, 25.15, 24.90.

2. Protein, DNA, and RNA sequences

2.1 Protein Sequences

2.1.1 E60C

MSGGRQEKVLKSIEETVRKMGVTMETHRSGNEVKVVIKGLHIKQQRQLYRDVRETSKKQGVETCIE
VEGDTVTIVVREGGGSGSGGGSGSGGGNRVDEVRVYINPTGSWGSHHHHHH

2.1.2 I61C

MSGGRQEKVLKSIEETVRKMGVTMETHRSGNEVKVVIKGLHIKQQRQLYRDVRETSKKQGVETECE
VEGDTVTIVVREGGGSGSGGGSGSGGGNRVDEVRVYINPTGSWGSHHHHHH

2.1.3 E62C

MSGGRQEKVLKSIEETVRKMGVTMETHRSGNEVKVVIKGLHIKQQRQLYRDVRETSKKQGVETEIC
VEGDTVTIVVREGGGSGSGGGSGSGGGNRVDEVRVYINPTGSWGSHHHHHH

2.1.4 G65C

MSGGRQEKVLKSIEETVRKMGVTMETHRSGNEVKVVIKGLHIKQQRQLYRDVRETSKKQGVETEIE
VECDTVTIVVREGGGSGSGGGSGSGGGNRVDEVRVYINPTGSWGSHHHHHH

2.1.5 AWT

MSGGRQEKVLKSIEETVRKMGVTMETHRSGNEVKVVIKGLHIKQQRQLYRDVRETSKKQGVETEIE
VEGDTVTIVVREGGGSGSGGGSGSGGGNRVDEVRVYINPTGSWGSHHHHHH

2.1.6 BWT

MSGGRQEKVLKSIEETVRKMGVTMETHRSGNEVKVVIKGLHESQQEQLLEDVLRTAEKQGVRVRIR
FKGDTVTIVVREGGGSGSGGGSGSGGGSEEQIRQNLRRFYEALDSGDADTAASLFHPGVTIHLWD
GVTFTSREEFREWFERLFSTSKDALREIKSLEVRGDTVEVHVQLHFTRNGQKHTVDLTHLFHFRGS
WGSHHHHHH

2.1.7 BH-E62C

MKETAAAKFERQHMDSPDLGTLVPRGSMSGGRQEKVLKSIEETVRKMGVTMETHRSGNEVKVVIK
GLHIKQQRQLYRDVRETSKKQGVETEICVEGDTVTIVVREGGGSGSGGGSGSGGGRVVAVRVYVN
PTGSWGSHHHHHH

2.1.8 BH-AWT

MKETAAAKFERQHMDSPDLGTLVPRGSMSGGRQEKVLKSIEETVRKMGVTMETHRSGNEVKVVIK
GLHIKQQRQLYRDVRETSKKQGVETEIEVEGDTVTIVVREGGGSGSGGGSGSGGGRVVAVRVYVN
PTGSWGSHHHHHH

2.1.9 BH-BWT

MKETAAAKFERQHMDSPDLGTLVPRGSMSGGRQEKVLKSIEETVRKMGVTMETHRSGNEVKVVIK
GLHESQQEQLLEDVLRTAEKQGVRVRIRFKGDTVTIVVREGGGSGSGGGSGSGGGTPEELRQNLL
RFYEALDSGDAVAAASLFDPGVTITLWDGTTFTSNEEFREWFETQFRTSRDAFREITSLEVDGDTVRV
TVRLTFTRDGEEQVVDLLQLFEFAGSWGSHHHHHH

2.1.10 Extinction coefficients, molecular weights, and isoelectric points (pI)

Table S2: Library of proteins

Protein	Extinction coefficient ($M^{-1}cm^{-1}$)	Molecular Weight (Da)	pI
**E60C**	8480	12,596.02	9.20
**I61C**	8480	12,611.97	8.82
**E62C**	8480	12,596.02	9.20
**G65C**	8480	12,668.08	8.82
**AWT**	8480	12,621.99	9.05
**BWT**	17990	23,057.63	6.95
**BH-E62C**	8480	15,480.44	9.56
**BH-AWT**	8480	15,506.42	9.52
**BH-BWT**	17990	25,847.74	5.52

2.2 Viral Sequences:

2.2.1 H1N1 NP template (Target RNA)

<u>TAATACGACTCACTATAGGG</u>ATGGCGTCTCAAGGCACCAAACGATCATATGAACAAATGGAGAC
TGGTGGGGAGCGCCAGGATGCCACAGAAATCAGAGCATCTGTCGGAAGAATGATTGGTGGAAT
CGGGAGATTCTACATCCAAATGTGCACTGAACTCAAACTCAGTGATTATGATGGACGACTAATCC
AGAATAGCATAACAATAGAGAGGATGGTGCTTTCTGCTTTTGATGAGAGAAGAAATAAATACCTAG
AAGAGCATCCCAGTGCTGGGAAGGACCCTAAGAAAACAGGAGGACCCATATATAGAAGAATAG
ACGGAAAGTGGATGAGAGAACTCATCCTTTATGACAAAGAAGAAATAAGGAGAGTTTGGCGCCA
AGCAAACAATGGCGAAGATGCAACAGCAGGTCTTACTCATATCATGATTTGGCATTCCAACCTGA
ATGATGCCACATATCAGAGAACAAGAGCGCTTGTTCGCACCGGAATGGATCCCAGAATGTGCTC
TCTAATGCAAGGTTCAACACTTCCCAGAAGGTCTGGTGCCGCAGGTGCTGCGGTGAAAGGAGT
TGGAACAATAGCAATGGAGTTAATCAGAATGATCAAACGTGGAATCAATGACCGAAATTTCTGGA
GGGGTGAAAATGGACGAAGGACAAGGGTTGCTTATGAAAGAATGTGCAATATCCTCAAAGGAAA
ATTTCAAACAGCTGCCCAGAGGGCAATGATGGATCAAGTAAGAGAAAGTCGAAACCCAGGAAA
CGCTGAGATTGAAGACCTCATTTTCCTGGCACGGTCAGCACTCATTCTGAGGGGATCAGTTGCA
CATAAATCCTGCCTGCCTGCTTGTGTGTATGGGCTTGCAGTAGCAAGTGGGCATGACTTTGAAAG
GGAAGGGTACTCACTGGTCGGGATAGACCCATTCAAATTACTCCAAAACAGCCAAGTGGTCAG

CCTGATGAGACCAAATGAAAACCCAGCTCACAAGAGTCAATTGGTGTGGATGGCATGCCACTCT
GCTGCATTTGAAGATTTAAGAGTATCAAGTTTCATAAGAGGAAAGAAAGTGATTCCAAGAGGAAAG
CTTTCCACAAGAGGGGTCCAGATTGCTTCAAATGAGAATGTGGAAACCATGGACTCCAATACCC
TGGAACTAAGAAGCAGATACTGGGCCATAAGGACCAGGAGTGGAGGAAATACCAATCAACAAA
AGGCATCCGCAGGCCAGATCAGTGTGCAGCCTACATTCTCAGTGCAGCGAAATCTCCCTTTTGA
AAGAGCAACCGTTATGGCAGCATTCAGCGGGAACAATGAAGGACGGACATCCGACATGCGAA
CAGAAGTTATAAGAATGATGGAAAGTGCAAAGCCAGAAGATTTGTCCTTCCAGGGGCGGGGAGT
CTTCGAGCTCTCGGACGAAAAGGCAACGAACCCGATCGTGCCTTCCTTTGACATGAGTAATGAA
GGGTCTTATTTCTTCGGAGACAATGCAGAGGAGTATGACAGTTGA

2.2.2 H3N2 NP template

<u>TAATACGACTCACTATAGGG</u>ATGGCGTCCCAAGGCACCAAACGGTCTTATGAACAGATGGAAA
CTGATGGAGATCGCCAGAATGCAACTGAGATTAGGGCATCCGTCGGGAAGATGATTGATGGAAT
TGGGAGATTCTACATCCAAATGTGCACTGAACTTAAACTCAGTGATCATGAAGGACGGTTGATCC
AGAACAGCTTGACAATAGAGAAAATGGTACTCTCTGCTTTTGATGAAAGAAGGAATAAATACCTGG
AAGAACACCCCAGCGCGGGGAAAGATCCCAAGAAAACTGGGGGGCCCATATACAGGAGAGT
CGATGGGAAATGGATGAGGGAACTCGTCCTTTATGACAAAGAAGAAATAAGGCGAATCTGGCGC
CAAGCCAACAATGGTGAGGATGCTACATCTGGTCTAACTCACATAATGATTTGGCATTCCAATTTG
AATGATGCAACATACCAGAGGACAAGAGCTCTTGTCCGAACTGGAATGGATCCCAGAATGTGCT
CTCTGATGCAGGGCTCGACTCTCCCTAGAAGGTCCGGAGCTGCAGGTGCTGCAGTCAAAGGA
ATCGGAACAATGGTGATGGAACTGATCAGAATGGTCAAACGGGGGATCAACGATCGAAATTTTTG
GAGAGGTGAGAATGGGCGGAAAACAAGAAGTGCTTATGAGAGAATGTGCAACATTCTTAAAGGA
AAATTTCAAACAGCTGCACAAAGAGCAATGGTGGATCAAGTTAGAGAAAGTCGGAACCCAGGAA
ACGCTGAGATCGAAGATCTCATATTTTTAGCAAGATCTGCACTGATATTGAGAGGATCAGTTGCTC
ACAAATCTTGCCTACCTGCCTGTGCATATGGACCTGCAGTATCCAGTGGTTACGACTTTGAAAAA
GAGGGATATTCCTTGGTGGGAATAGACCCTTTCAAACTACTTCAAAATAGCCAAATATACAGCTTA
ATCAGACCTAATGAGAATCCAGCACACAAGAGTCAGCTGGTGTGGATGGCATGCCATTCTGCTG
CATTTGAAGATTTAAGATTGTTAAGCTTCATCAGAGGGACAAAAGTATCTCCTCGGGGGAAACTGT
CAACTAGAGGAGTACAAATTGCTTCAAATGAGAACATGGATAATATGGGATCGAGCACTCTTGAA
CTGAGAAGCGGGTACTGGGCCATAAGGACCAGGAGTGGAGGAAACACTAATCAACAGAGGGC
CTCCGCAGGCCAAACCAGTGTGCAACCTACGTTTTCTGTACAAAGAAACCTCCCATTTGAAAAG
TCAACCATCATGGCAGCATTCACTGGAAATACGGAGGGAAGAACTTCAGACATGAGGGCAGAA
ATCATAAGGATGATGGAAGGTGCAAAACCAGAAGAAGTGTCATTCCGGGGGAGGGGAGTTTTC
GAGCTCTCAGACGAGAAGGCAACGAACCCGATCGTGCCCTCTTTTGATATGAGTAACGAAGGAT
CTTATTTCTTCGGAGACAATGCAGAAGAGTACGACAATTAA

2.2.3 COVID NP template

<u>TAATACGACTCACTATAGGG</u>ATGTCTGATAATGGACCCCAAAATCAGCGAAATGCACCCCGCAT
TACGTTTGGTGGACCCTCAGATTCAACTGGCAGTAACCAGAATGGAGAACGCAGTGGGGCGCG

ATCAAAACAACGTCGGCCCCAAGGTTTACCCAATAATACTGCGTCTTGGTTCACCGCTCTCACT
CAACATGGCAAGGAAGACCTTAAATTCCCTCGAGGACAAGGCGTTCCAATTAACACCAATAGCA
GTCCAGATGACCAAATTGGCTACTACCGAAGAGCTACCAGACGAATTCGTGGTGGTGACGGTAA
AATGAAAGATCTCAGTCCAAGATGGTATTTCTACTACCTAGGAACTGGGCCAGAAGCTGGACTTC
CCTATGGTGCTAACAAAGACGGCATCATATGGGTTGCAACTGAGGGAGCCTTGAATACACCAAA
AGATCACATTGGCACCCGCAATCCTGCTAACAATGCTGCAATCGTGCTACAACTTCCTCAAGGA
ACAACATTGCCAAAAGGCTTCTACGCAGAAGGGAGCAGAGGCGGCAGTCAAGCCTCTTCTCG
TTCCTCATCACGTAGTCGCAACAGTTCAAGAAATTCAACTCCAGGCAGCAGTAGGGGAACTTCT
CCTGCTAGAATGGCTGGCAATGGCGGTGATGCTGCTCTTGCTTTGCTGCTGCTTGACAGATTGA
ACCAGCTTGAGAGCAAAATGTCTGGTAAAGGCCAACAACAACAAGGCCAAACTGTCACTAAGA
AATCTGCTGCTGAGGCTTCTAAGAAGCCTCGGCAAAAACGTACTGCCACTAAAGCATACAATGTA
ACACAAGCTTTCGGCAGACGTGGTCCAGAACAAACCCAAGGAAATTTTGGGGACCAGGAACTA
ATCAGACAAGGAACTGATTACAAACATTGGCCGCAAATTGCACAATTTGCCCCCAGCGCTTCAG
CGTTCTTCGGAATGTCGCGCATTGGCATGGAAGTCACACCTTCGGGAACGTGGTTGACCTACAC
AGGTGCCATCAAATTGGATGACAAAGATCCAAATTTCAAAGATCAAGTCATTTTGCTGAATAAGCA
TATTGACGCATACAAAACATTCCCACCAACAGAGCCTAAAAAGGACAAAAAGAAGAAGGCTGAT
GAAACTCAAGCCTTACCGCAGAGACAGAAGAAACAGCAAACTGTGACTCTTCTTCCTGCTGCA
GATTTGGATGATTTCTCCAAACAATTGCAACAATCCATGAGCAGTGCTGACTCAACTCAGGCCTA
A

2.2.4 hCoV-229E NP template

TAATACGACTCACTATAGGGATGGCTACAGTCAAATGGGCTGATGCATCTGAACCACAACGTGG
TCGTCAGGGTAGAATACCTTATTCTCTTTATAGCCCTTTGCTTGTTGATAGTGAACAACCTTGGAAG
GTGATACCTCGTAATTTGGTACCCATCAACAAGAAAGACAAAAATAAGCTTATAGGCTATTGGAAT
GTTCAAAAACGTTTCAGAACTAGAAAGGGCAAACGGGTGGATTTGTCACCCAAGCTGCATTTTTA
TTATCTTGGCACAGGACCCCATAAAGATGCAAAATTTAGAGAGCGTGTTGAAGGTGTCGTCTGGG
TTGCTGTTGATGGTGCTAAAACTGAACCTACAGGTTACGGTGTTAGGCGCAAGAATTCAGAACCA
GAGATACCACACTTCAATCAAAAGCTCCCAAATGGTGTTACTGTTGTTGAAGAACCTGACTCCCG
TGCTCCTTCCCGGTCTCAGTCGAGGTCGCAGAGTCGCGGTCGTGGTGAATCCAAACCTCAATC
TCGGAATCCTTCAAGTGACAGAAACCATAACAGTCAGGATGACATCATGAAGGCAGTTGCTGCG
GCTCTTAAATCTTTAGGTTTTGACAAGCCTCAGGAAAAAGATAAAAAGTCAGCGAAAACGGGTAC
TCCTAAGCCTTCTCGTAATCAGAGTCCTGCTTCTTCTCAAACTTCTGCCAAGAGTCTTGCTCGTTC
TCAGAGTTCTGAAACAAAAGAACAAAAGCATGAAATGCAAAAGCCACGGTGGAAAAGACAGCC
TAATGATGATGTGACATCTAATGTCACACAATGTTTTGGCCCCAGAGACCTTGACCACAACTTTGG
AAGTGCAGGTGTTGTGGCCAATGGTGTTAAAGCTAAAGGCTATCCACAATTTGCTGAGCTTGTGC
CGTCAACAGCTGCTATGCTGTTTGATAGTCACATTGTTTCCAAAGAGTCAGGCAACACTGTGGTCT
TGACTTTCACTACTAGAGTGACTGTGCCCAAAGACCATCCACACTTGGGTAAGTTTCTTGAGGAG
TTAAATGCATTCACTAGAGAAATGCAACAACATCCTCTTCTTAACCCTAGTGCACTAGAATTCAAC

CCATCTCAAACTTCACCTGCAACTGCTGAACCAGTGCGTGATGAAGTTTCTATTGAAACTGACATAATTGATGAAGTAAACTAA

2.2.5 RSV NP template

TAATACGACTCACTATAGGGATGGCTCTTAGCAAAGTCAAGTTAAATGATACATTAAATAAGGATCAGCTGCTGTCATCCAGCAAATACACTATTCAACGTAGTACAGGAGATAATATTGACACTCCCAATTATGATGTGCAAAAACACCTAAACAAACTATGTGGTATGCTATTAATCACTGAAGATGCTAATCATAAATTCACAGGATTAATAGGTATGTTATATGCTATGTCCAGGTTAGGAAGGGAAGACACTATAAAGATACTTAAAGATGCTGGATATCATGTTAAAGCTAATGGAGTAGATATAACAACATATCGTCAAGATATAAATGGTAAGGAAATGAAATTCGAAGTATTAACATTATCAAGCTTGACATCAGAAATACAAGTCAATATTGAGATAGAATCTAGAAAGTCCTACAAAAAAATGCTAAAAGAGATGGGAGAAGTTGCTCCAGAATATAGGCATGATTCTCCAGACTGCGGGATGATAATACTGTGCATAGCTGCTCTTGTAATAACCAAATTAGCAGCAGGAGATAGATCAGGTCTTACAGCAGTAATTAGGAGGGCAAACAATGTCTTAAAAAACGAAATAAAACGCTACAAGGGCCTAATACCAAGGGATATAGCTAACAGTTTTTATGAAGTGTTTGAAAAACACCCTCATCTTATAGATGTTTTTGTGCACTTTGGCATTGCACAATCATCCACAAGAGGGGGTAGTAGAGTTGAAGGAATCTTTGCAGGATTGTTTATGAATGCCTATGGTTCAGGACAAGTAATGCTAAGATGGGGAGTCTTAGCCAAATCTGTAAAAAATATCATGCTAGGACATGCTAGTGTCCAAGCAGAAATGGAGCAAGTTGTGGAAGTCTATGAGTATGCACAGAAGTTGGGAGGAGAAGCTGGATTCTACCATATATTGAATAATCCAAAAGCATCATTGCTGTCATTAACTCAATTCCCTAACTTCTCAAGTGTGGTCCTAGGCAATGCTGCAGGTCTAGGCATAATGGGAGAGTATAGAGGTACACCAAGAAACCAAGATCTTTATGATGCAGCCAAAGCATATGCAGAGCAACTCAAAGAAAATGGAGTAATAAACTACAGTGTATTAGACTTAACAGCAGAAGAATTGGAAGCCATAAAGCATCAACTCAACCCCAAAGAAGATGATGTAGAGCTTTAA

2.2.6 Shortened H1N1 NP RNA (short Target RNA)

GAAAAUGGACGAAGGACAAGGGUUGCUUAUGAAAGAAUGUGCAAUAUCCUCAAAGGAAAA

2.3 crRNA sequences

2.3.1 crRNA1

GAUUUAGACUACCCCAAAAACGAAGGGGACUAAAACCACAUUCUUUCAUAAGCAACCCU

2.3.2 crRNA2

GAUUUAGACUACCCCAAAAACGAAGGGGACUAAAACUUAAAUCUUCAAAUGCAGCAGAG

2.3.3 crRNA3

GAUUUAGACUACCCCAAAAACGAAGGGGACUAAAACAUCAAAAGCAGAAAGCACCAUCC

2.4 Conjugate RNA

2.4.1 conjRNA1

N_3/GAUUUCUUUCUUUGAGG

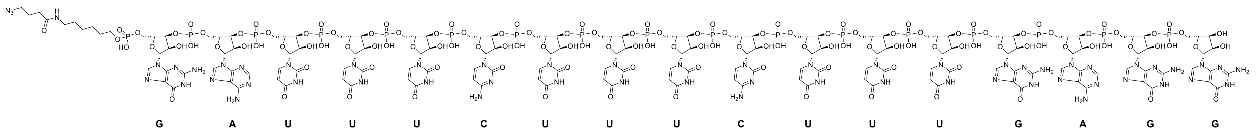

Figure S1: Chemical structure of **conjRNA1**. Molecular weight = 5,635 Da.

2.4.2 conjRNA2

FAM/GAUUUCUUUCUUUGAGG

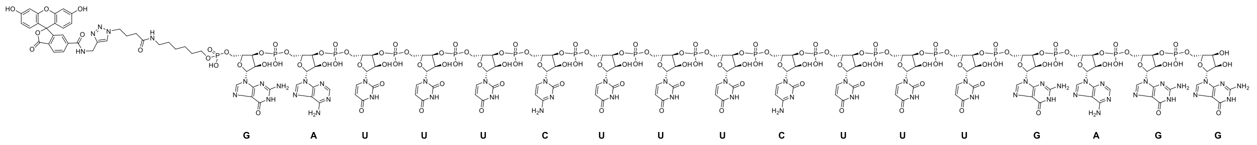

Figure S2: Chemical structure of **conjRNA2**.

3. LC-MS Spectra

3.1 LC-MS Spectra of Proteins

3.1.1 E60C LC-MS

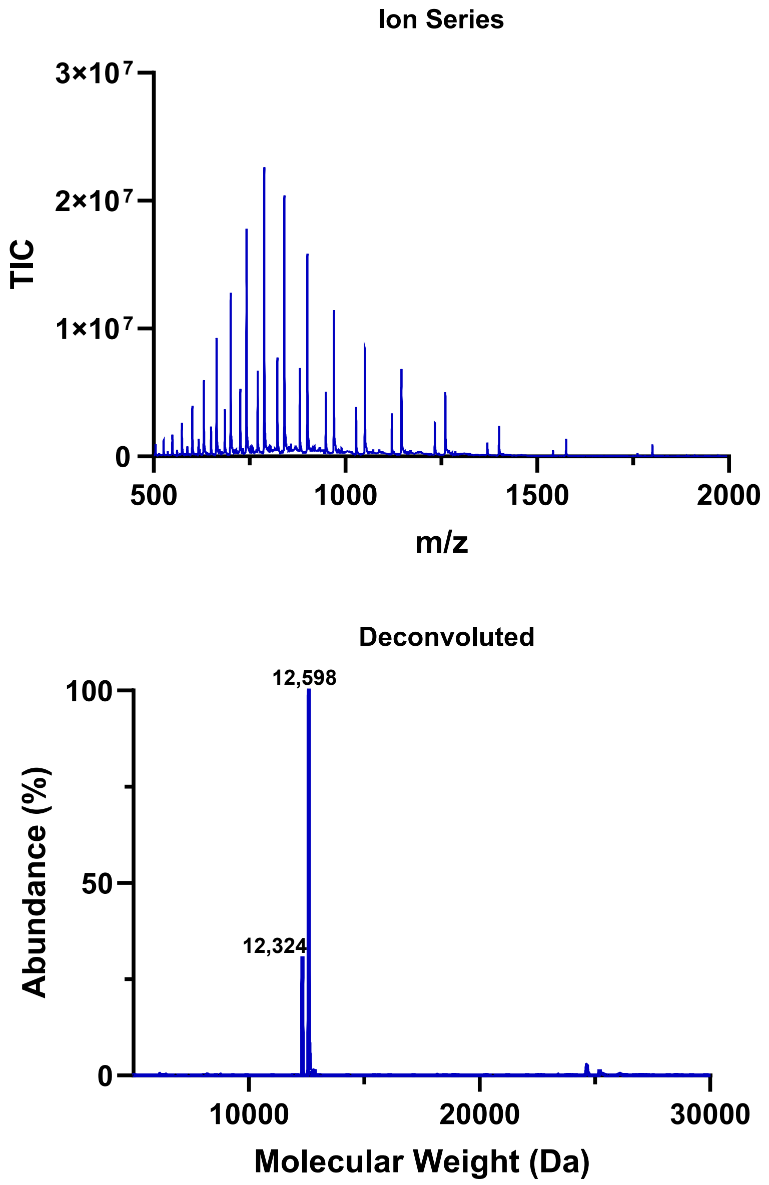

Figure S3: LC–MS spectra of E60C ion series and deconvolution. Calculated molecular weight: 12,596.02 Da. M – 274 Da corresponds to truncation of the C-terminal 6xHis tag by two histidines.

3.1.2 I61C LC-MS

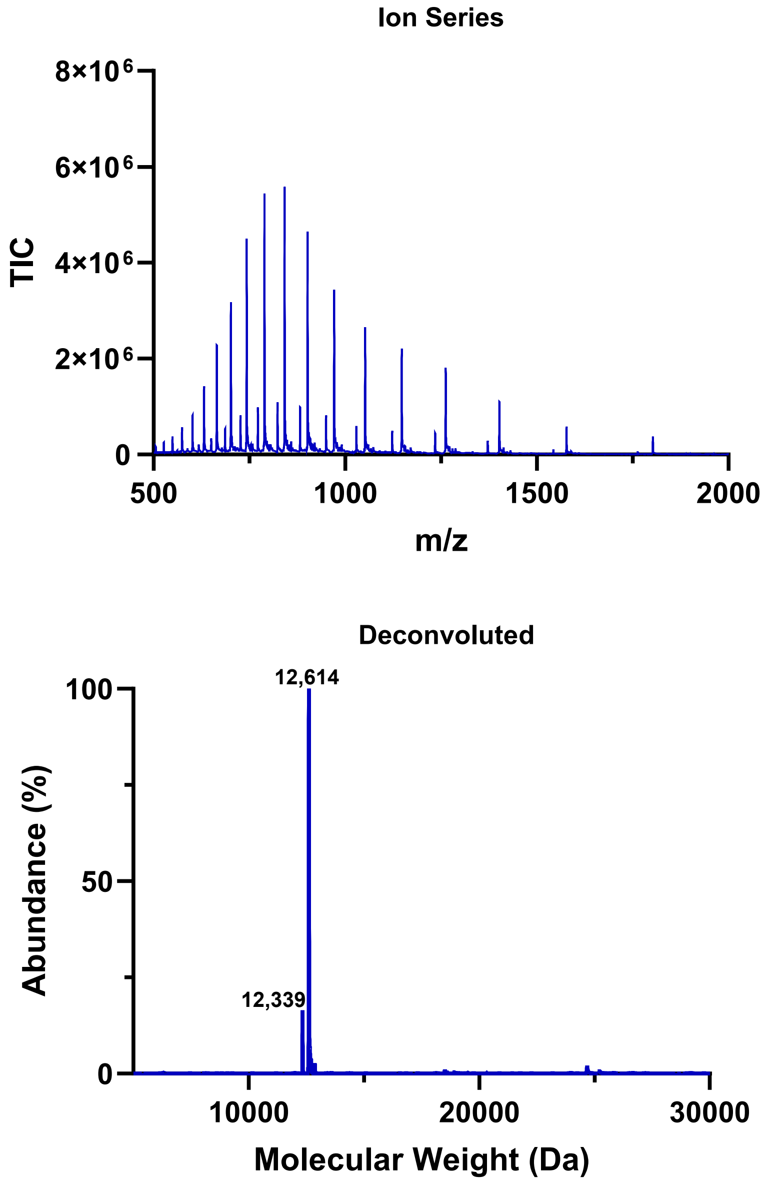

Figure S4: LC–MS spectra of I61C ion series and deconvolution. Calculated molecular weight: 12,611.97 Da. M – 275 Da corresponds to truncation of the C-terminal 6xHis tag by two histidines.

3.1.3 E62C LC-MS

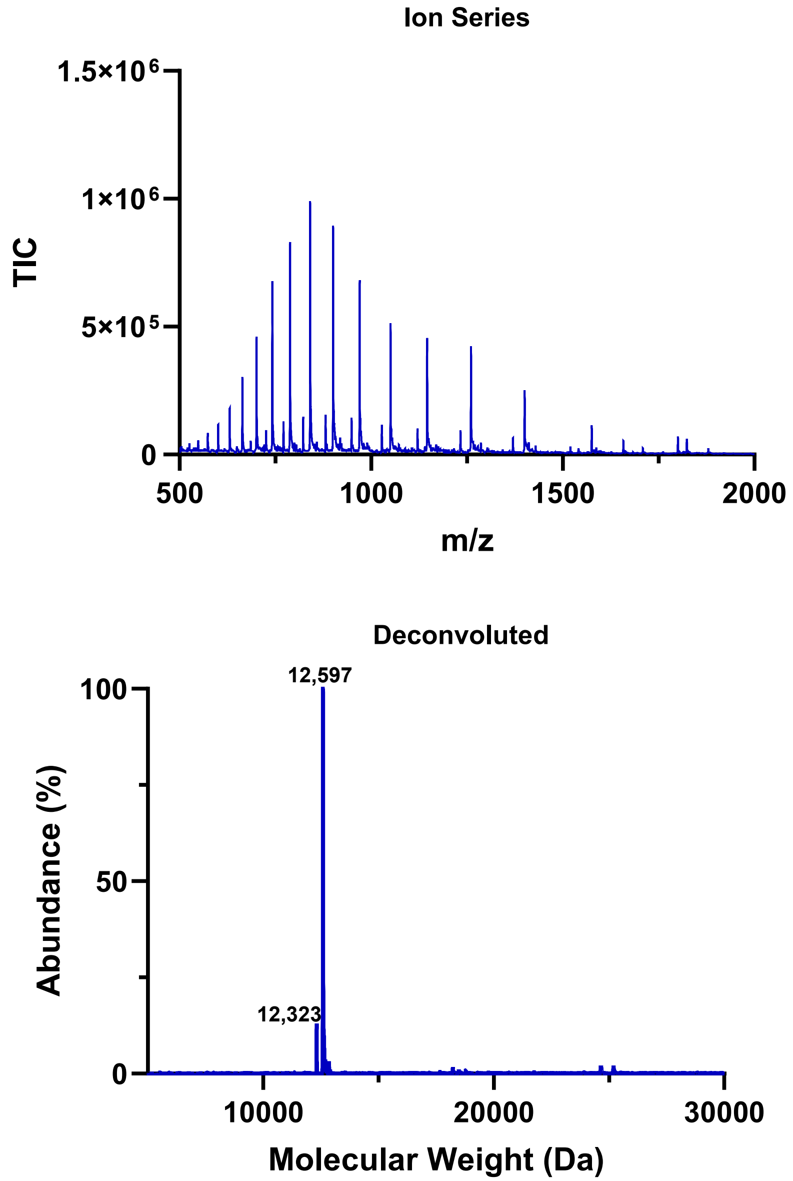

Figure S5: LC–MS spectra of E62C ion series and deconvolution. Calculated molecular weight: 12,596.02 Da. M – 274 Da corresponds to truncation of the C-terminal 6xHis tag by two histidines.

3.1.4 G65C LC-MS

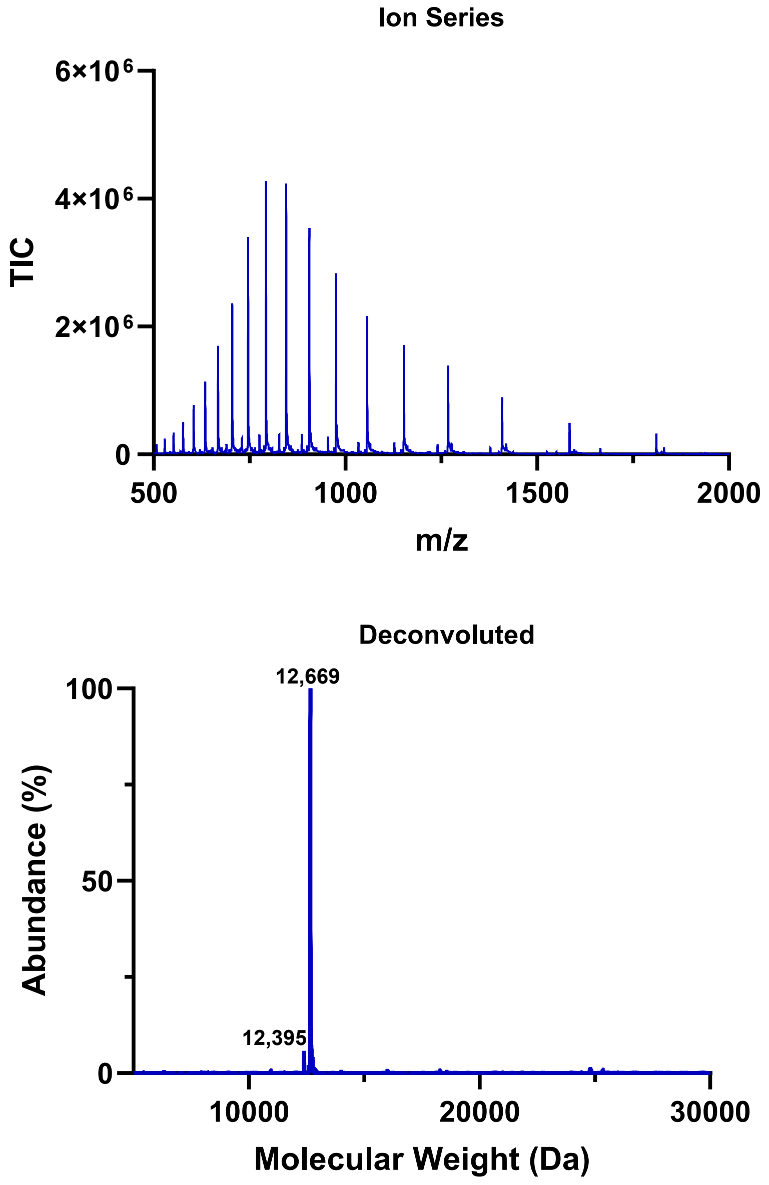

Figure S6: LC–MS spectra of G65C ion series and deconvolution. Calculated molecular weight: 12,668.08 Da. M – 274 Da corresponds to truncation of the C-terminal 6xHis tag by two histidines.

3.1.5 AWT LC-MS

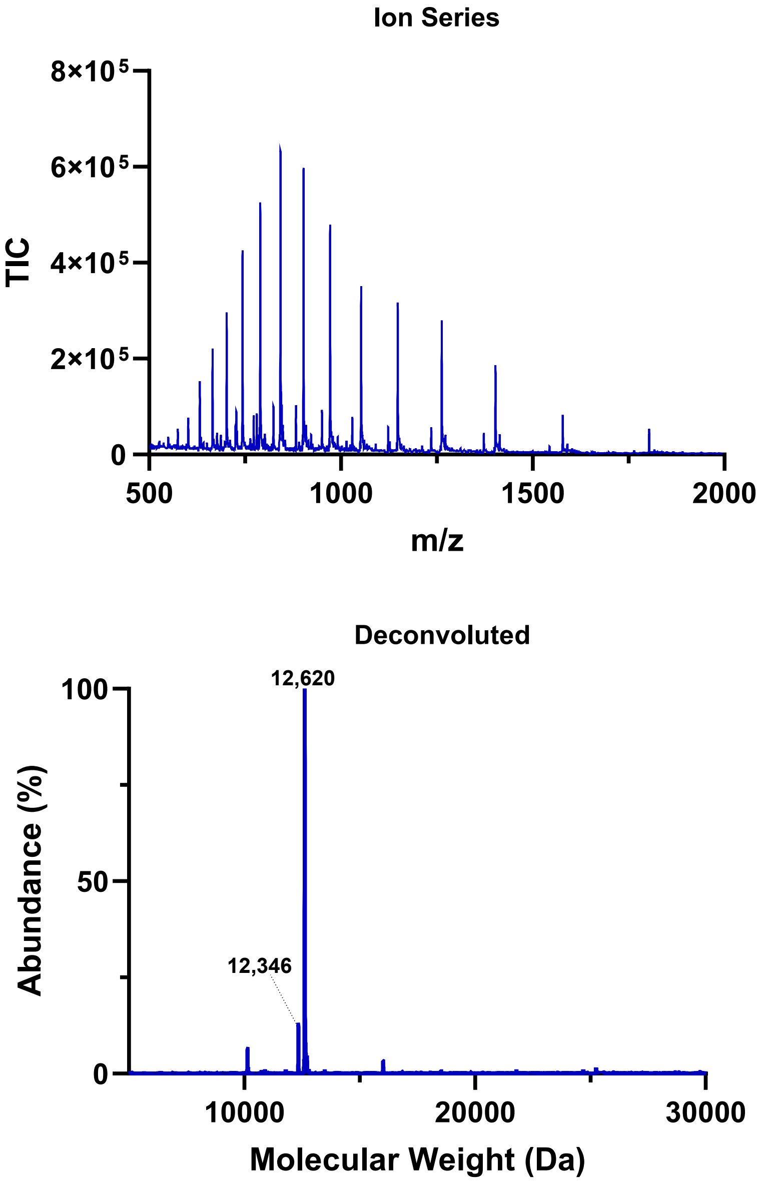

Figure S7: LC–MS spectra of AWT ion series and deconvolution. Calculated molecular weight: 12,621.99 Da. M – 274 Da corresponds to truncation of the C-terminal 6xHis tag by two histidines.

3.1.6 BWT LC-MS

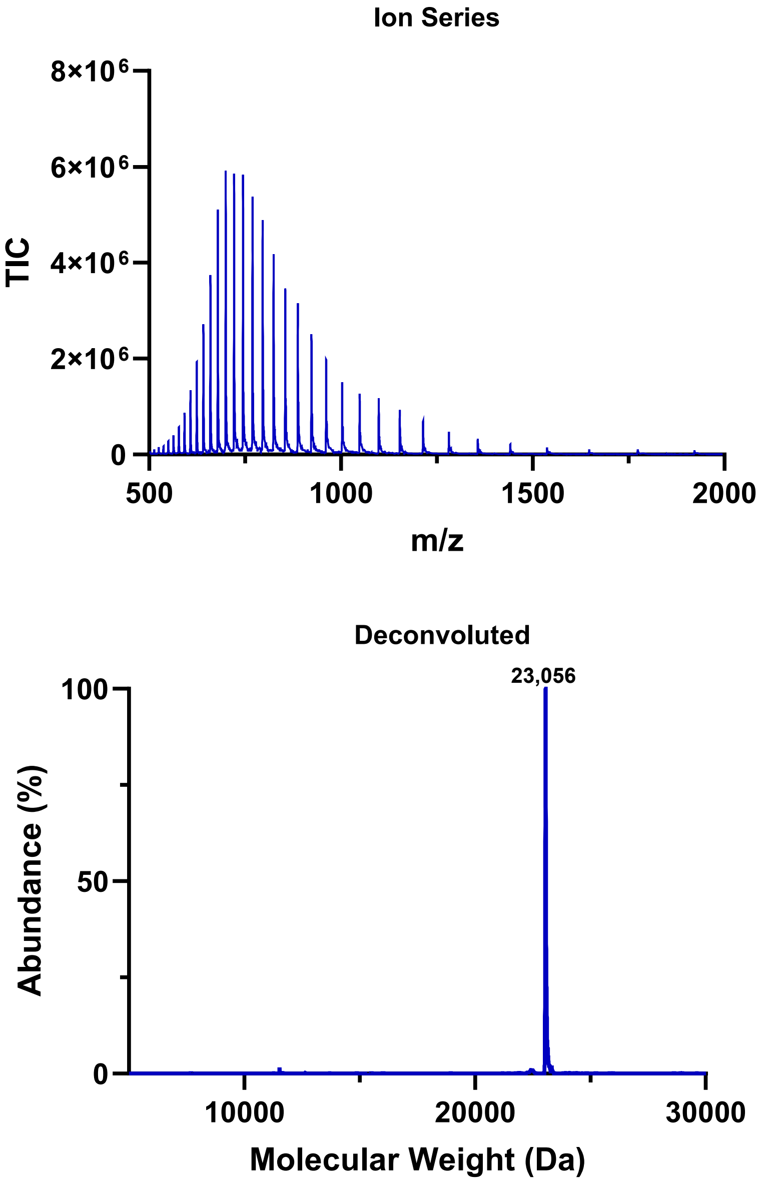

Figure S8: LC–MS spectra of BWT ion series and deconvolution. Calculated molecular weight: 23,057.63 Da.

3.1.7 BH-E62C LC-MS

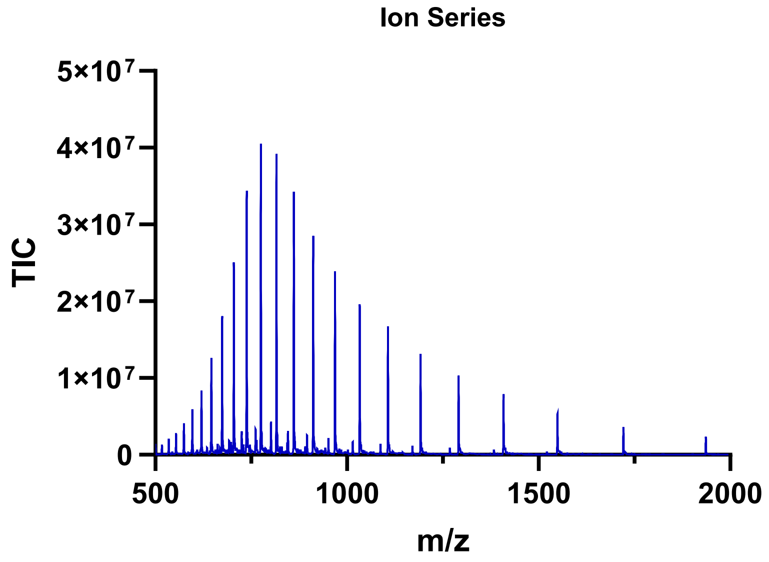

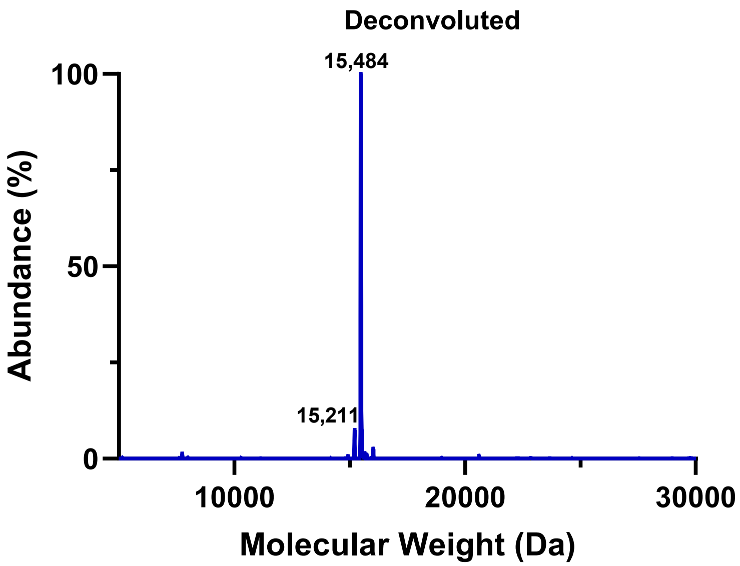

Figure S9: LC–MS spectra of BH-E62C ion series and deconvolution. Calculated molecular weight: 15,480.44 Da. M – 273 Da corresponds to truncation of the C-terminal 6xHis tag by two histidines.

3.1.8 BH-AWT LC-MS

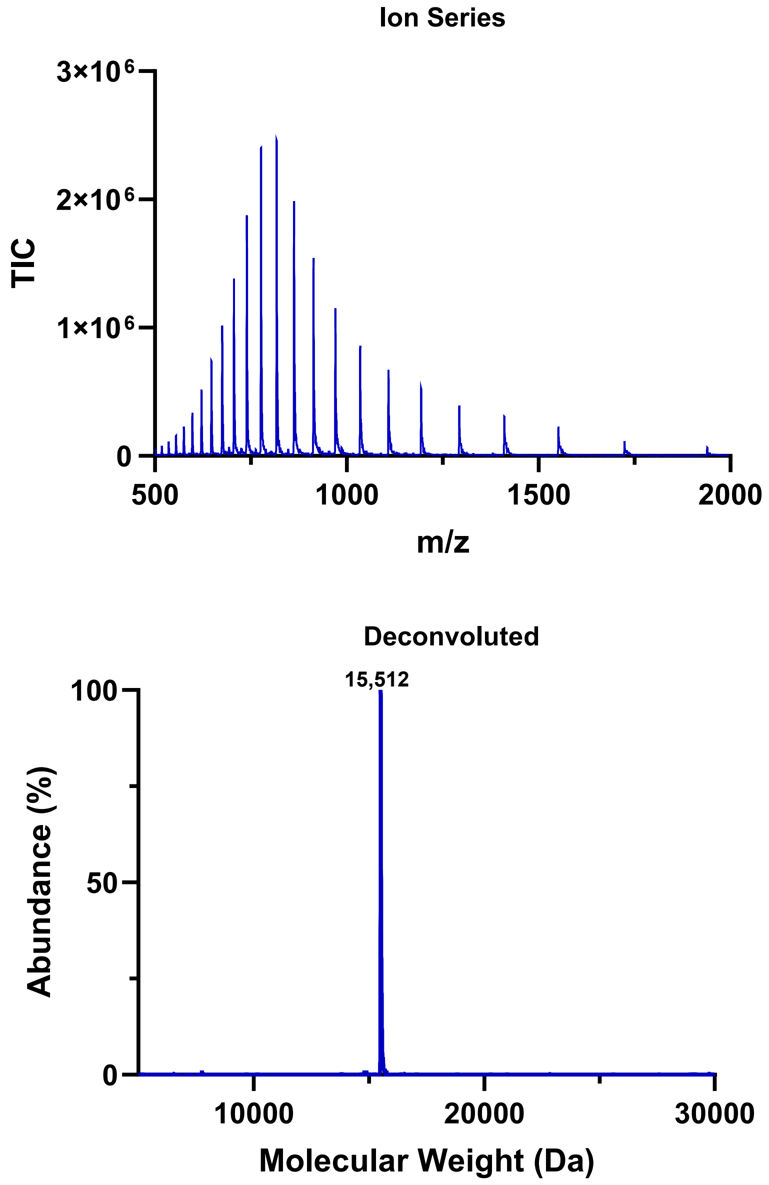

Figure S10: LC–MS spectra of BH-AWT ion series and deconvolution. Calculated molecular weight: 15,506.42 Da.

3.1.9 BH-BWT LC-MS

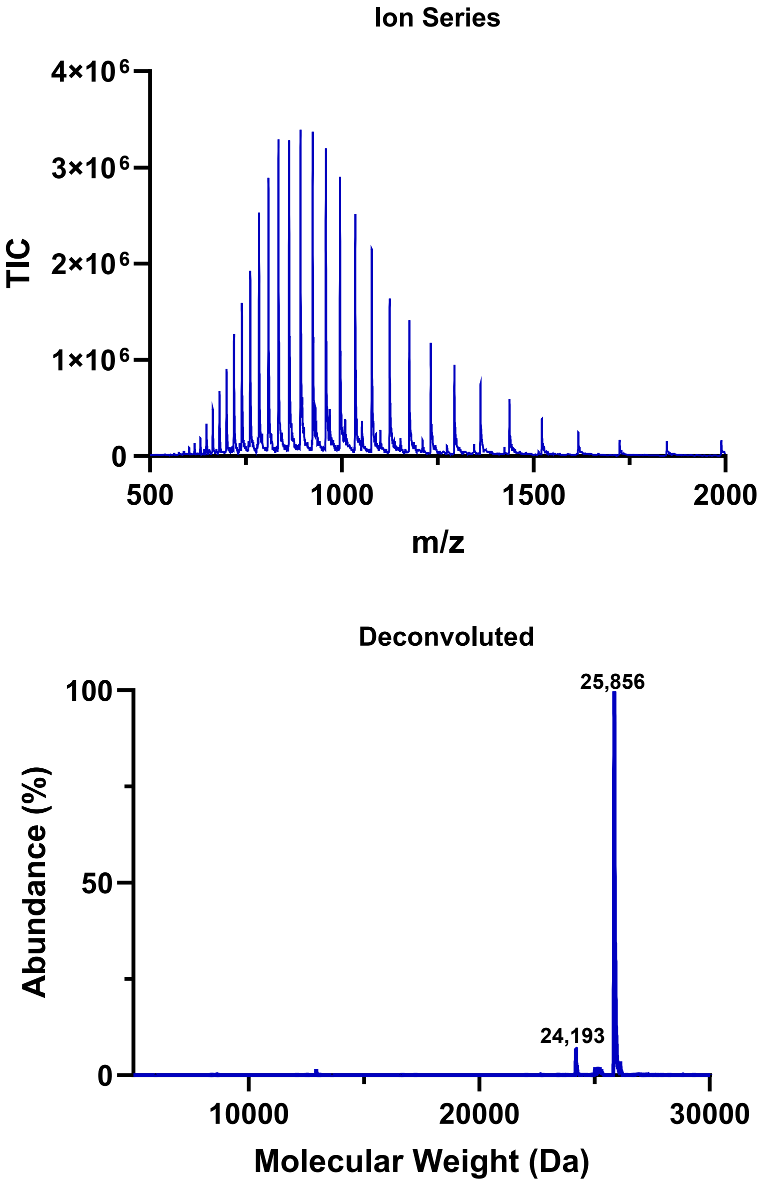

Figure S11: LC–MS spectra of BH-BWT ion series and deconvolution. Calculated molecular weight: 25,847.74 Da. M – 1,663 Da corresponds to N-terminal translation with missing S-tag starting at residue D15 (M14 is cleaved).

3.2 LC-MS spectra of bioconjugates

3.2.1 BC01 (E60C-L1) LC-MS

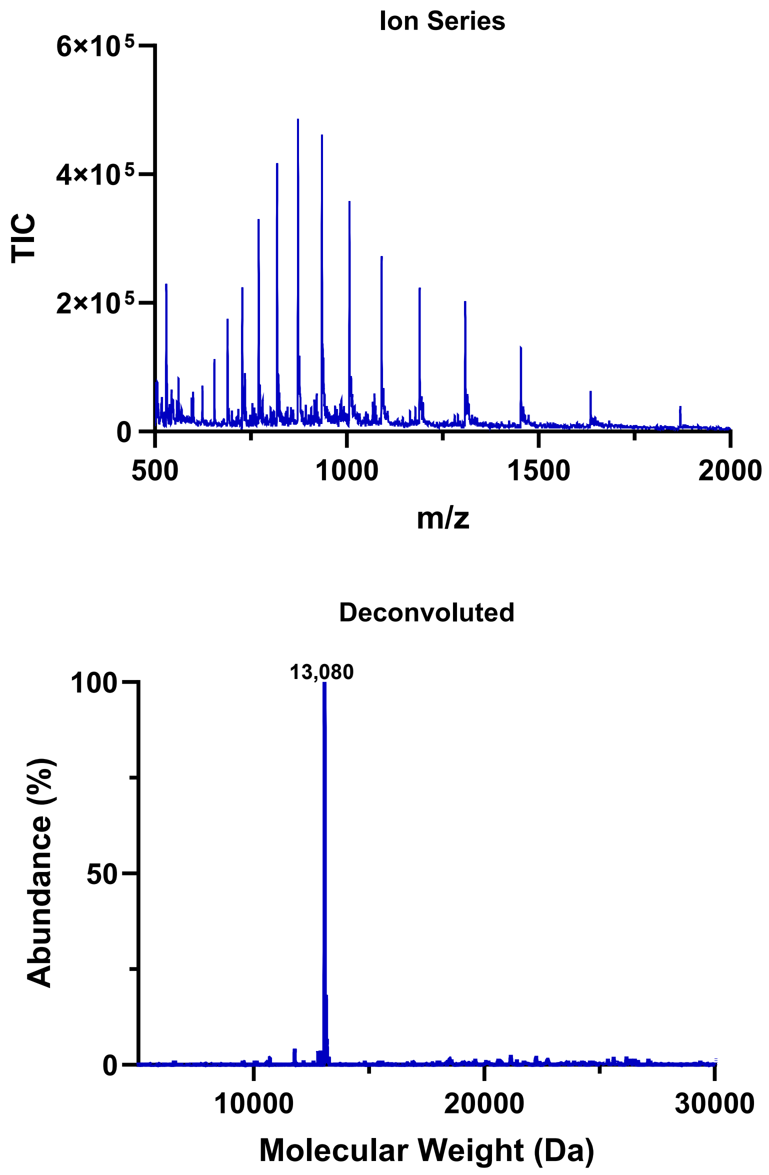

Figure S12: LC–MS spectra of **BC01** (E60C-L1) ion series and deconvolution. Calculated molecular weight: 13,079 Da.

3.2.2 BC02 (E60C-L1-RNA) LC-MS

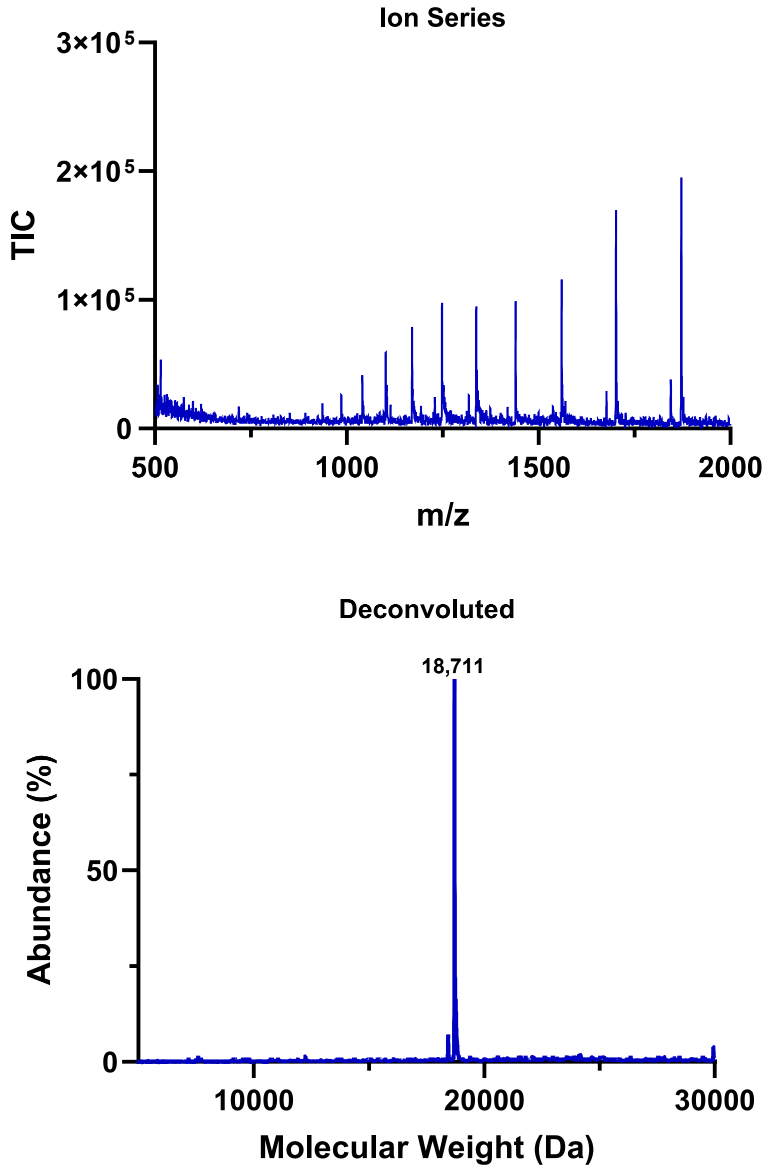

Figure S13: LC–MS spectra of **BC02** (E60C-L1-RNA) ion series and deconvolution. Calculated molecular weight: 18,714 Da.

3.2.3 BC03 (E60C-L2) LC-MS

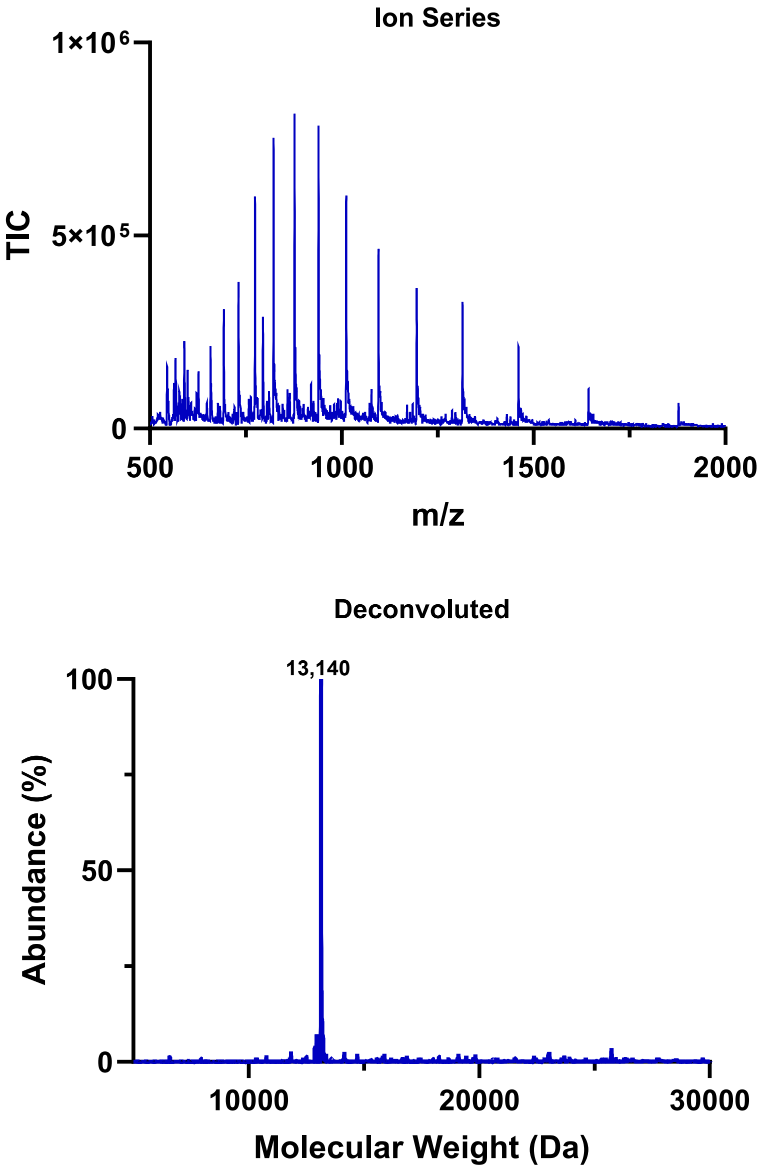

Figure S14: LC–MS spectra of **BC03** (E60C-L2) ion series and deconvolution. Calculated molecular weight: 13,139 Da.

3.2.4 BC04 (E60C-L2-RNA) LC-MS

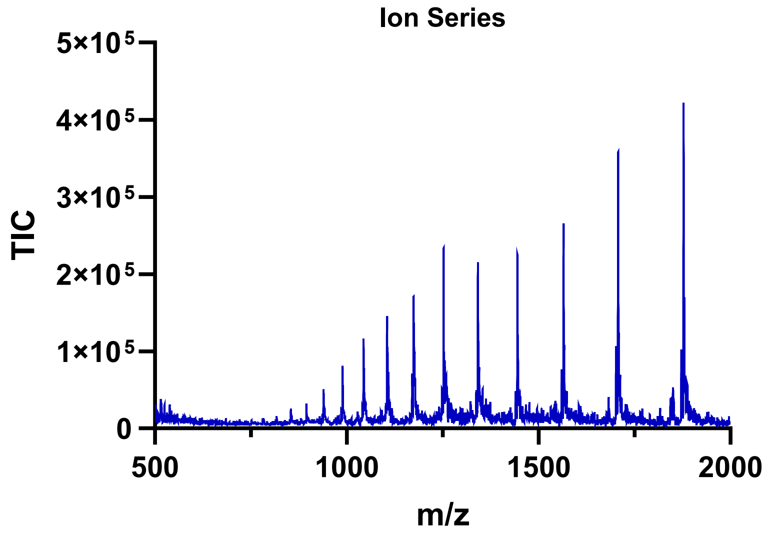

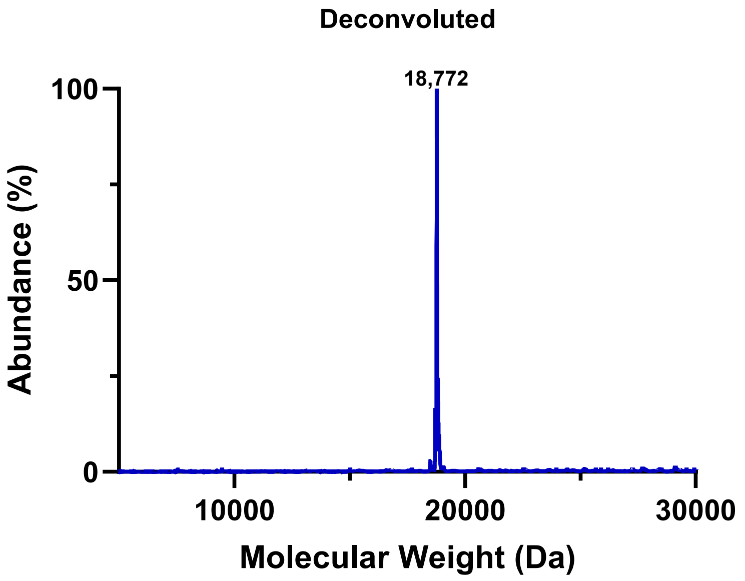

Figure S15: LC–MS spectra of **BC04** (E60C-L2-RNA) ion series and deconvolution. Calculated molecular weight: 18,774 Da.

3.2.5 BC05 (I61C-L1) LC-MS

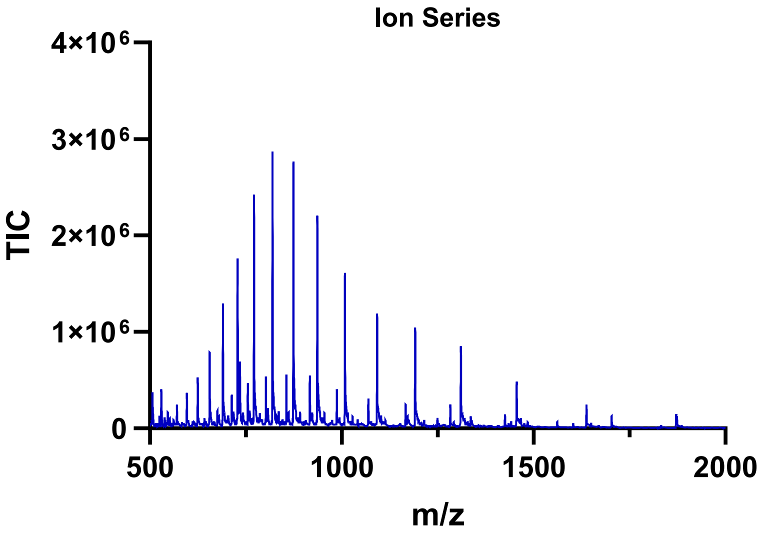

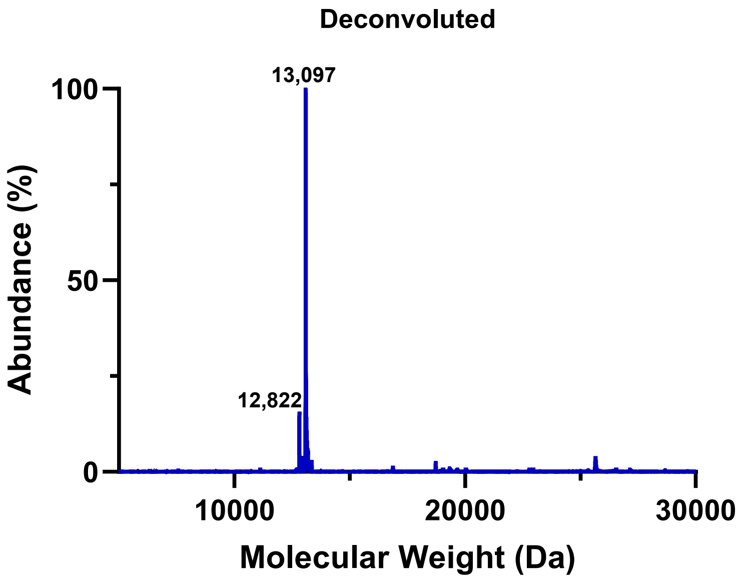

Figure S16: LC–MS spectra of **BC05** (I61C-L1) ion series and deconvolution. Calculated molecular weight: 13,095 Da. M – 275 Da corresponds to truncation of the C-terminal 6xHis tag by two histidines.

3.2.6 BC06 (I61C-L1-RNA) LC-MS

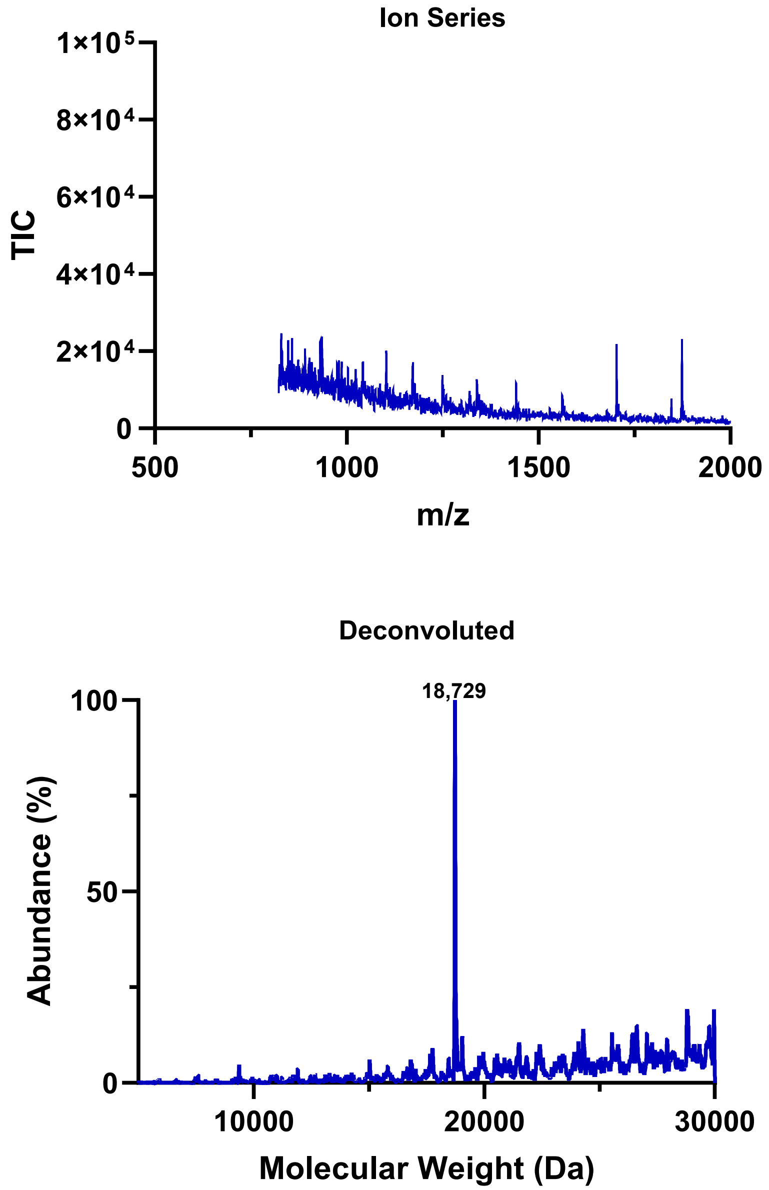

Figure S17: LC–MS spectra of **BC06** (I61C-L1-RNA) ion series and deconvolution. Calculated molecular weight: 18,730 Da.

3.2.7 BC07 (I61C-L2) LC-MS

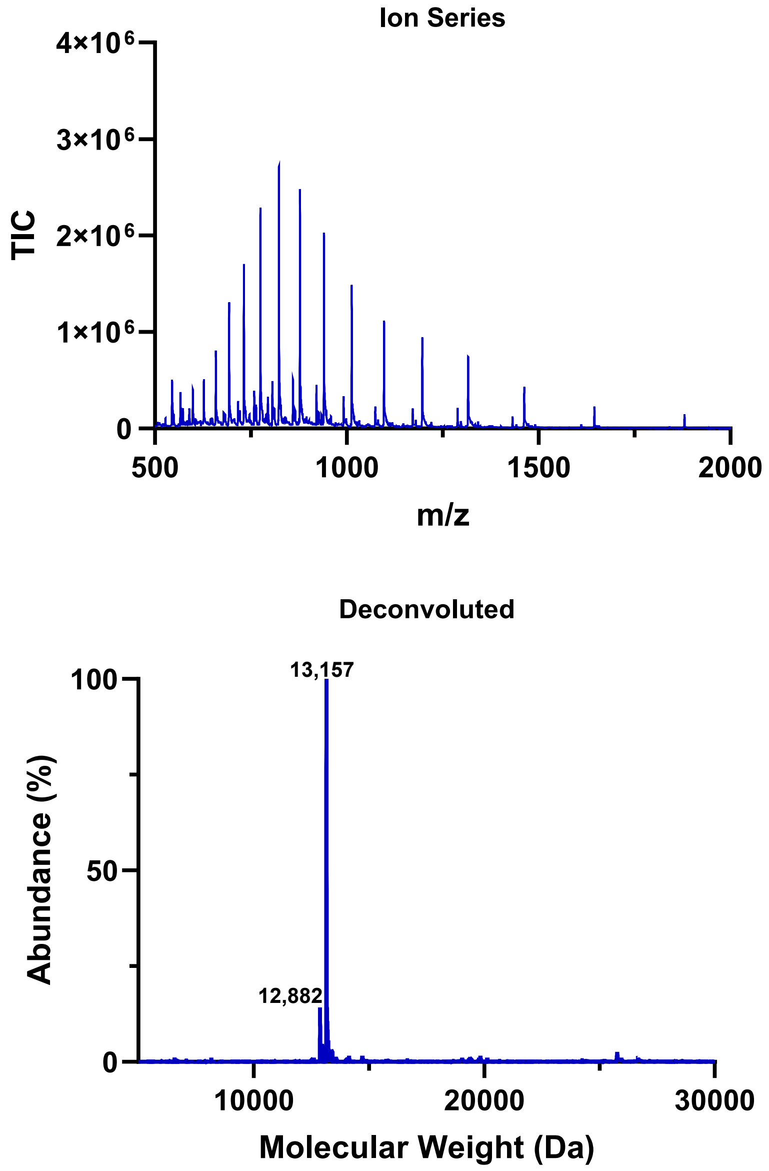

Figure S18: LC–MS spectra of **BC07** (I61C-L2) ion series and deconvolution. Calculated molecular weight: 13,155 Da. M – 275 Da corresponds to truncation of the C-terminal 6xHis tag by two histidines.

3.2.8 BC08 (I61C-L2-RNA) LC-MS

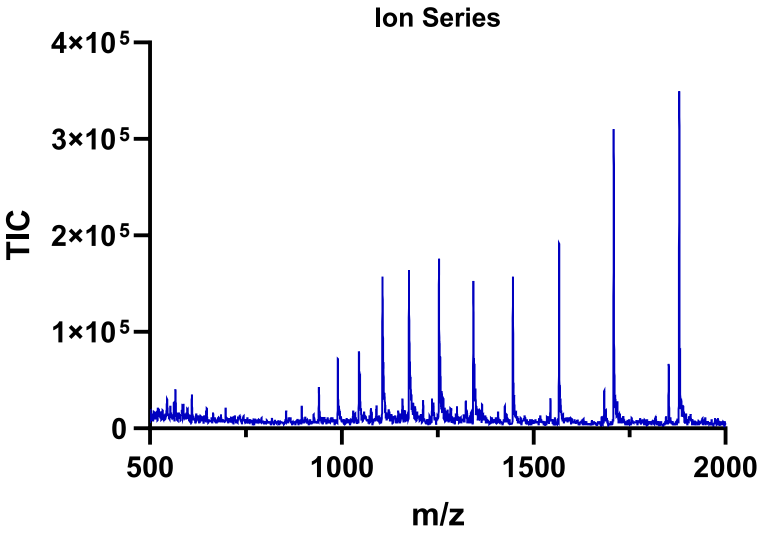

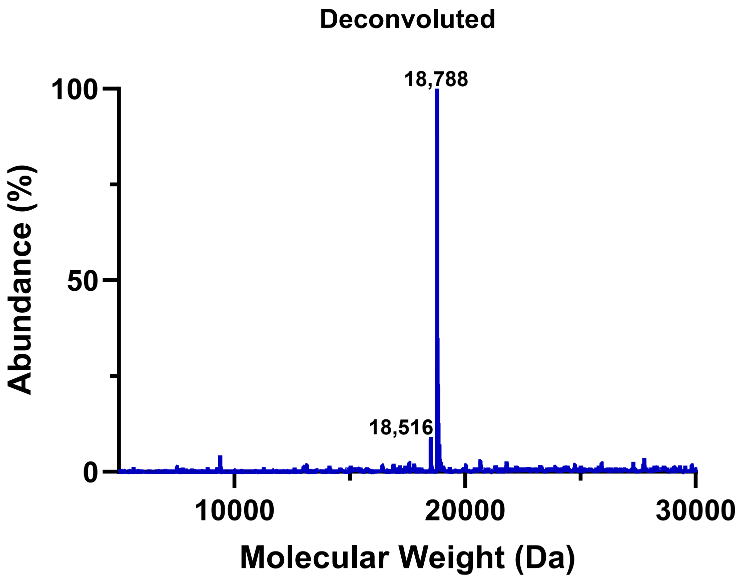

Figure S19: LC–MS spectra of **BC08** (I61C-L2-RNA) ion series and deconvolution. Calculated molecular weight: 18,790 Da. M – 272 Da corresponds to truncation of the C-terminal 6xHis tag by two histidines.

3.2.9 BC09 (E62C-L1) LC-MS

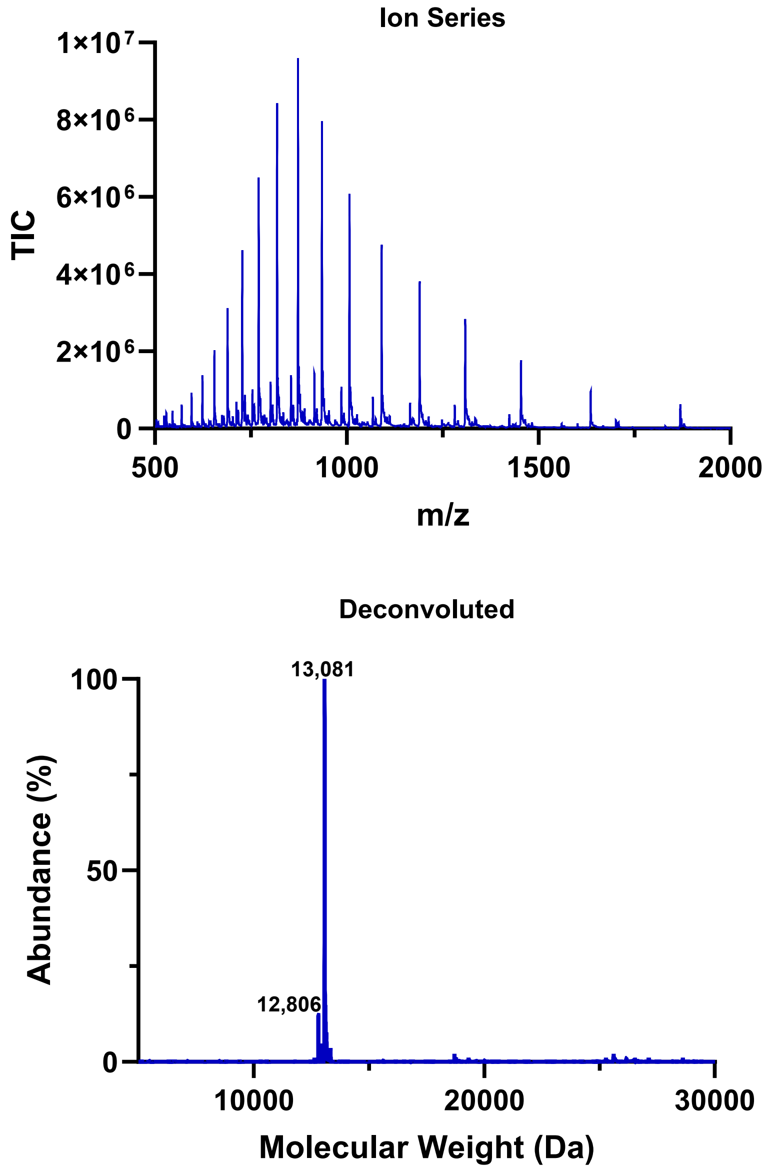

Figure S20: LC–MS spectra of **BC09** (E62C-L1) ion series and deconvolution. Calculated molecular weight: 13,079 Da. M – 275 Da corresponds to truncation of the C-terminal 6xHis tag by two histidines.

3.2.10 BC10 (E62C-L1-RNA) LC-MS

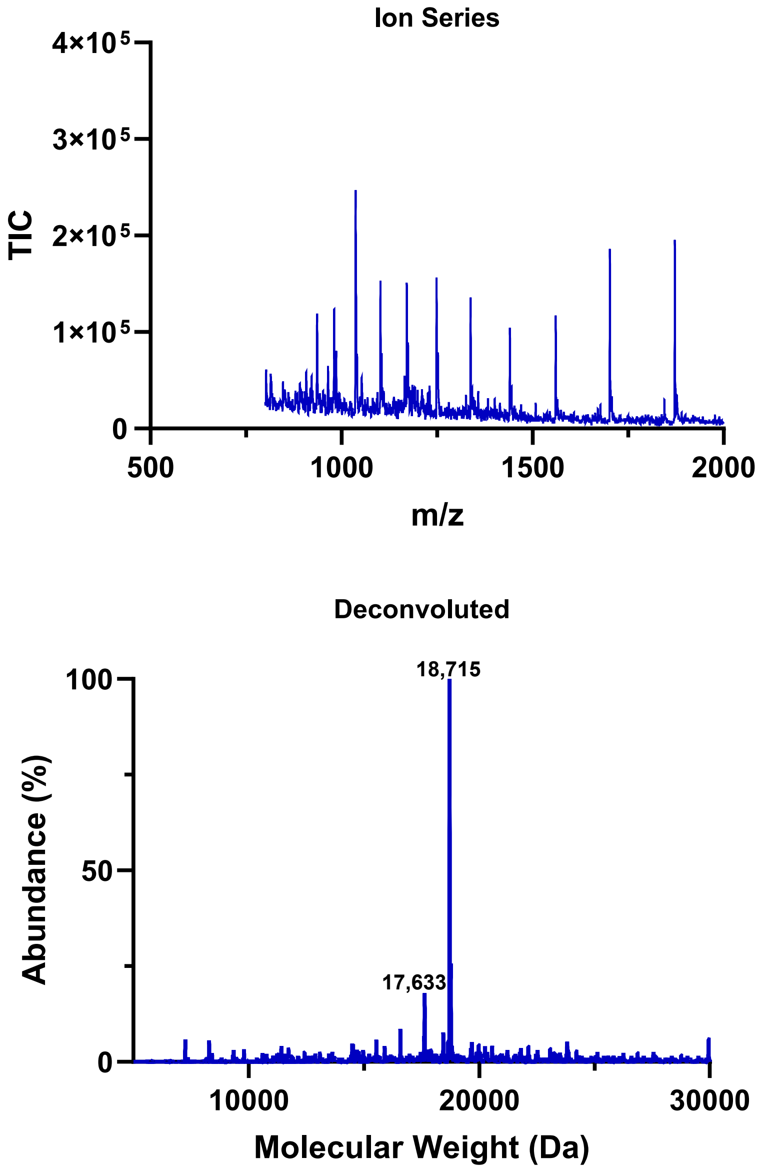

Figure S21: LC–MS spectra of **BC10** (E62C-L1-RNA) ion series and deconvolution. Calculated molecular weight: 18,714 Da. M – 1,082 Da corresponds to loss of N-terminus of protein between residues K11 and S12 which is spatially separated from the binding site.

3.2.11 BC11 (E62C-L2) LC-MS

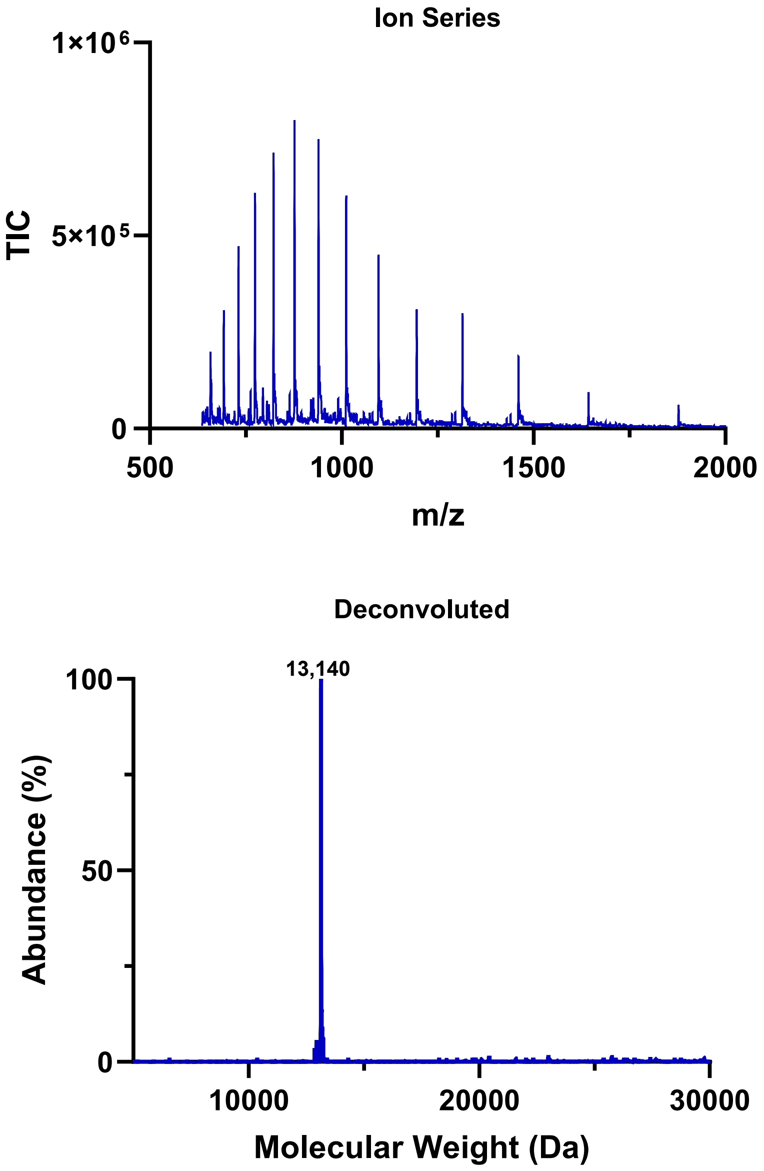

Figure S22: LC–MS spectra of **BC11** (E62C-L2) ion series and deconvolution. Calculated molecular weight: 13,139 Da.

3.2.12 BC12 (E62C-L2-RNA) LC-MS

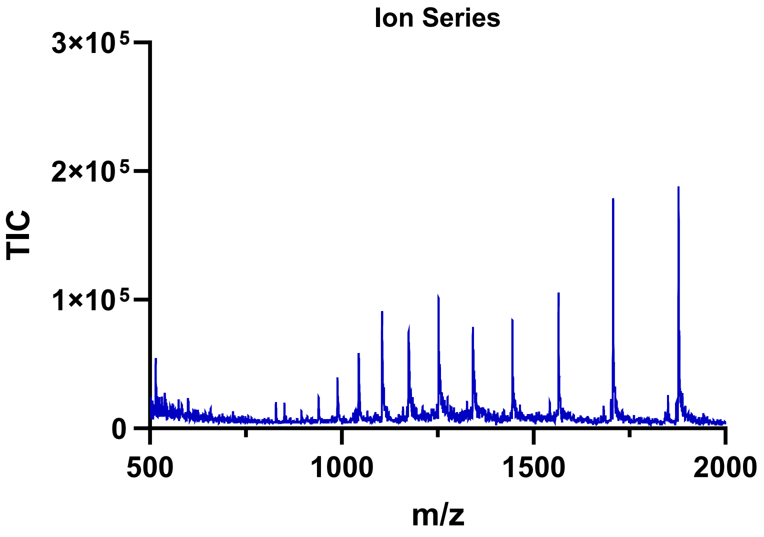

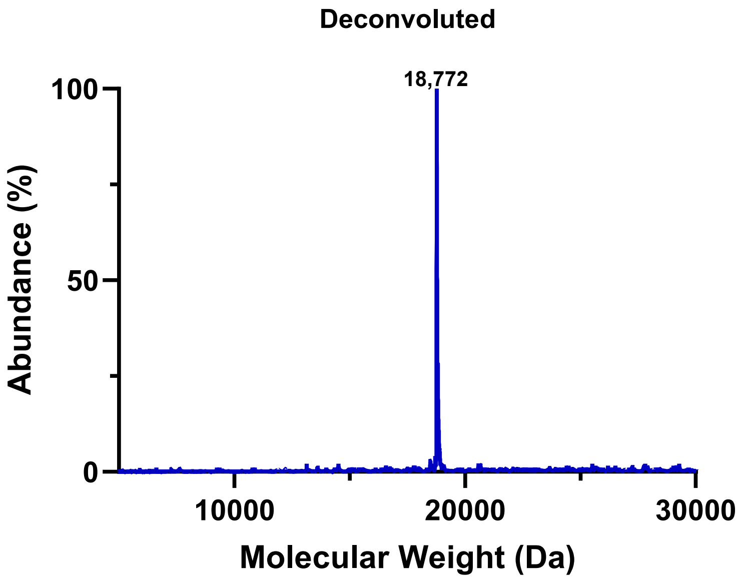

Figure S23: LC–MS spectra of **BC12** (E62C-L2-RNA) ion series and deconvolution. Calculated molecular weight: 18,774 Da.

3.2.13 BC13 (G65C-L1) LC-MS

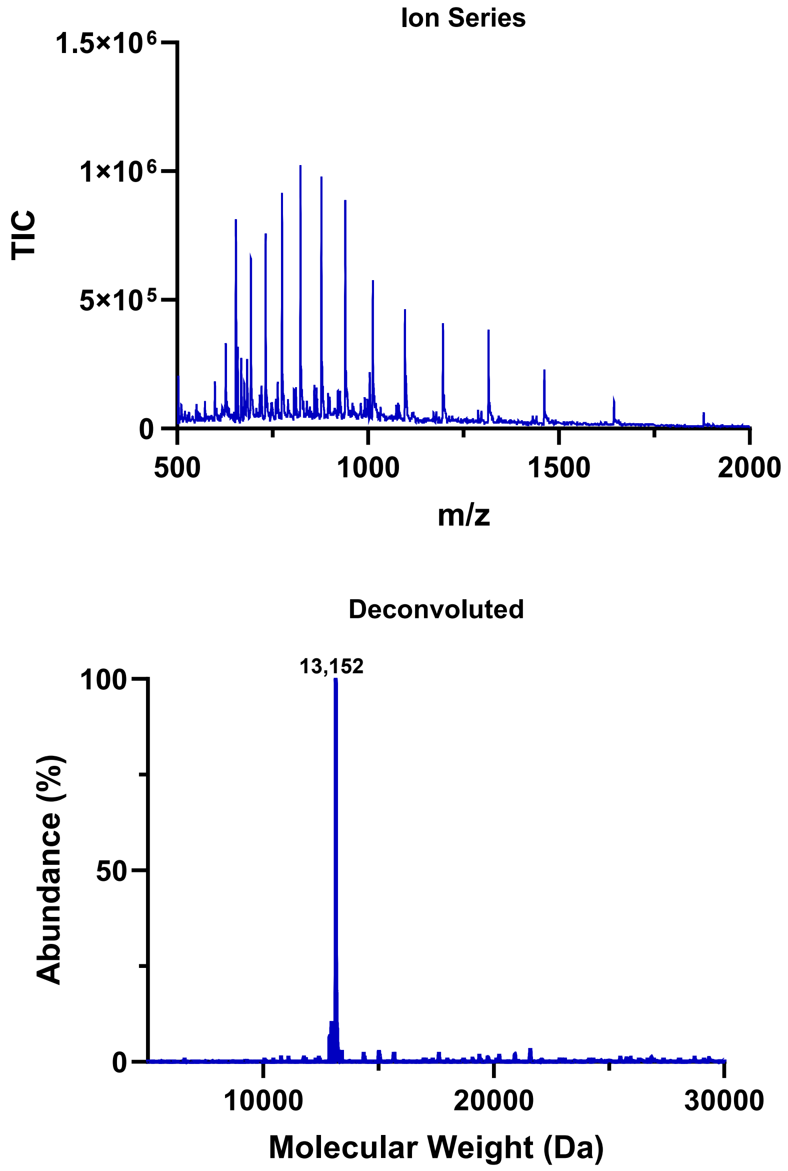

Figure S24: LC–MS spectra of **BC13** (G65C-L1) ion series and deconvolution. Calculated molecular weight: 13,151 Da.

3.2.14 BC14 (G65C-L1-RNA) LC-MS

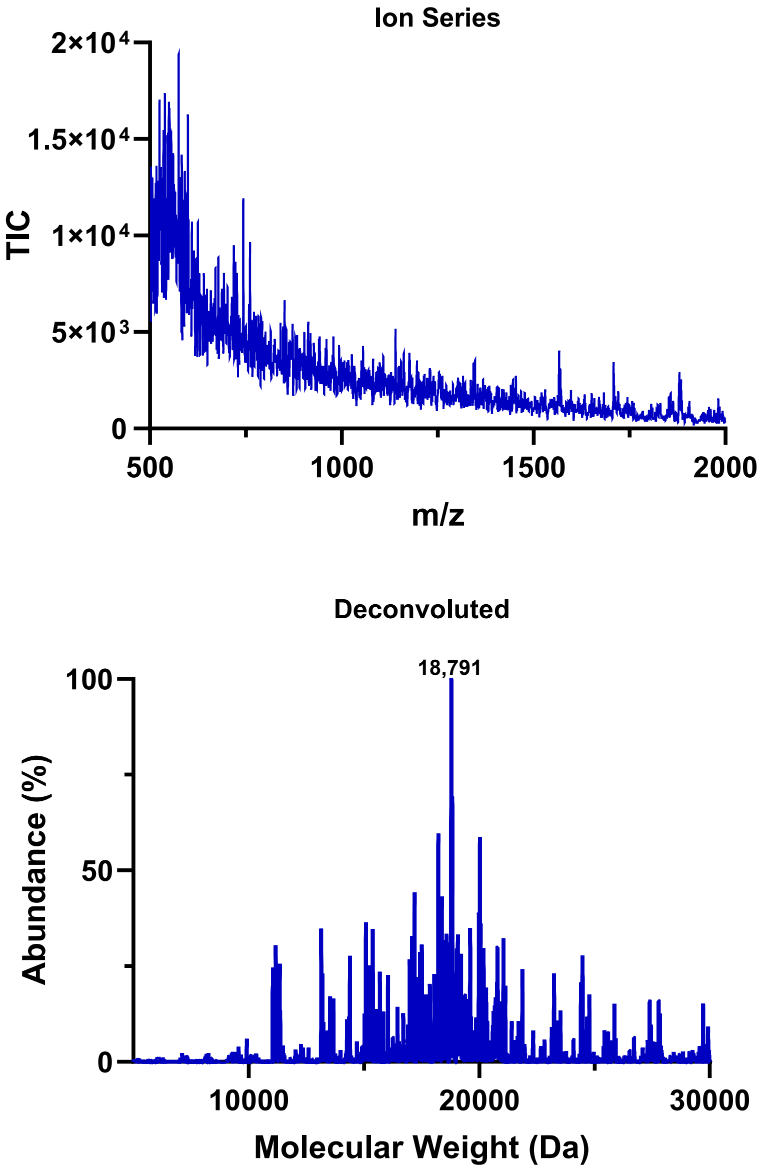

Figure S25: LC–MS spectra of **BC14** (G65C-L1-RNA) ion series and deconvolution. Calculated molecular weight: 18,786 Da. Deconvoluted spectrum was produced from only 1,000–2,000 m/z of the ion series.

3.2.15 BC15 (G65C-L2) LC-MS

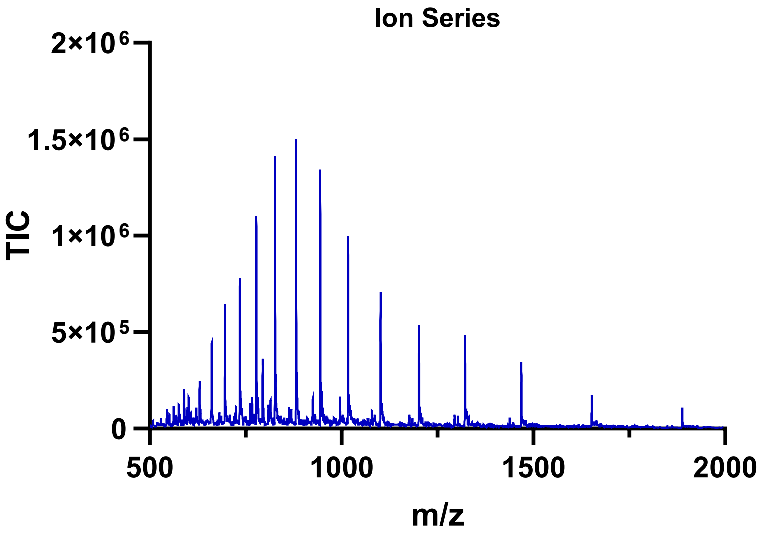

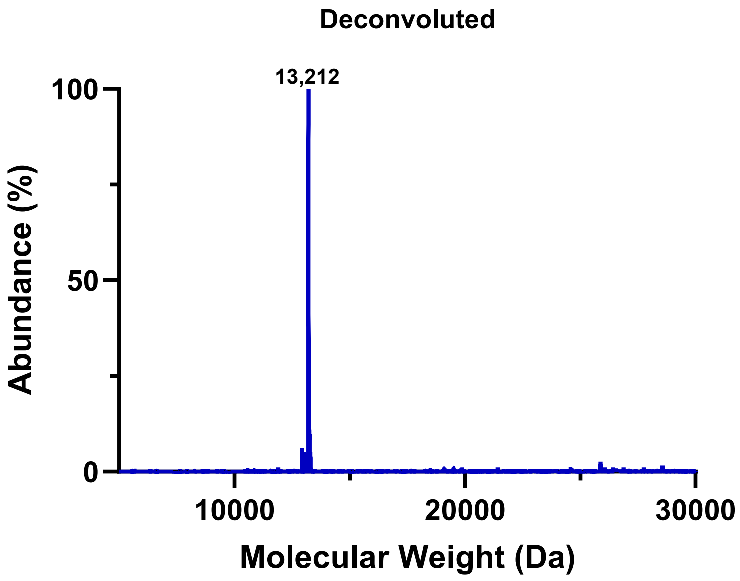

Figure S26: LC–MS spectra of **BC15** (G65C-L2) ion series and deconvolution. Calculated molecular weight: 13,211 Da.

3.2.16 BC16 (G65C-L2-RNA) LC-MS

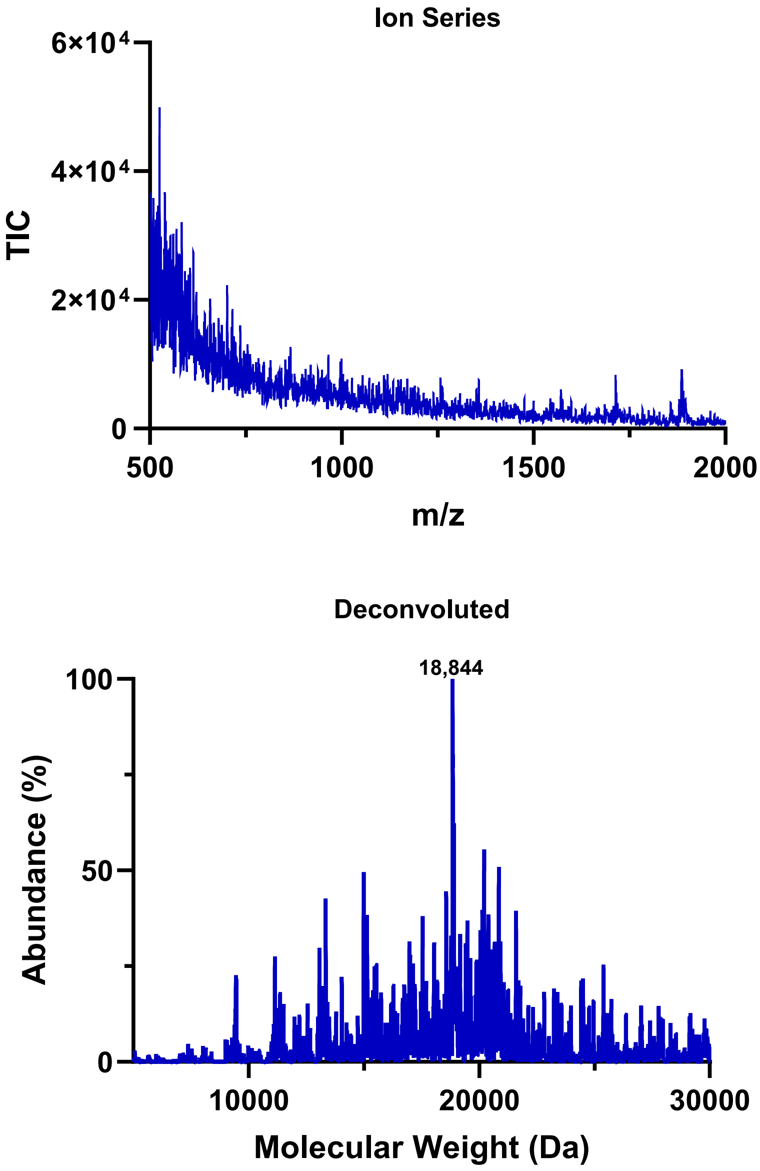

Figure S27: LC–MS spectra of **BC16** (G65C-L2-RNA) ion series and deconvolution. Calculated molecular weight: 18,846 Da. Deconvoluted spectrum was produced from only 1,000–2,000 m/z of the ion series.

3.2.17 BC17 (BH-E62C-L1) LC-MS

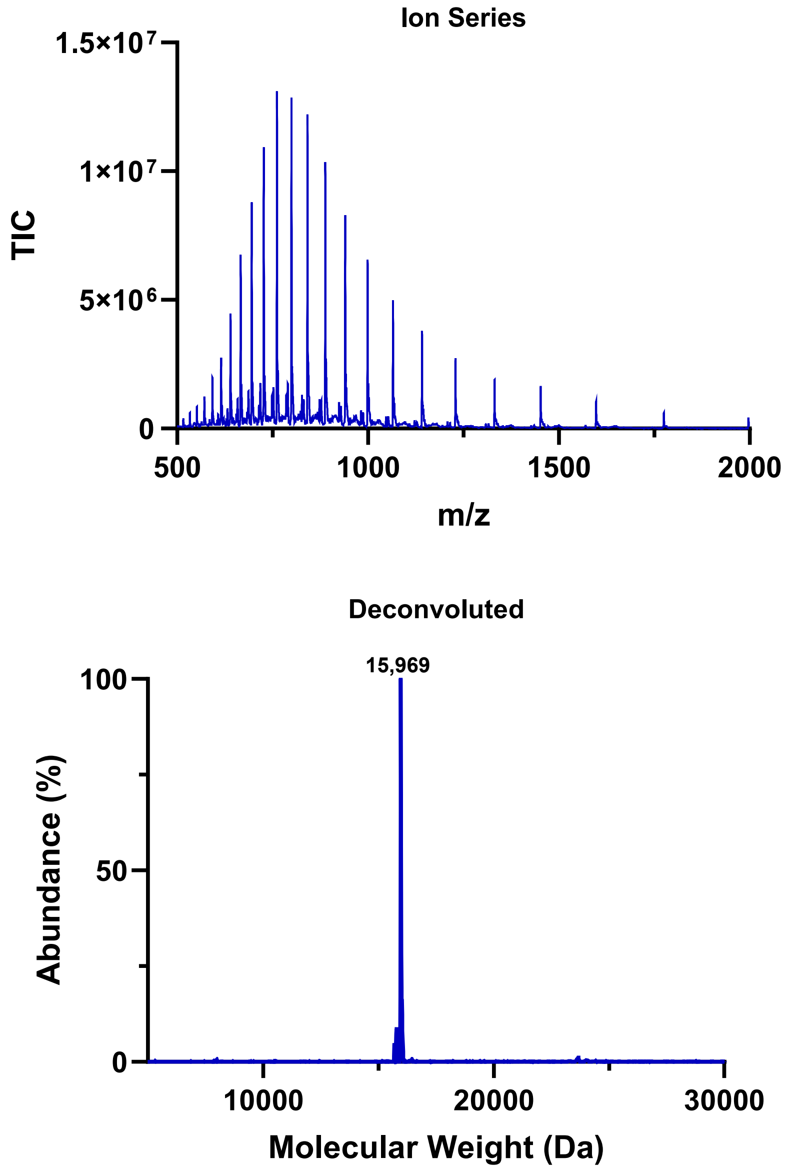

Figure S28: LC–MS spectra of **BC17** (BH-E62C-L1) ion series and deconvolution. Calculated molecular weight: 15,963 Da.

3.2.18 BC18 (BH-E62C-L1-RNA) LC-MS

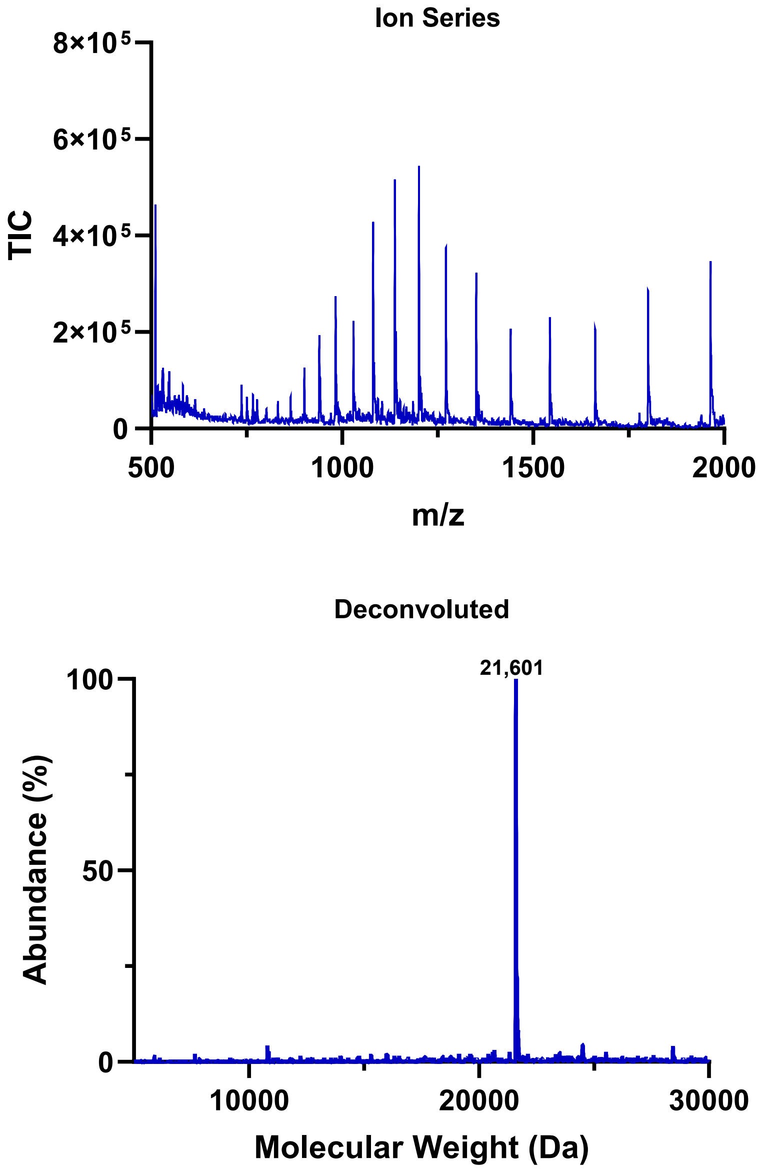

Figure S29: LC–MS spectra of **BC18** (BH-E62C-L1-RNA) ion series and deconvolution. Calculated molecular weight: 21,598 Da.

4. Size exclusion chromatography (SEC) of protein-RNA bioconjugates

4.1 BC02 (E60C-L1-RNA) SEC trace

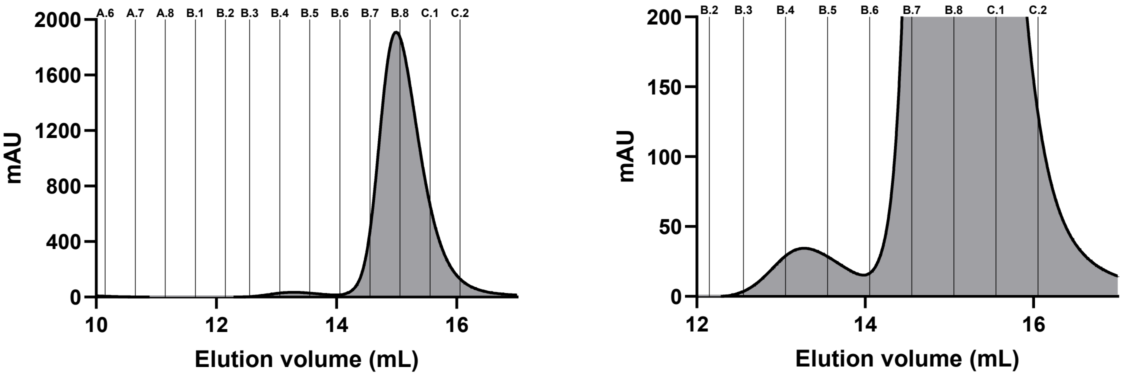

Figure S30: FPLC size exclusion chromatogram trace for **BC02** (E60C-L1-RNA) with Superdex75 Increase 10/300 GL. Absorption is measured at 280 nm. Peak 1 (12–14 min) corresponds to the bioconjugate and peak 2 (14–17 min) corresponds to excess, unreacted **conjRNA1**. Fractions B.3–B.5 were pooled and used in all further testing.

4.2 BC04 (E60C-L2-RNA) SEC trace

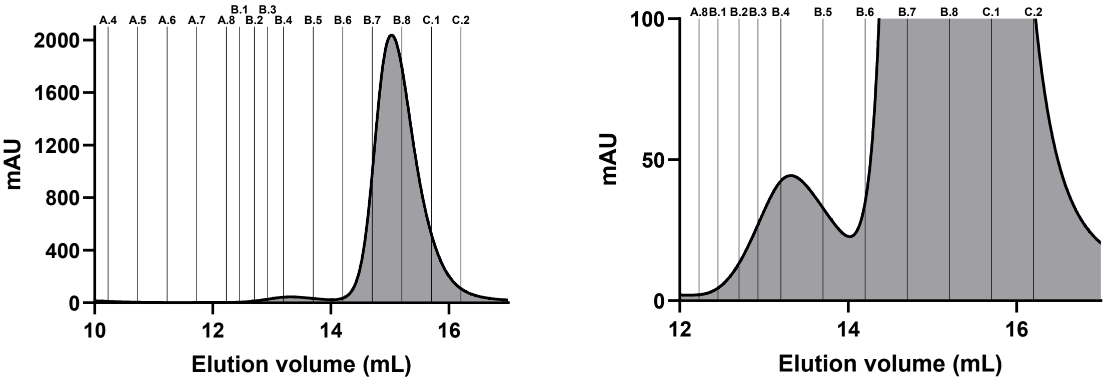

Figure S31: FPLC size exclusion chromatogram trace for **BC04** (E60C-L2-RNA) with Superdex75 Increase 10/300 GL. Absorption is measured at 280 nm. Peak 1 (12–14 min) corresponds to the bioconjugate and peak 2 (14–17 min) corresponds to excess, unreacted **conjRNA1**. Fractions B.1–B.5 were pooled and used in all further testing.

4.3 BC06 (I61C-L1-RNA) SEC trace

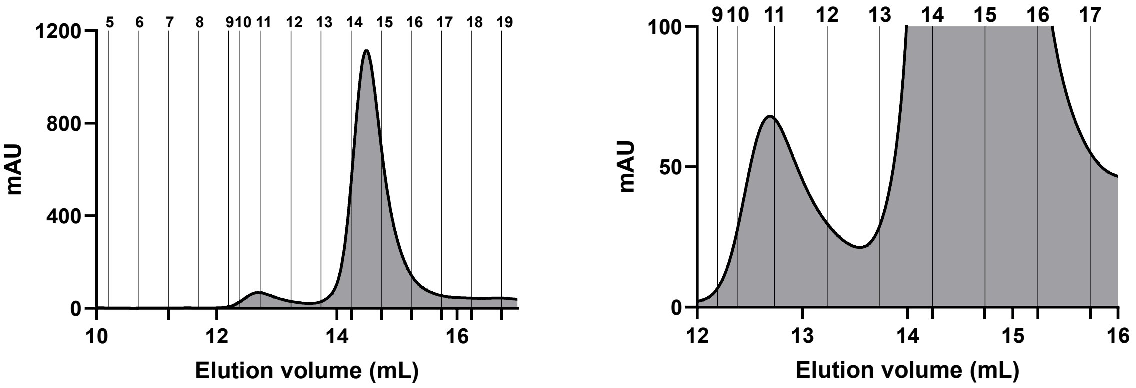

Figure S32: FPLC size exclusion chromatogram trace for **BC06** (I61C-L1-RNA) with Superdex75 Increase 10/300 GL. Absorption is measured at 280 nm. Peak 1 (12–14 min) corresponds to the bioconjugate and peak 2 (14–16 min) corresponds to excess, unreacted **conjRNA1**. Fraction 9 was saved and used in all further testing.

4.4 BC08 (I61C-L2-RNA) SEC trace

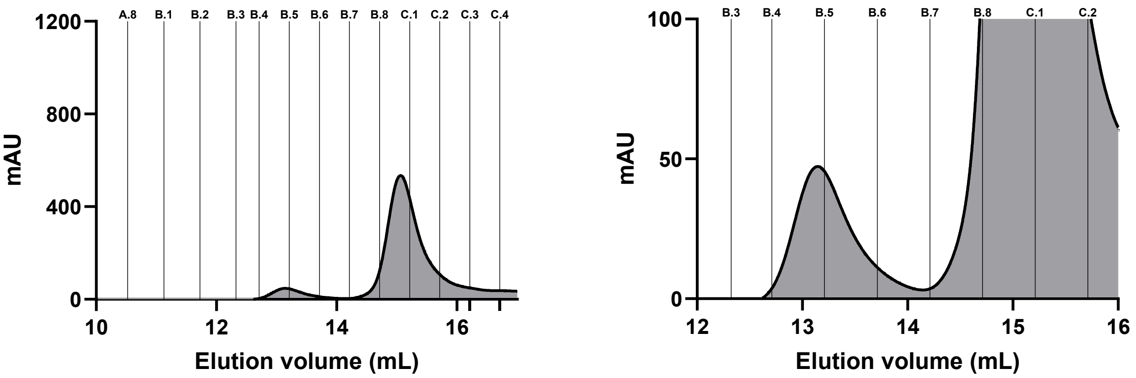

Figure S33: FPLC size exclusion chromatogram trace for **BC08** (I61C-L2-RNA) with Superdex75 Increase 10/300 GL. Absorption is measured at 280 nm. Peak 1 (12–14 min) corresponds to the bioconjugate and peak 2 (14–16 min) corresponds to excess, unreacted **conjRNA1**. Fraction B.4 was saved and used in all further testing.

4.5 BC10 (E62C-L1-RNA) SEC trace

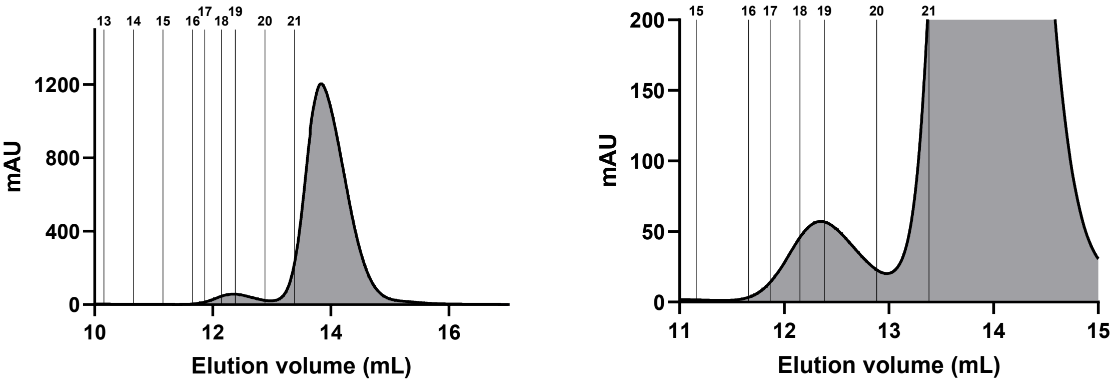

Figure S34: FPLC size exclusion chromatogram trace for **BC10** (E62C-L1-RNA) with Superdex75 Increase 10/300 GL. Absorption is measured at 280 nm. Peak 1 (11.5–13 min) corresponds to the bioconjugate and peak 2 (13–15 min) corresponds to excess, unreacted **conjRNA1**. Fractions 16–19 were saved individually and were used in all further testing.

4.6 BC12 (E62C-L2-RNA) SEC trace

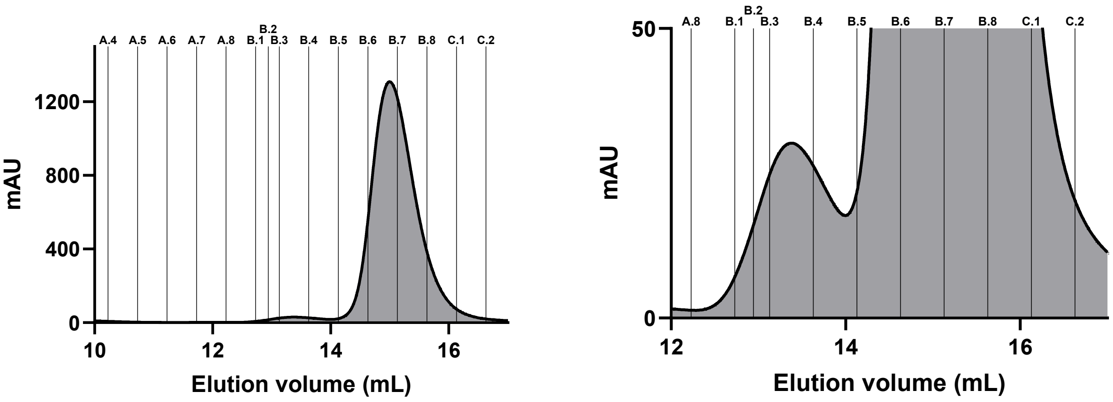

Figure S35: FPLC size exclusion chromatogram trace for **BC12** (E62C-L2-RNA) with Superdex75 Increase 10/300 GL. Absorption is measured at 280 nm. Peak 1 (12–14 min) corresponds to the bioconjugate and peak 2 (14–16 min) corresponds to excess, unreacted **conjRNA1**. Fractions B.1–B.4 were saved individually and were used in all further testing.

4.7 BC14 (G65C-L1-RNA) SEC trace

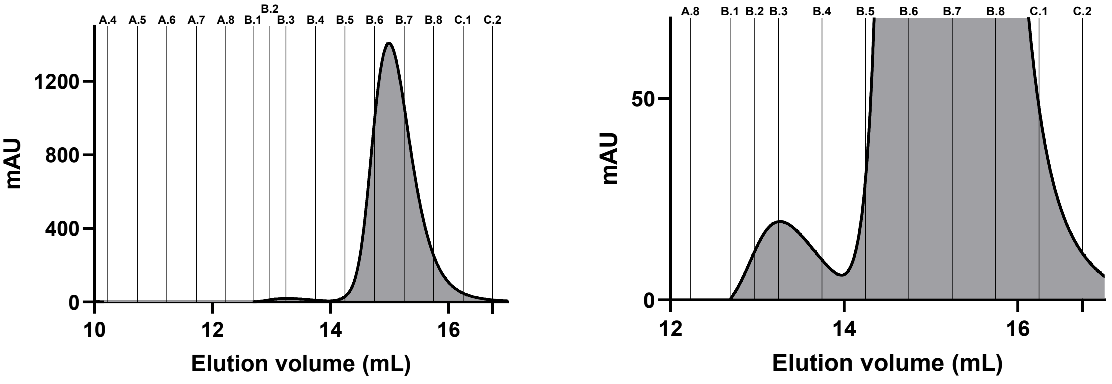

Figure S36: FPLC size exclusion chromatogram trace for **BC14** (G65C-L1-RNA) with Superdex75 Increase 10/300 GL. Absorption is measured at 280 nm. Peak 1 (12–14 min) corresponds to the bioconjugate and peak 2 (14–16 min) corresponds to excess, unreacted **conjRNA1**. Fractions B.1–B.4 were pooled and were used in all further testing.

4.8 BC16 (G65C-L2-RNA) SEC trace

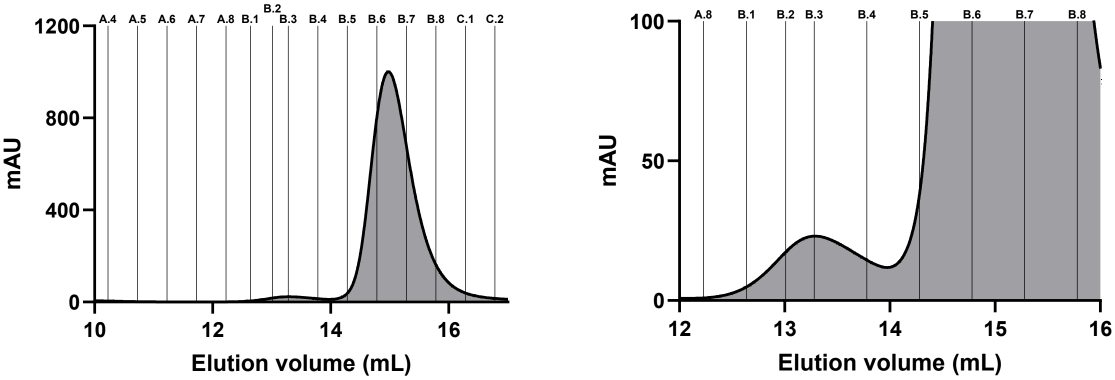

Figure S37: FPLC size exclusion chromatogram trace for **BC16** (G65C-L2-RNA) with Superdex75 Increase 10/300 GL. Absorption is measured at 280 nm. Peak 1 (12–14 min) corresponds to the bioconjugate and peak 2 (14–16 min) corresponds to excess, unreacted **conjRNA1**. Fractions B.1–B.3 were pooled and were used in all further testing.

4.9 BC18 (BH-E62C-L1-RNA) SEC trace

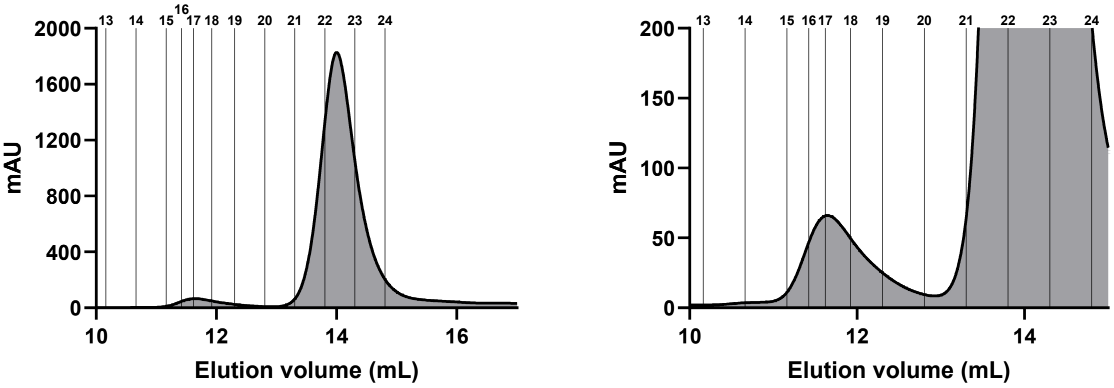

Figure S38: FPLC size exclusion chromatogram trace for **BC18** (BH-E62C-L1-RNA) with Superdex75 Increase 10/300 GL. Absorption is measured at 280 nm. Peak 1 (11–13 min) corresponds to the bioconjugate and peak 2 (13–15 min) corresponds to excess, unreacted **conjRNA1**. Fractions 15–18 were saved individually and were used in all further testing.

5. Gel electrophoresis

5.1 SDS-PAGE characterization of proteins

5.1.1 Gel of E60C and AWT purifications

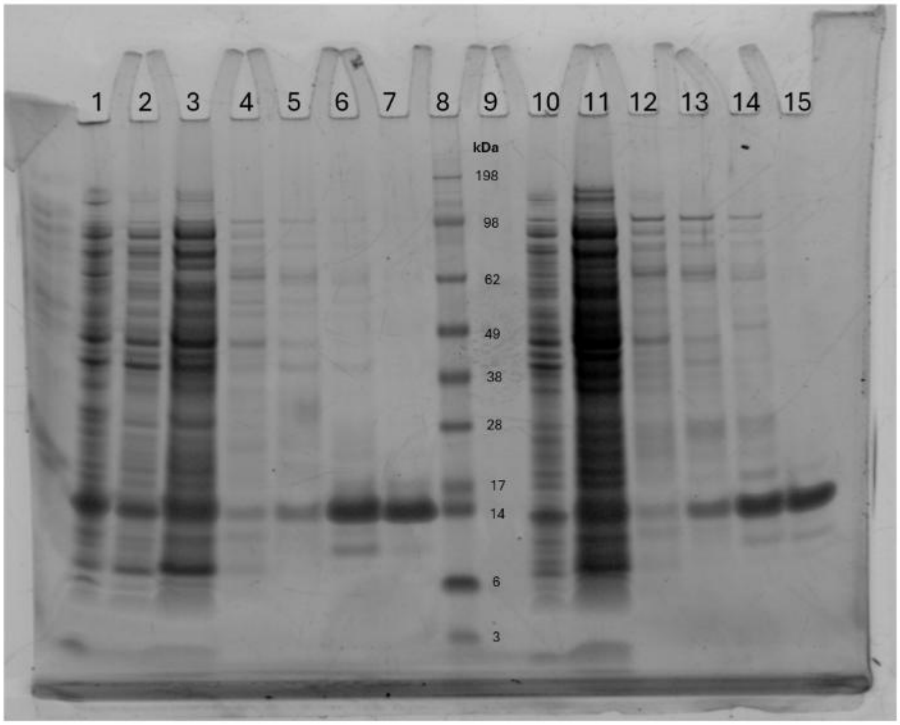

Figure S39: InstantBlue-stained NuPAGE™ gel of E60C and AWT purification. Lane 1: E60C crude lysate. Lane 2: E60C cleared lysate. Lane 3: E60C flow through. Lane 4: E60C wash 1 (20 mM imidazole in 1X PBS pH 7.4). Lane 5: E60C wash 2 (40 mM imidazole in 1X PBS pH 7.4). Lane 6: E60C elution in 500 mM imidazole in 1X PBS pH 7.4 desalted into 1X PBS pH 7.4. Lane 7: E60C after size exclusion. Lane 8: ladder. Lane 9: empty. Lane 10: AWT cleared lysate. Lane 11: AWT flow through. Lane 12: AWT wash 1 (20 mM imidazole in 1X PBS pH 7.4). Lane 13: AWT wash 2 (40 mM imidazole in 1X PBS pH 7.4). Lane 14: AWT elution in 500 mM imidazole in 1X PBS pH 7.4 desalted into 1X PBS pH 7.4. Lane 15: AWT after size exclusion.

5.1.2 Gel of BWT purification

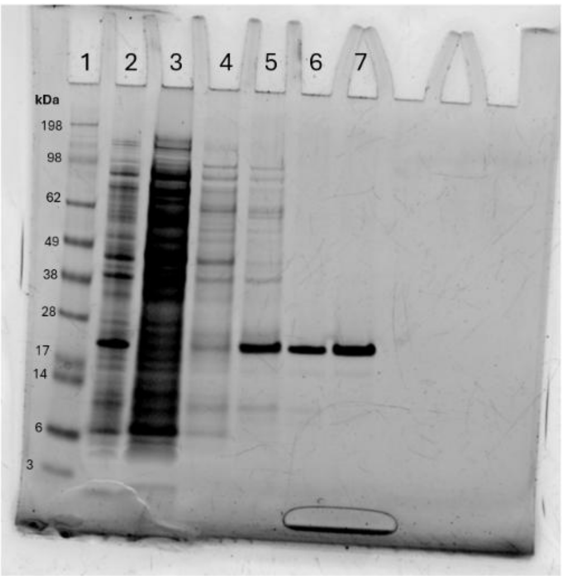

Figure S40: InstantBlue-stained NuPAGE™ gel of BWT purification. Lane 1: ladder. Lane 2: cleared lysate. Lane 3: flow through. Lane 4: wash 1 (20 mM imidazole in 1X PBS pH 7.4). Lane 5: wash 2 (40 mM imidazole in 1X PBS pH 7.4). Lane 6: elution in 500 mM imidazole in 1X PBS pH 7.4 desalted into 1X PBS pH 7.4. Lane 7: after size exclusion.

5.1.3 Gel of I61C purification

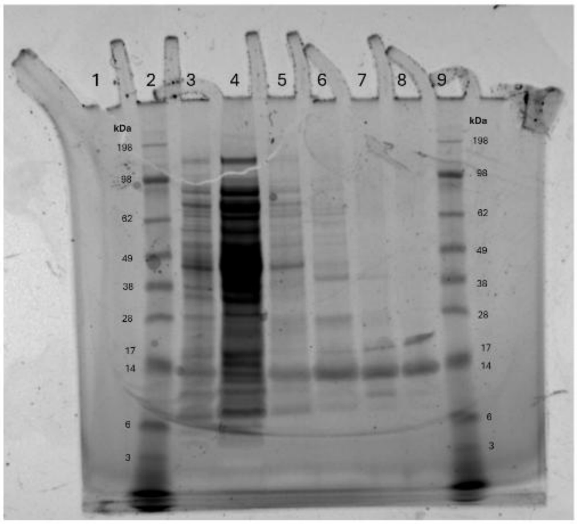

Figure S41: InstantBlue-stained NuPAGE™ gel of I61C purification. Lane 1: empty. Lane 2: ladder. Lane 3: cleared lysate. Lane 4: flow through. Lane 5: wash 1 (20 mM imidazole in 1X PBS pH 7.4). Lane 6: wash 2 (40 mM imidazole in 1X PBS pH 7.4). Lane 7: elution in 500 mM imidazole in 1X PBS pH 7.4. Lane 8: after size exclusion. Lane 9: ladder.

5.1.4 Gel of E62C purification

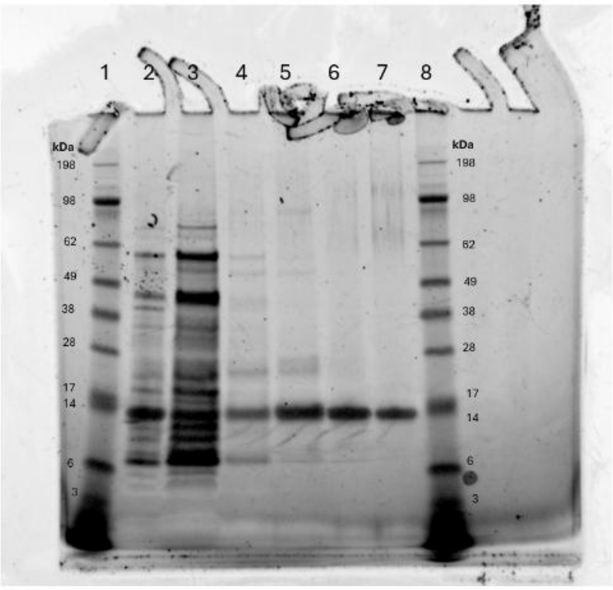

Figure S42: InstantBlue-stained NuPAGE™ gel of E62C purification. Lane 1: ladder. Lane 2: cleared lysate. Lane 3: flow through. Lane 4: wash 1 (20 mM imidazole in 1X PBS pH 7.4). Lane 5: wash 2 (40 mM imidazole in 1X PBS pH 7.4). Lane 6: elution in 500 mM imidazole in 1X PBS pH 7.4. Lane 7: after size exclusion. Lane 8: ladder.

5.1.5 Gel of G65C purification

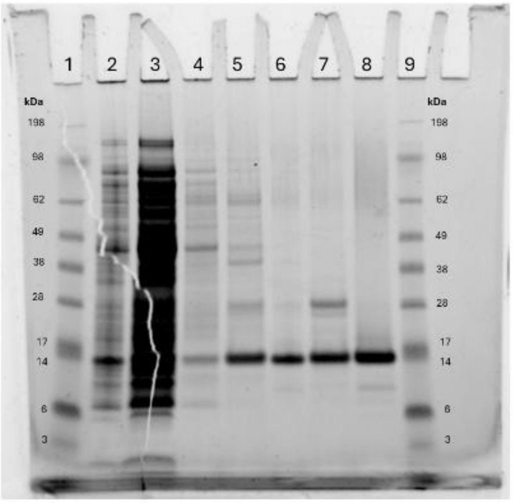

Figure S43: InstantBlue-stained NuPAGE™ gel of G65C purification. Lane 1: ladder. Lane 2: cleared lysate. Lane 3: flow through. Lane 4: wash 1 (20 mM imidazole in 1X PBS pH 7.4). Lane 5: wash 2 (40 mM imidazole in 1X PBS pH 7.4). Lane 6: elution in 500 mM imidazole in 1X PBS pH 7.4. Lane 7: size exclusion peak 1 (dimer). Lane 8: size exclusion peak 2. Lane 9: ladder.

5.2 SDS-PAGE characterization of protein-RNA bioconjugates

5.2.1 Gel of BC06 (I61C-L1-RNA)

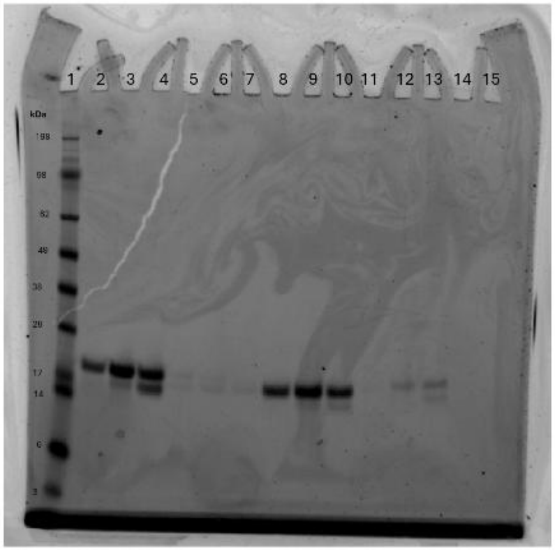

Figure S44: InstantBlue-stained NuPAGE™ gel of **BC06** (I61C-L1-RNA) purification. Lane 1: ladder. Lane 2: SEC fraction 9. Lane 3: SEC fraction 10. Lane 4: SEC fraction 11. Lane 5–13: unrelated samples for a separate experiment. Lane 14-15: empty.

5.2.2 Gel of BC08 (I61C-L2-RNA)

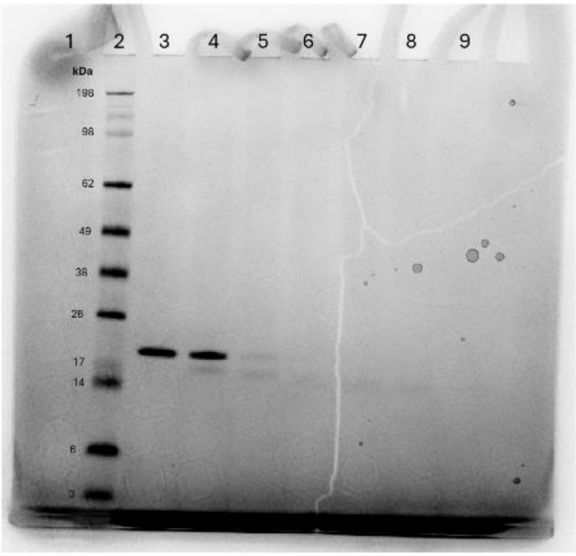

Figure S45: InstantBlue-stained NuPAGE™ gel of **BC08** (I61C-L2-RNA) purification. Lane 1: empty. Lane 2: ladder. Lane 3: SEC fraction B.4. Lane 4: SEC fraction B.5. Lane 5: SEC fraction B.6. Lane 6: SEC fraction B.7. Lane 7: SEC fraction B.8. Lane 8: SEC fraction C.1. Lane 9: SEC fraction C.2.

5.2.3 Gel of BC10 (E62C-L1-RNA)

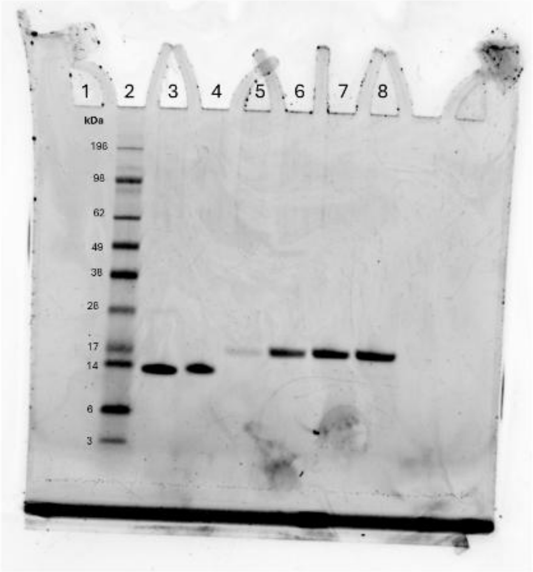

Figure S46: InstantBlue-stained NuPAGE™ gel of **BC10** (E62C-L1-RNA) purification. Lane 1: empty. Lane 2: ladder. Lane 3: E62C. Lane 4: E62C-L1. Lane 5: SEC fraction 16. Lane 6: SEC fraction 17. Lane 7: SEC fraction 18. Lane 8: SEC fraction 19.

5.2.4 Gel of BC12 (E62C-L2-RNA)

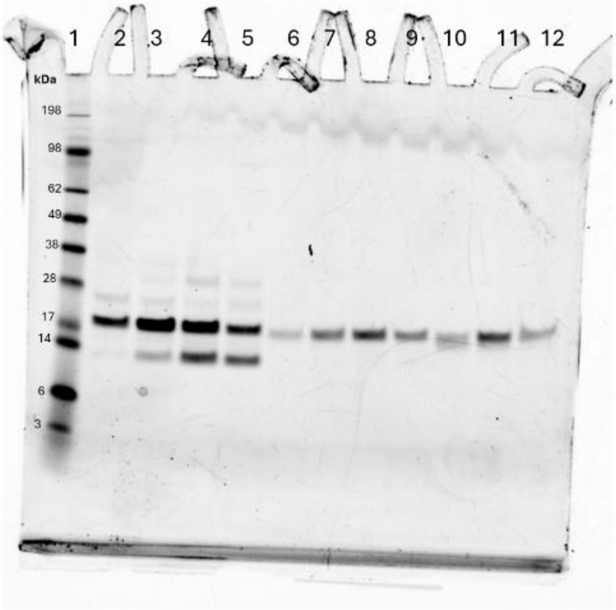

Figure S47: InstantBlue-stained NuPAGE™ gel of **BC12** (E62C-L2-RNA) purification. Lane 1: ladder. Lanes 2–5: unrelated samples from a separate experiment. Lane 6: SEC fraction B.1. Lane 7: SEC fraction B.2. Lane 8: SEC fraction B.3. Lane 9: SEC fraction B.4. Lanes 10–12: unrelated samples from a separate experiment.

5.2.5 Gel of BC02 (E60C-L1-RNA), BC04 (E60C-L2-RNA), BC14 (G65C-L1-RNA), and BC16 (G65C-L2-RNA)

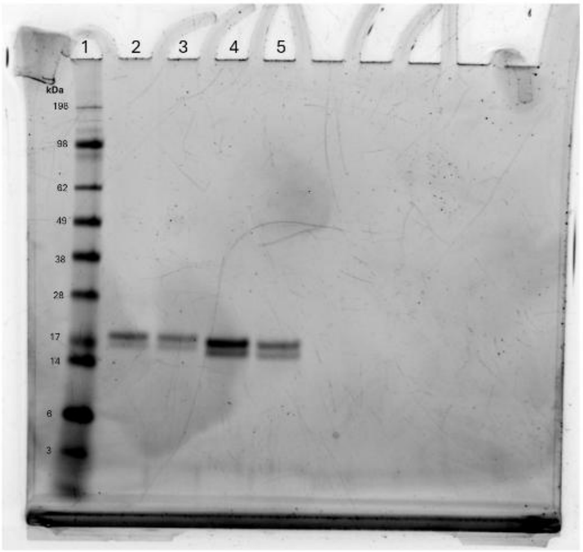

Figure S48: InstantBlue-stained NuPAGE™ gel of **BC02** (E60C-L1-RNA), **BC04** (E60C-L2-RNA), **BC14** (G65C-L1-RNA), and **BC16** (G65C-L2-RNA) purification. Lane 1: ladder. Lane 2: **BC16** pooled SEC fractions B.1–B.3. Lane 3: **BC14** pooled SEC fractions B.1–B.4. Lane 4: **BC04** pooled SEC fractions B.1–B.5. Lane 5: **BC02** pooled SEC fractions B.3–B.5.

5.2.6 Gel of BC18 (BH-E62C-L1-RNA)

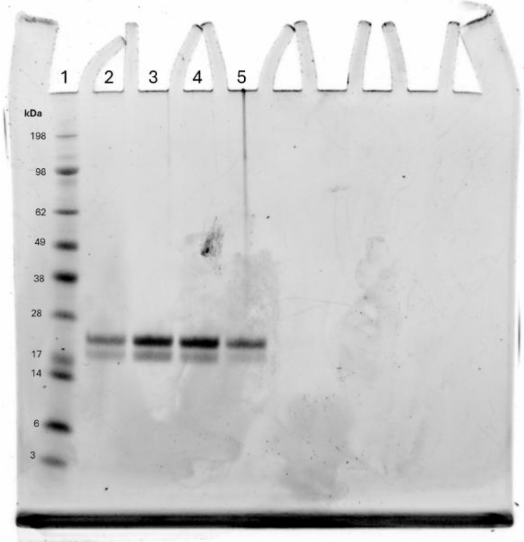

Figure S49: InstantBlue-stained NuPAGE™ gel of **BC18** (BH-E62C-L1-RNA). Lane 1: ladder. Lane 2: SEC fraction 15. Lane 3: SEC fraction 16. Lane 4: SEC fraction 17. Lane 5: SEC fraction 18.

5.3 SDS-PAGE characterization of Cas-13 degradation of BC10

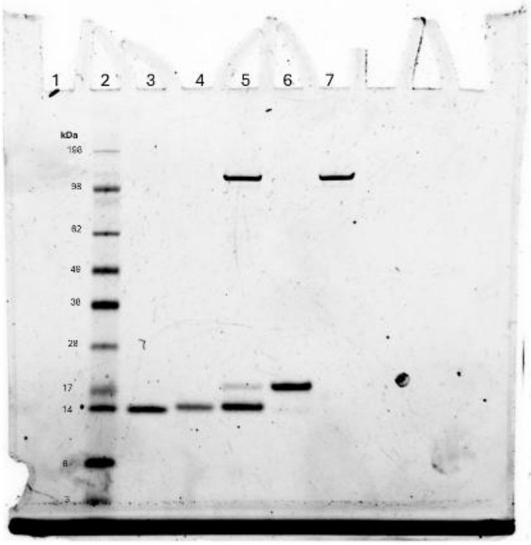

Figure S50: InstantBlue-stained NuPAGE™ gel of **BC10** (E62C-L1-RNA) treated with Cas13. Lane 1: empty. Lane 2: ladder. Lane 3: E62C. Lane 4: **BC09** (E62C-L1). Lane 5: **BC10** treated with Cas13. Lane 6: **BC10** untreated. Lane 7: Cas13 only.

5.4 RNA denaturing PAGE

5.4.1 Viral transcripts

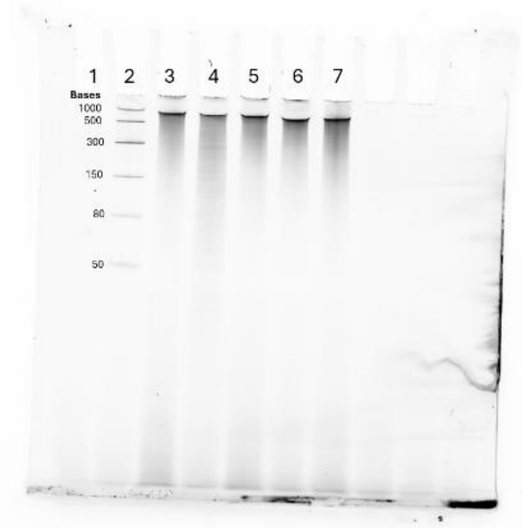

Figure S51: 15% TBE-Urea gel stained with SYBR Gold following T7 transcription and purification of viral transcripts. Lane 1: empty. Lane 2: ladder. Lane 3: H1N1 NP transcript. Lane 4: H3N2 NP transcript. Lane 5: COVID NP transcript. Lane 6: hCoV-229E NP transcript. Lane 7: RSV NP transcript.

5.4.2 crRNA efficacy screen

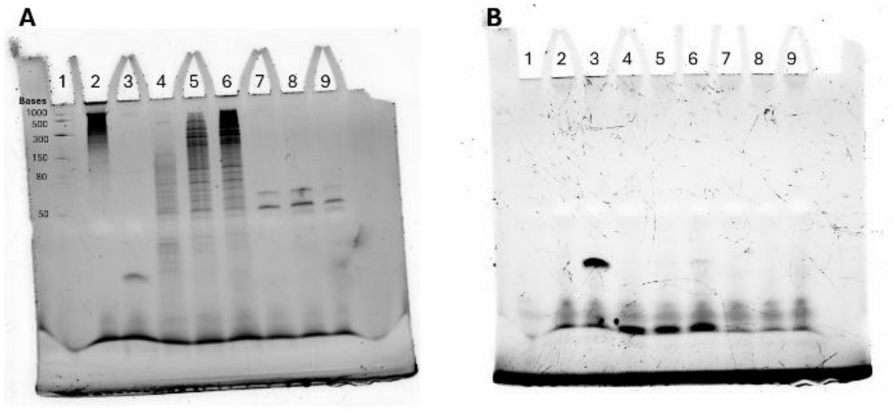

Figure S52: 15% TBE-Urea gel stained with SYBR Gold (**A**) and imaged for fluorescein (**B**) with different crRNAs. Lane 1: Ladder. Lane 2: Target RNA only. Lane 3: **conjRNA2**. Lane 4: Cas13 + **crRNA1** + target + **conjRNA2**. Lane 5: Cas13 + **crRNA2** + target + **conjRNA2**. Lane 6: Cas13 + **crRNA3** + target + **conjRNA2**. Lane 7: Cas13 + **crRNA1**. Lane 8: Cas13 + **crRNA2**. Lane 9: Cas13 + **crRNA3**.

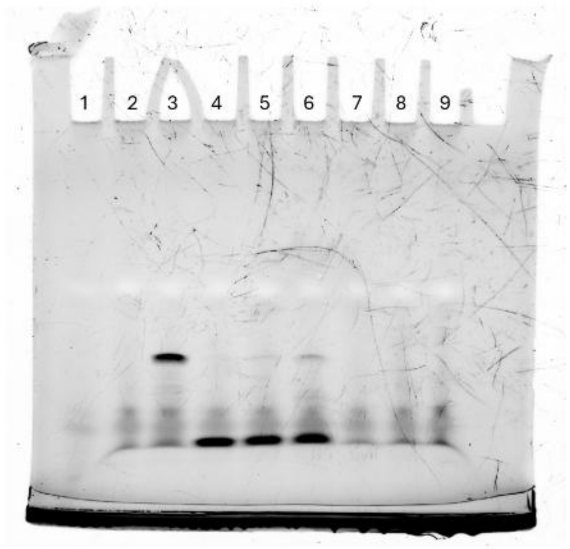

Figure S53: 15% TBE-Urea gel imaged for fluorescein with different crRNAs replicate. Lane 1: Ladder (not visible in this channel). Lane 2: Target RNA only. Lane 3: **conjRNA2**. Lane 4: Cas13 + **crRNA1** + target + **conjRNA2**. Lane 5: Cas13 + **crRNA2** + target + **conjRNA2**. Lane 6: Cas13 + **crRNA3** + target + **conjRNA2**. Lane 7: Cas13 + **crRNA1**. Lane 8: Cas13 + **crRNA2**. Lane 9: Cas13 + **crRNA3**.

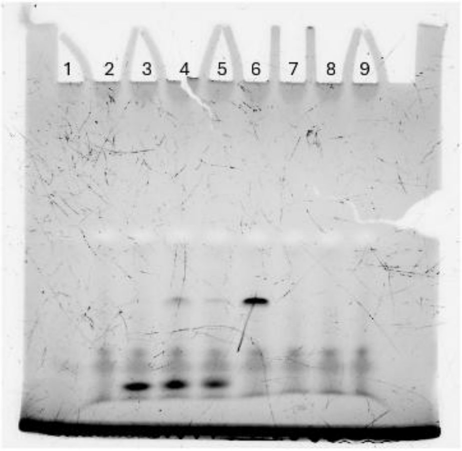

Figure S54: 15% TBE-Urea gel imaged for fluorescein with different crRNAs replicate. Lane 1: Ladder (not visible in this channel). Lane 2: Target RNA only. Lane 3: Cas13 + **crRNA1** + target + **conjRNA2**. Lane 4: Cas13 + **crRNA2** + target + **conjRNA2**. Lane 5: Cas13 + **crRNA3** + target + **conjRNA2**. Lane 6: **conjRNA2**. Lane 7: Cas13 + **crRNA1**. Lane 8: Cas13 + **crRNA2**. Lane 9: Cas13 + **crRNA3**.

5.4.3 Cas13 time trial

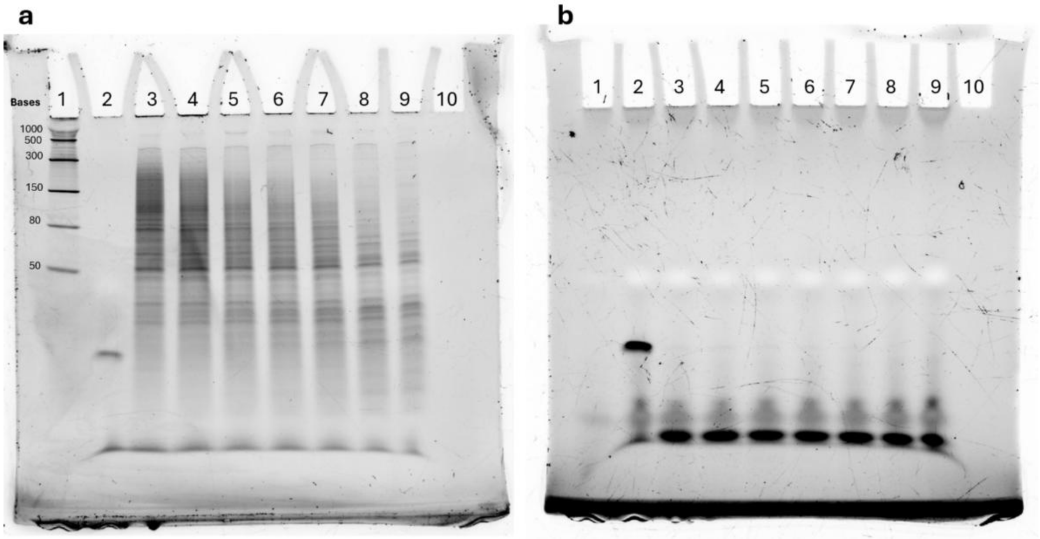

Figure S55: 15% TBE-Urea gel stained with SYBR Gold (**A**) and imaged for fluorescein (**B**) with different treatment times with Cas13. Lane 1: Ladder. Lane 2: **conjRNA2** only. Lane 3: 1 min. Lane 4: 2 mins. Lane 5: 3 mins. Lane 6: 4 mins. Lane 7: 5 mins. Lane 8: 10 mins. Lane 9: 15 mins. Lane 10: empty.

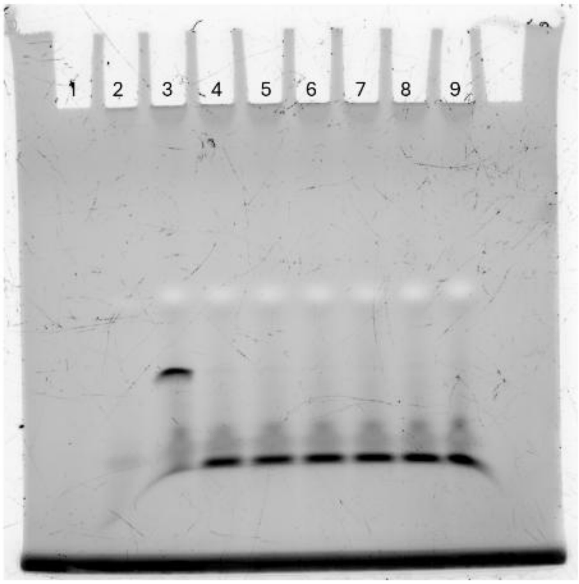

Figure S56: 15% TBE-Urea gel stained imaged for fluorescein with different times of Cas13 activation replicate. Lane 1: Empty. Lane 2: Ladder (not visible in this channel). Lane 3: **conjRNA2**. Lane 4: 1 min. Lane 5: 2 mins. Lane 6: 3 mins. Lane 7: 4 mins. Lane 8: 5 mins. Lane 9: 10 mins.

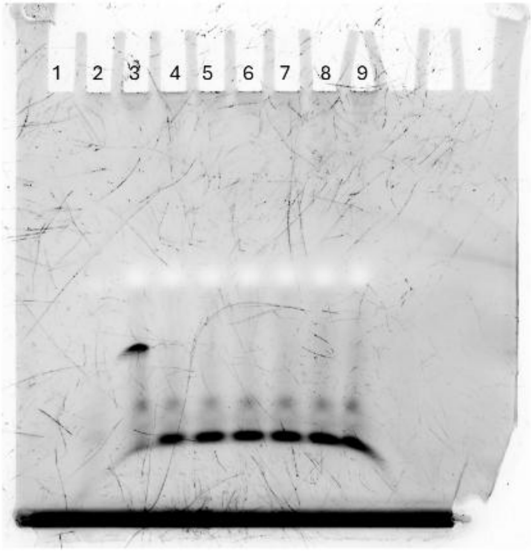

Figure S57: 15% TBE-Urea gel stained imaged for fluorescein with different times of Cas13 activation replicate. Lane 1: Empty. Lane 2: Ladder (not visible in this channel). Lane 3: **conjRNA2**. Lane 4: 1 min. Lane 5: 2 mins. Lane 6: 3 mins. Lane 7: 4 mins. Lane 8: 5 mins. Lane 9: 10 mins.

6. Luminescence Data

6.1 Luminescence data from pure, unmodified proteins

6.1.1 E60C raw signal

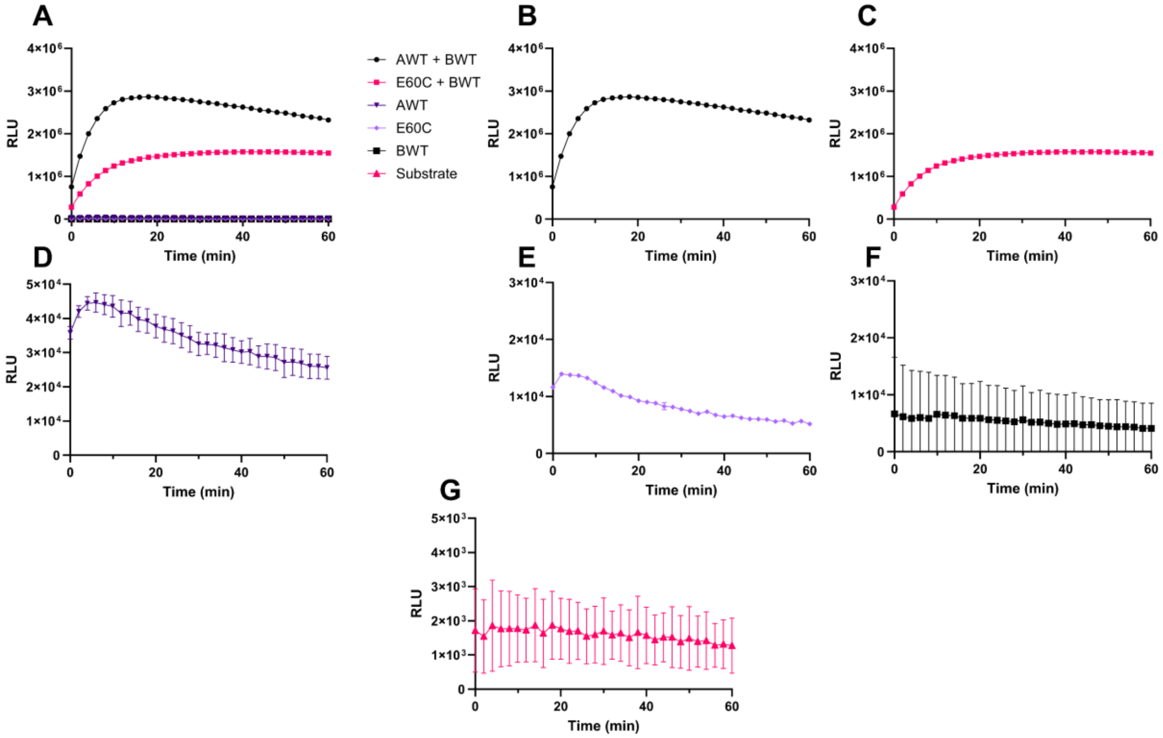

Figure S58: Raw luminescence data with controls for E60C. N = 3 for all sets. (**A**) Combined data set. (**B**) AWT + BWT. (**C**) E60C + BWT. (**D**) AWT. (**E**) E60C. (**F**) BWT. (**G**) Substrate.

6.1.2 I61C, E62C, and G65C raw signal

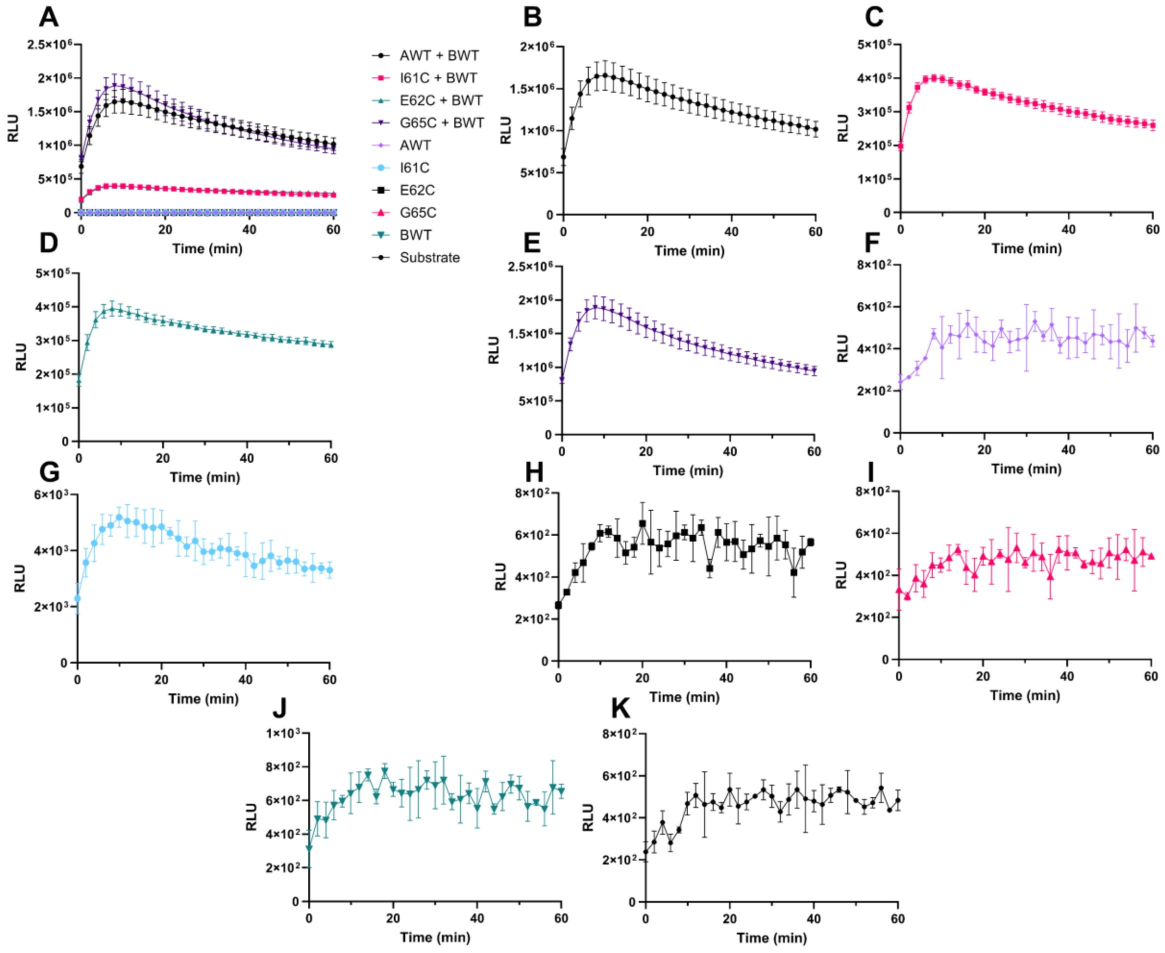

Figure S59: Raw luminescence data with controls for I61C, E62C, and G65C. N = 3 for all sets. (**A**) Combined data set. (**B**) AWT + BWT. (**C**) I61C + BWT. (**D**) E62C + BWT. (**E**) G65C + BWT. (**F**) AWT. (**G**) I61C. (**H**) E62C. (**I**) G65C. (**J**) BWT. (**K**) Substrate.

6.1.3 Mutant signals normalized to wild type

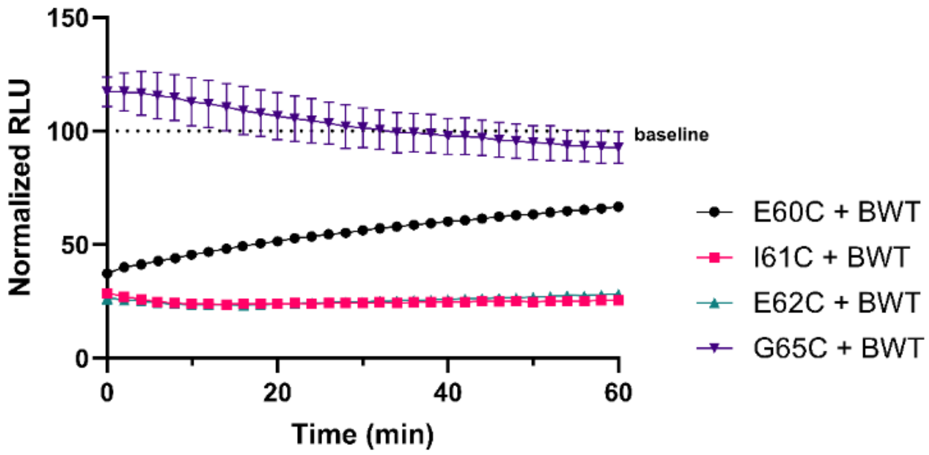

Figure S60: Normalized luminescence data for E60C, I61C, E62C, and G65C. N = 3 for all sets. All cysteine mutants were normalized to the AWT + BWT signal (indicated here as baseline) conducted in parallel with each cysteine mutant.

6.1.4 BH-E62C raw signal

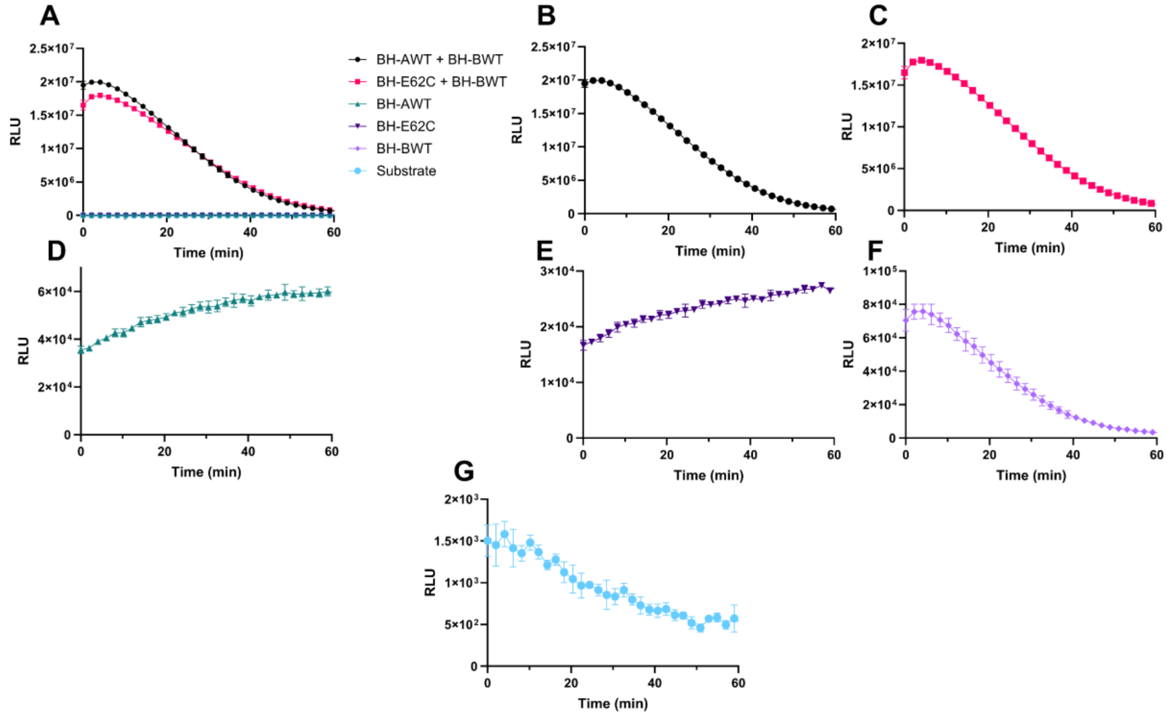

Figure S61: Raw luminescence data with controls for BH-E62C. N = 3 for all sets. (**A**) Combined data set. (**B**) BH-AWT + BH-BWT. (**C**) BH-E62C + BH-BWT. (**D**) BH-AWT. (**E**) BH-E62C. (**F**) BH-BWT. (**G**) Substrate.

6.1.5 BH-E62C signal normalized to wild type

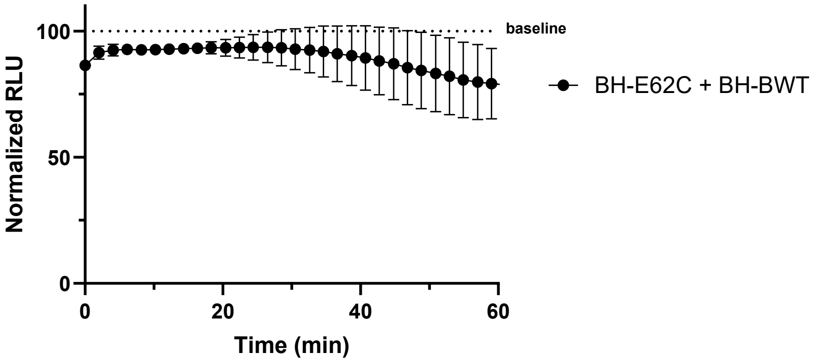

Figure S62: Normalized luminescence data for BH-E62C. The cysteine mutant was normalized to the BH-AWT + BH-BWT signal (indicated here as baseline) conducted in parallel. N = 3.

6.2 Luminescence data from RNase A screen

6.2.1 BC02 raw luminescence with RNase

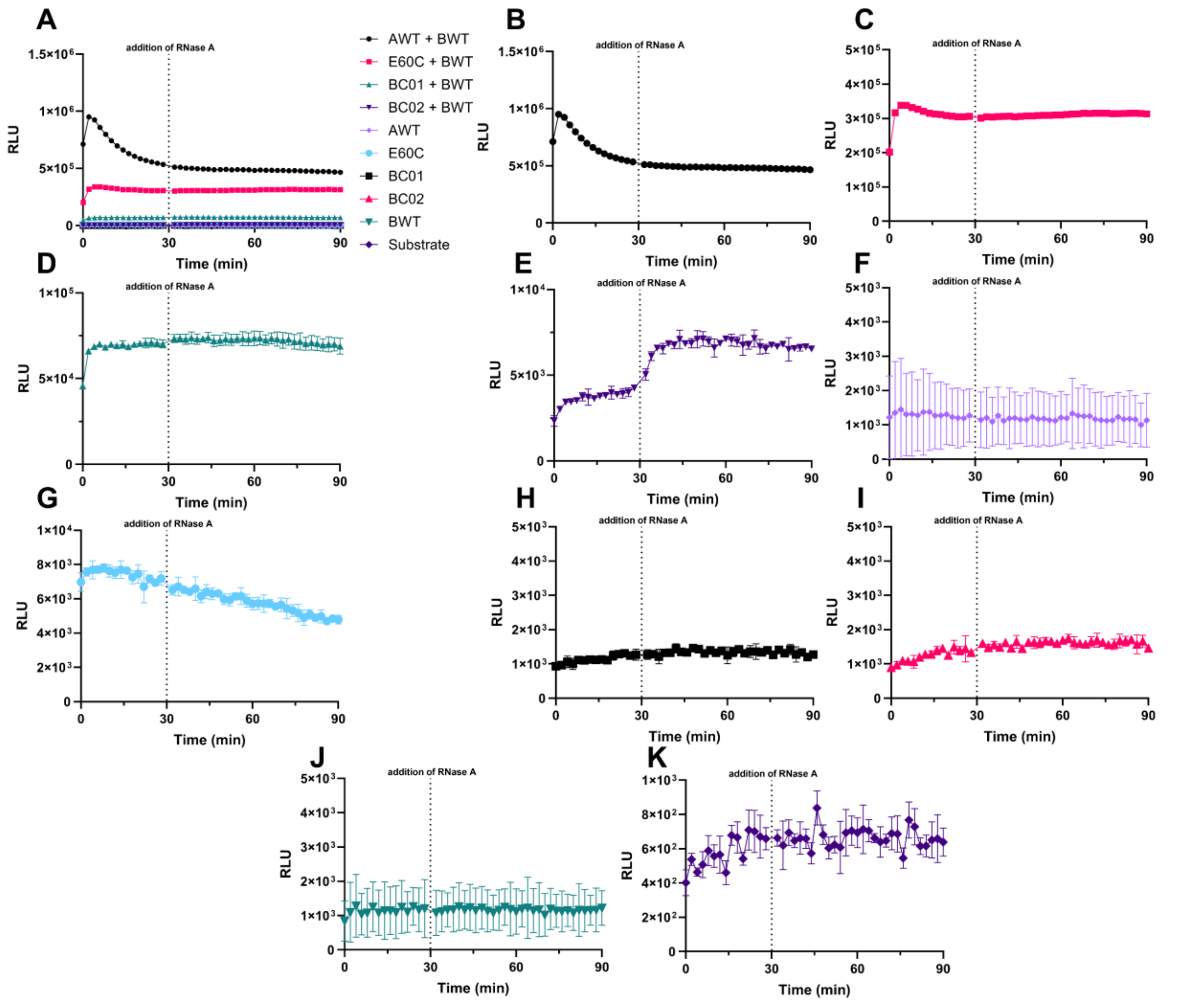

Figure S63: Raw luminescence data with controls for **BC02** with the addition of RNase A. N = 3 for all sets. (**A**) Combined data set. (**B**) AWT + BWT. (**C**) E60C + BWT. (**D**) **BC01** + BWT. (**E**) **BC02** + BWT. (**F**) AWT. (**G**) E60C. (**H**) **BC01**. (**I**) **BC02**. (**J**) BWT. (**K**) Substrate.

6.2.2 BC04 raw luminescence with RNase

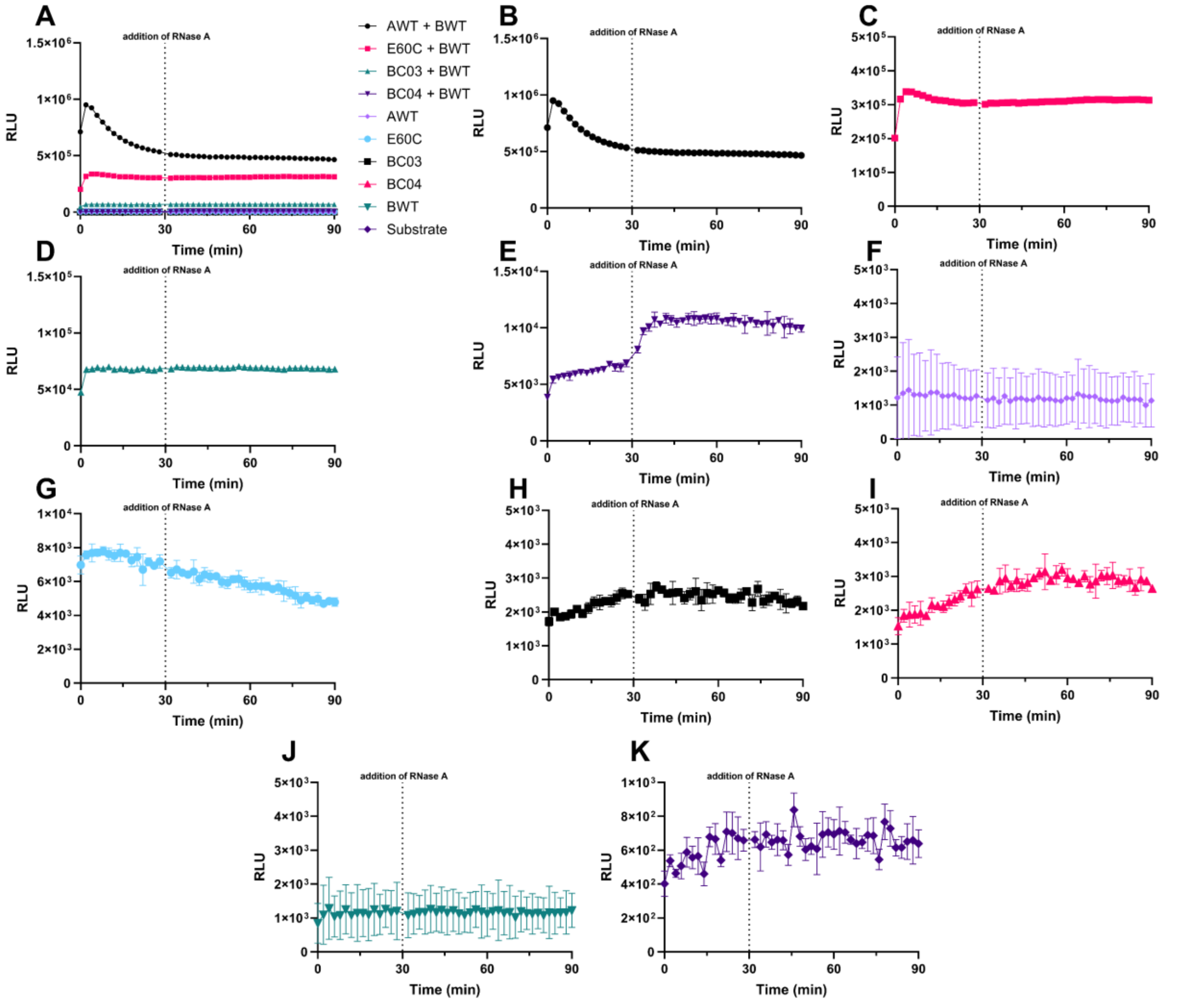

Figure S64: Raw luminescence data with controls for **BC04** with the addition of RNase A. N = 3 for all sets. (**A**) Combined data set. (**B**) AWT + BWT. (**C**) E60C + BWT. (**D**) **BC03** + BWT. (**E**) **BC04** + BWT. (**F**) AWT. (**G**) E60C. (**H**) **BC03**. (**I**) **BC04**. (**J**) BWT. (**K**) Substrate.

6.2.3 BC06 raw luminescence with RNase

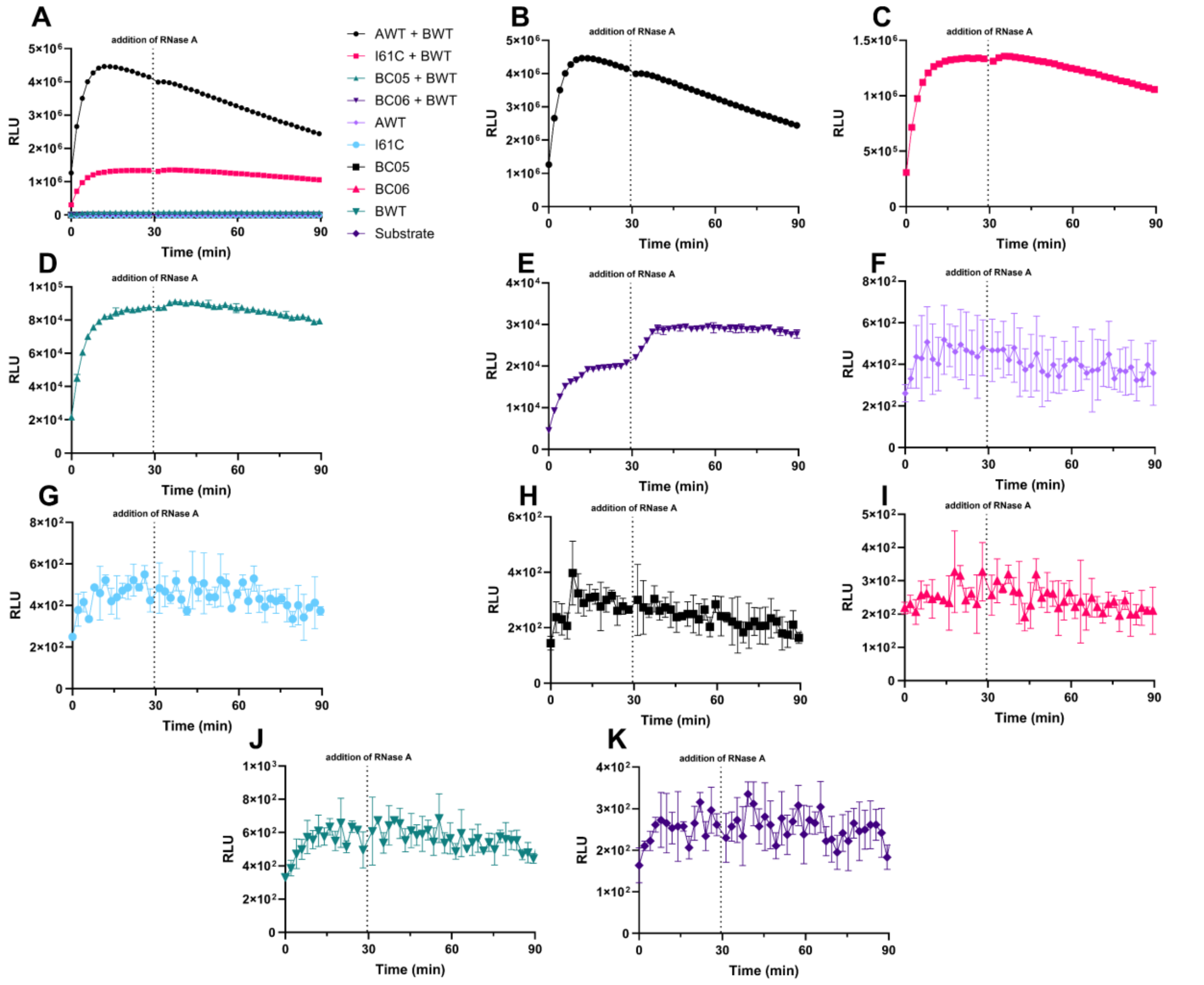

Figure S65: Raw luminescence data with controls for **BC06** with the addition of RNase A. N = 3 for all sets. (**A**) Combined data set. (**B**) AWT + BWT. (**C**) I61C + BWT. (**D**) **BC05** + BWT. (**E**) **BC06** + BWT. (**F**) AWT. (**G**) I61C. (**H**) **BC05**. (**I**) **BC06**. (**J**) BWT. (**K**) Substrate.

6.2.4 BC08 raw luminescence with RNase

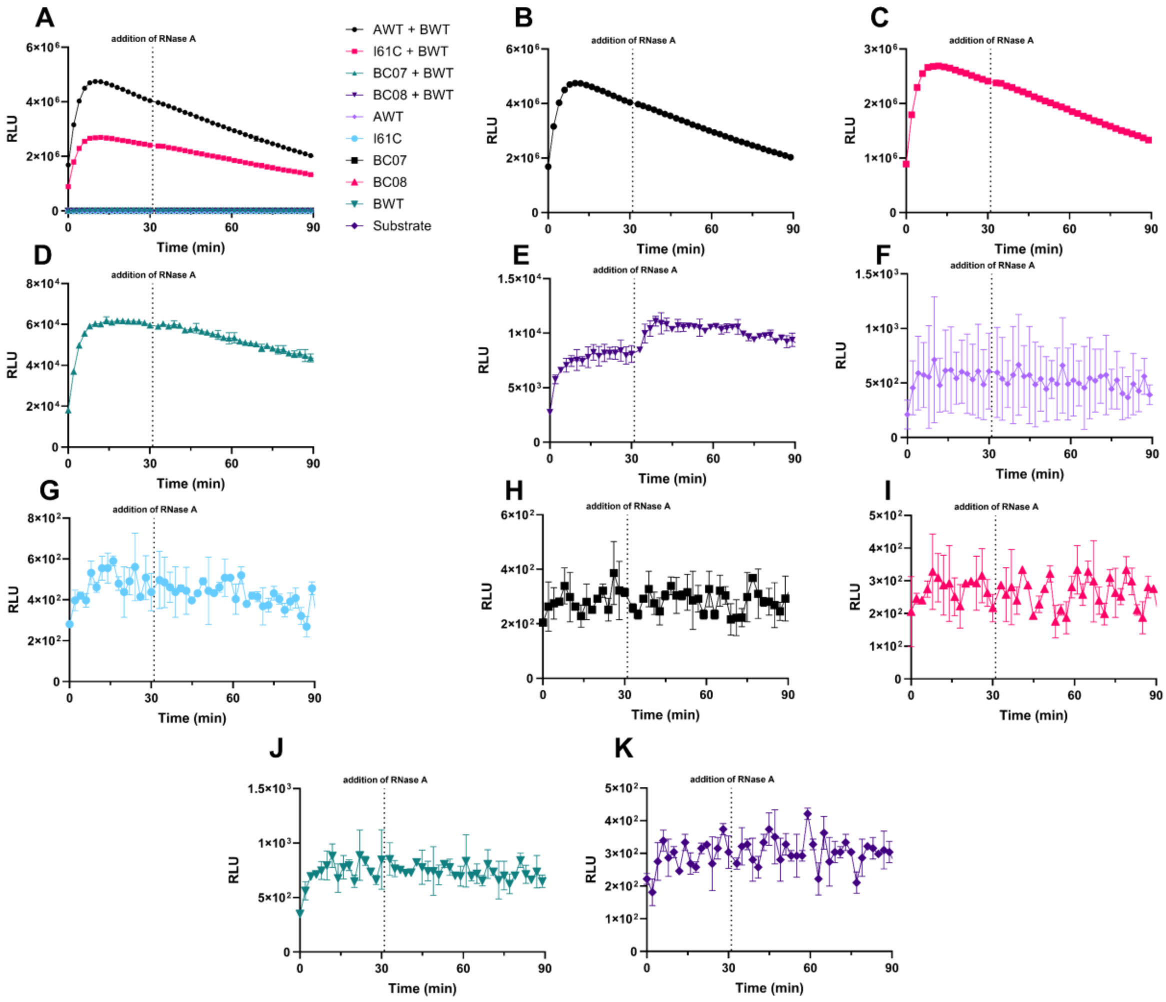

Figure S66: Raw luminescence data with controls for **BC08** with the addition of RNase A. N = 2 for all sets. (**A**) Combined data set. (**B**) AWT + BWT. (**C**) I61C + BWT. (**D**) **BC07** + BWT. (**E**) **BC08** + BWT. (**F**) AWT. (**G**) I61C. (**H**) **BC07**. (**I**) **BC08**. (**J**) BWT. (**K**) Substrate.

6.2.5 BC10 raw luminescence with RNase

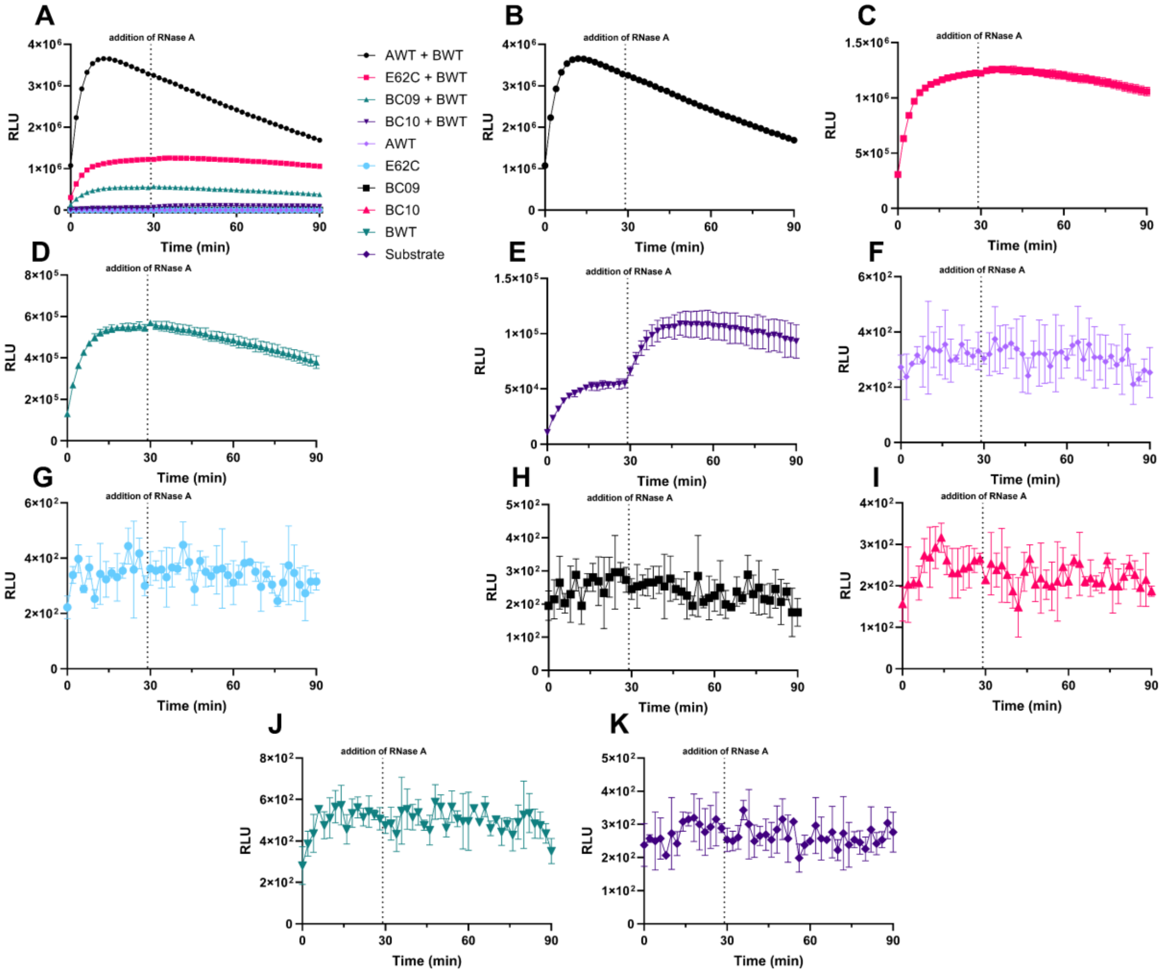

Figure S67: Raw luminescence data with controls for **BC10** with the addition of RNase A. N = 3 for all sets. (**A**) Combined data set. (**B**) AWT + BWT. (**C**) E62C + BWT. (**D**) **BC09** + BWT. (**E**) **BC10** + BWT. (**F**) AWT. (**G**) E62C. (**H**) **BC09**. (**I**) **BC10**. (**J**) BWT. (**K**) Substrate.

6.2.6 BC12 raw luminescence with RNase

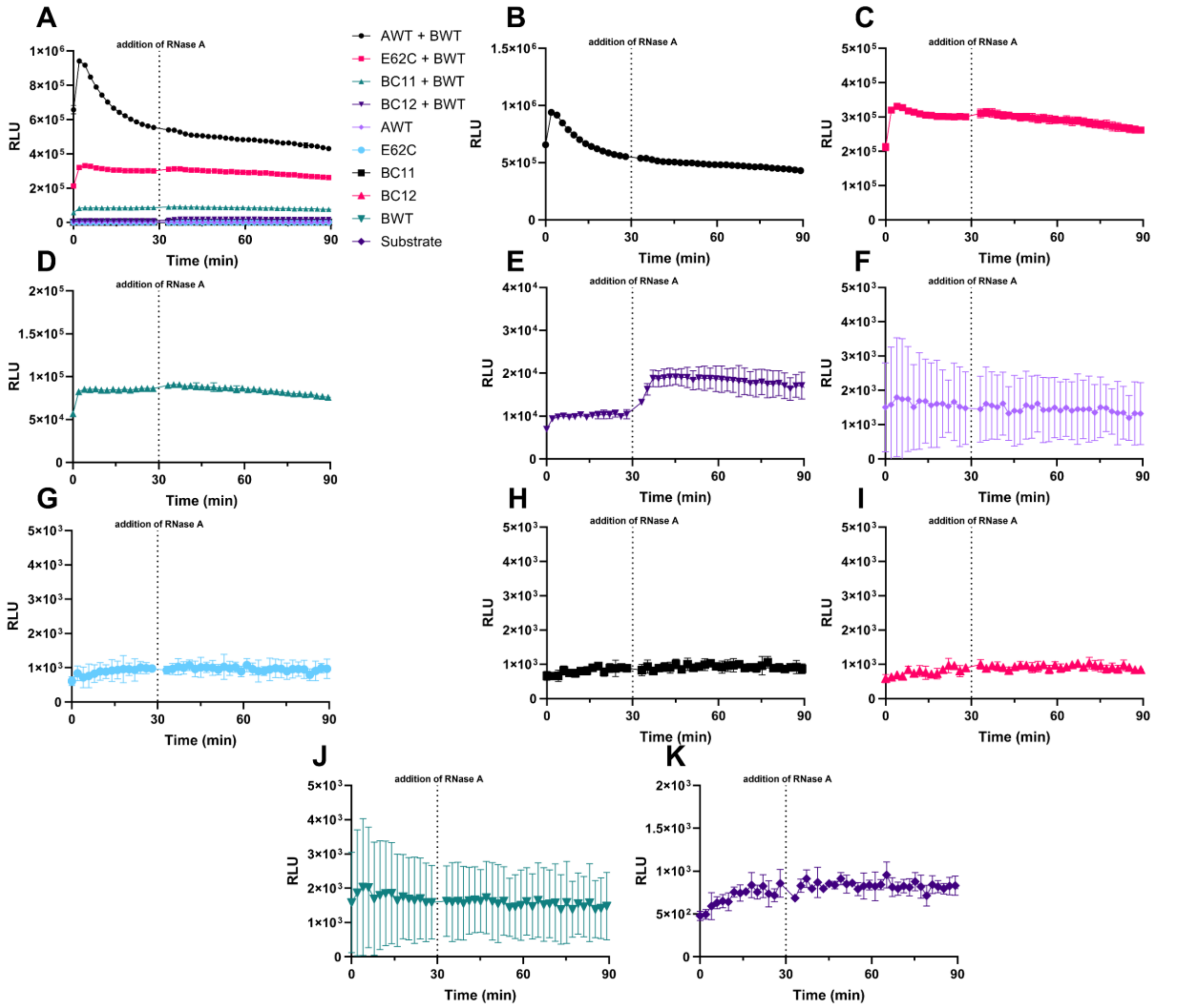

Figure S68: Raw luminescence data with controls for **BC12** with the addition of RNase A. N = 3 for all sets. (**A**) Combined data set. (**B**) AWT + BWT. (**C**) E62C + BWT. (**D**) **BC11** + BWT. (**E**) **BC12** + BWT. (**F**) AWT. (**G**) E62C. (**H**) **BC11**. (**I**) **BC12**. (**J**) BWT. (**K**) Substrate.

6.2.7 BC14 raw luminescence with RNase

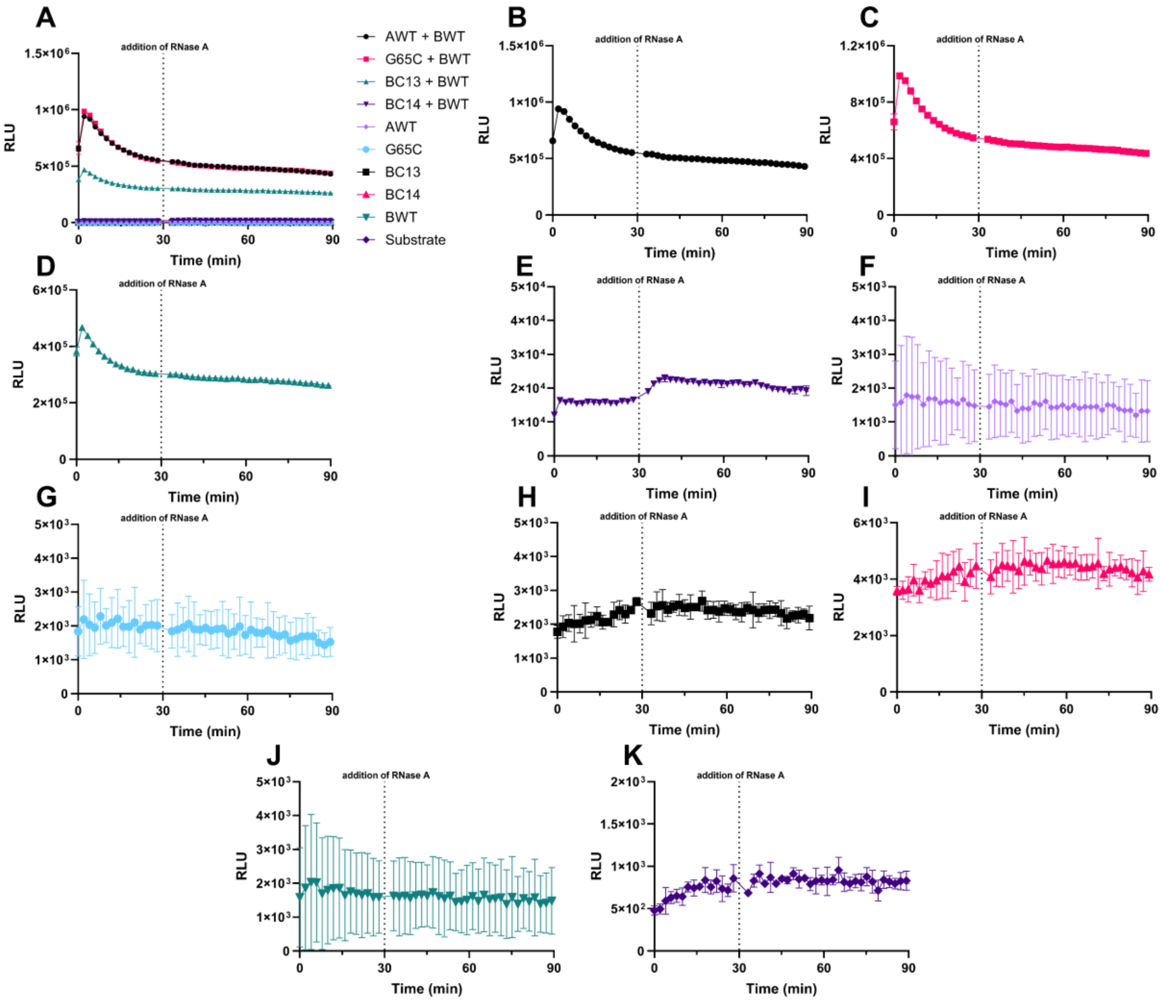

Figure S69: Raw luminescence data with controls for **BC14** with the addition of RNase A. N = 3 for all sets. (**A**) Combined data set. (**B**) AWT + BWT. (**C**) G65C + BWT. (**D**) **BC13** + BWT. (**E**) **BC14** + BWT. (**F**) AWT. (**G**) G65C. (**H**) **BC13**. (**I**) **BC14**. (**J**) BWT. (**K**) Substrate.

6.2.8 BC16 raw luminescence with RNase

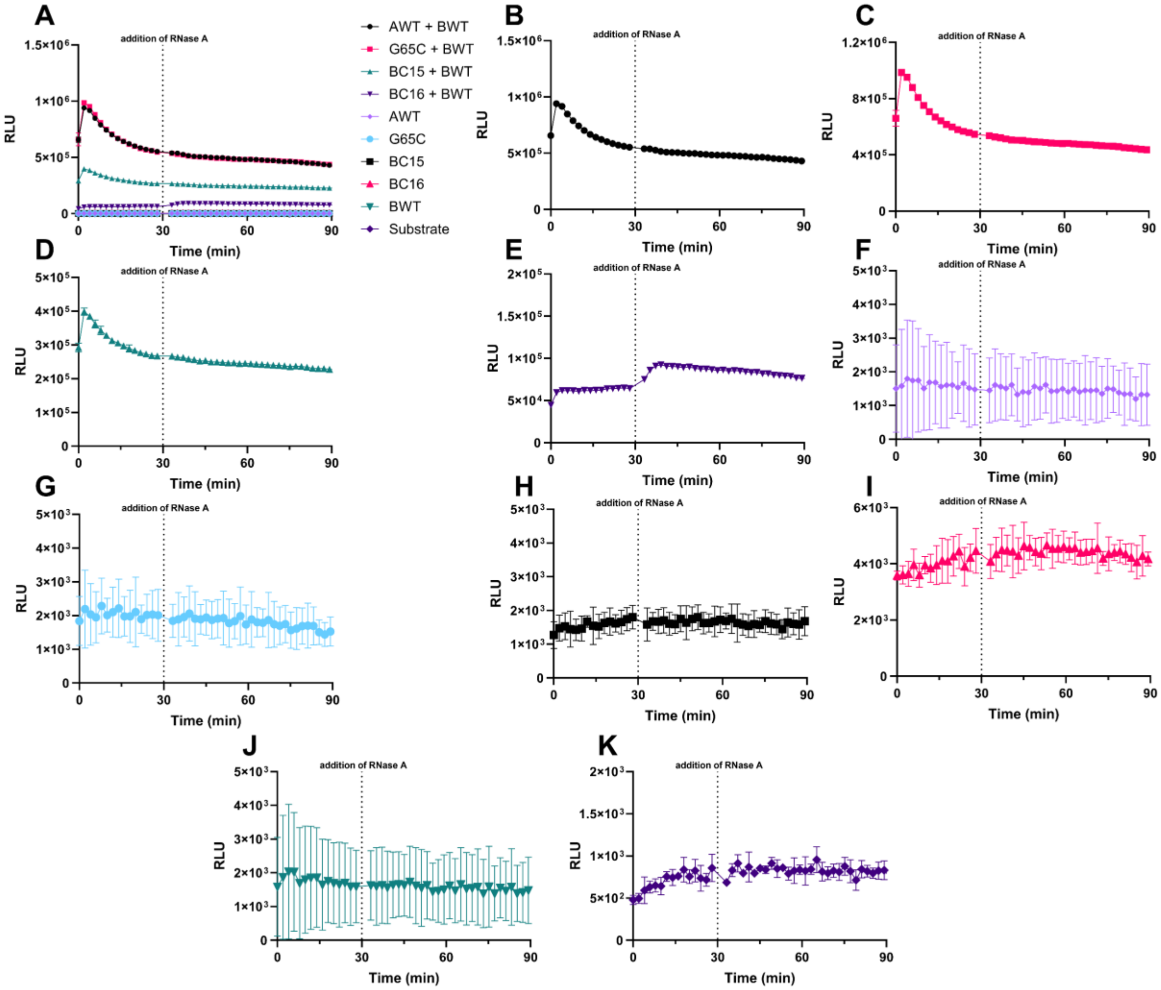

Figure S70: Raw luminescence data with controls for **BC16** with the addition of RNase A. N = 3 for all sets. (**A**) Combined data set. (**B**) AWT + BWT. (**C**) G65C + BWT. (**D**) **BC15** + BWT. (**E**) **BC16** + BWT. (**F**) AWT. (**G**) G65C. (**H**) **BC15**. (**I**) **BC16**. (**J**) BWT. (**K**) Substrate.

6.2.9 BC18 raw luminescence with RNase

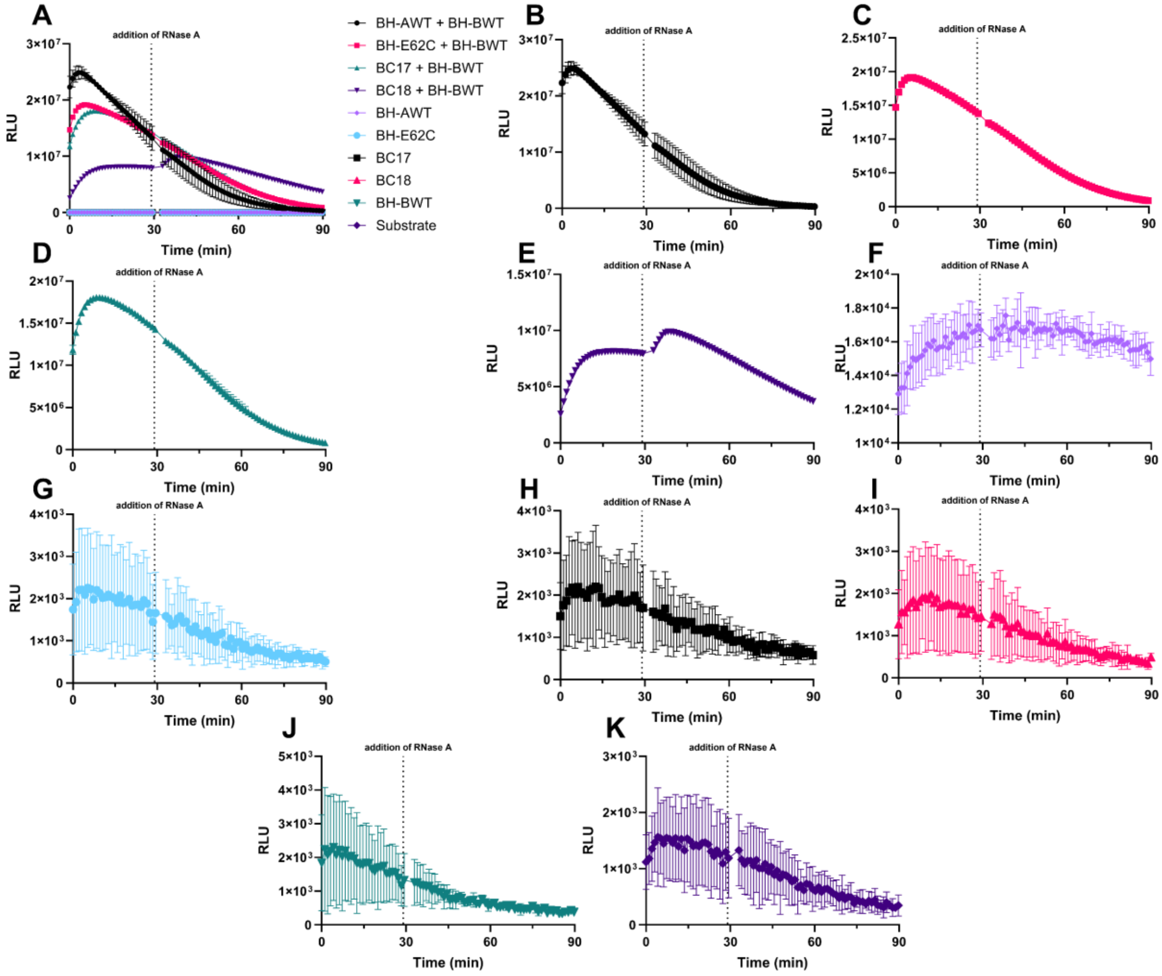

Figure S71: Raw luminescence data with controls for **BC18** with the addition of RNase A. N = 3 for all sets. (**A**) Combined data set. (**B**) BH-AWT + BH-BWT. (**C**) BH-E62C + BH-BWT. (**D**) **BC17** + BH-BWT. (**E**) **BC18** + BH-BWT. (**F**) BH-AWT. (**G**) BH-E62C. (**H**) **BC17**. (**I**) **BC18**. (**J**) BH-BWT. (**K**) Substrate.

6.2.10 Combined analysis of RNase screen

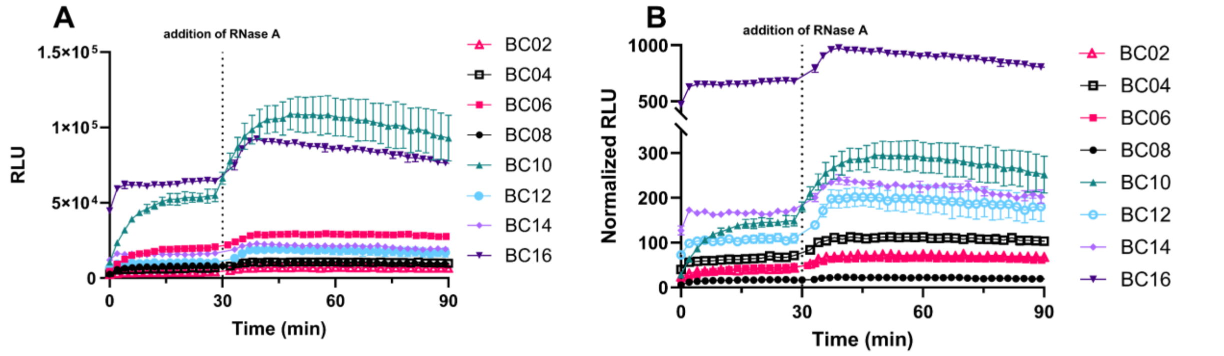

Figure S72: Combined luminescence data for RNase Library. N = 3 for all sets except **BC08** where N = 2. (**A**) Raw signals overlaid. (**B**) All signals normalized to wild-type signals (AWT + BWT) recorded in parallel with each conjugate. Wild type signal is defined as 10,000 Normalized RLU. Note, for samples **BC14** and **BC16**, the enhancement of signal in panel B relative to panel A relates to the inherent brighter luminescence of G65C relative to the other cysteine mutants.

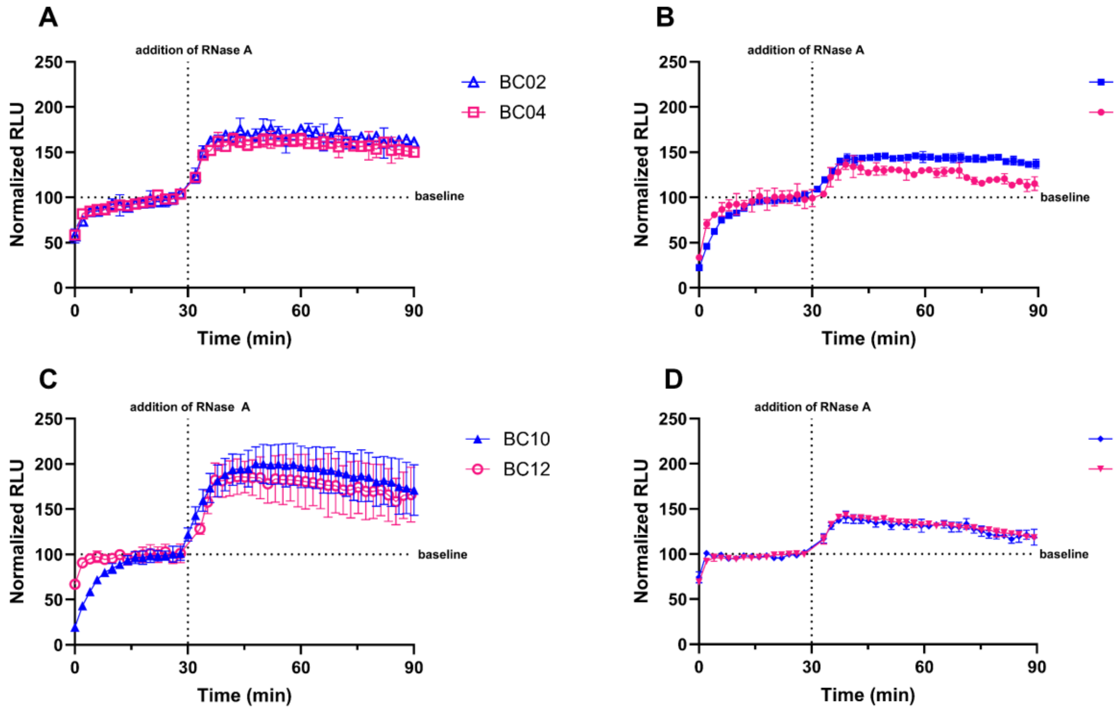

Figure S73: Combined normalized luminescence data by cysteine mutant for RNase Library. N = 3 for all sets except **BC08** where N = 2. All data sets were normalized to the baseline signal. Baseline is defined as the average signal before the addition of RNase A. Blue signals are conjugates made with L1, and pink signals are conjugates made with L2. (**A**) E60C conjugates. (**B**) I61C conjugates. (**C**) E62C conjugates. (**D**) G65C conjugates.

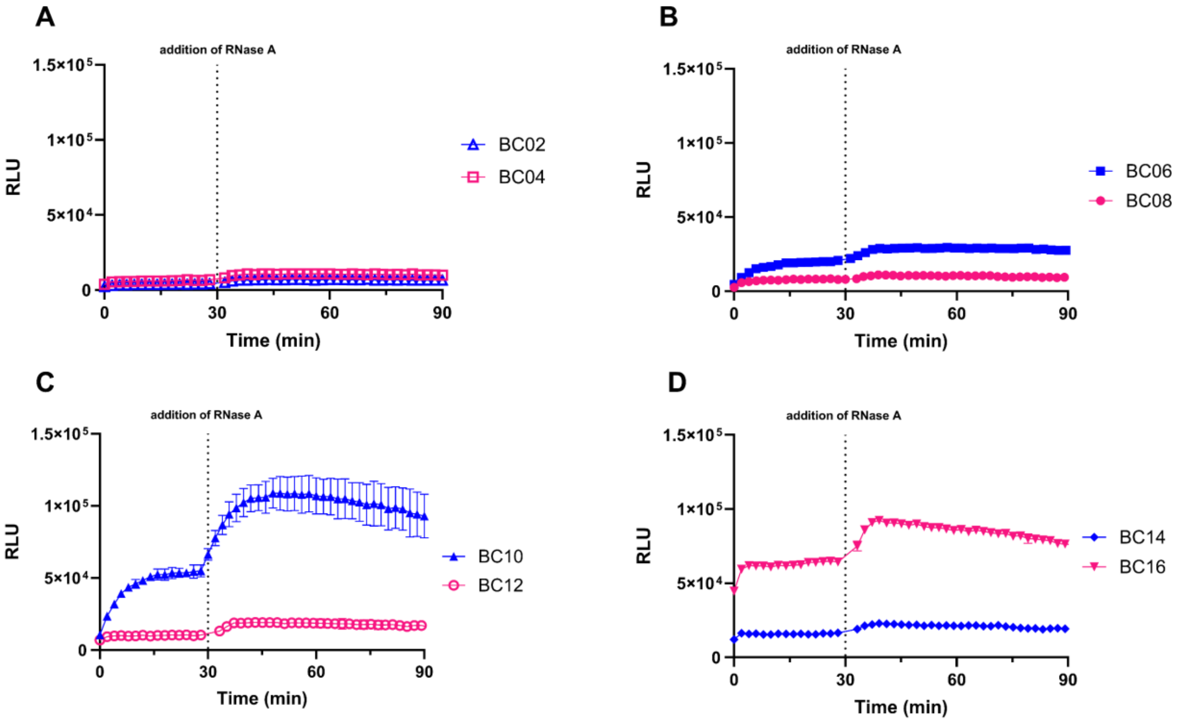

Figure S74: Combined raw luminescence data by cysteine mutant for RNase Library. N = 3 for all sets except **BC08** where N = 2. All data sets were normalized to the wild-type signals (AWT + BWT) recorded at the same time as each sample. Blue signals are conjugates made with **L1**, and pink signals are conjugates made with **L2**. (**A**) E60C conjugates. (**B**) I61C conjugates. (**C**) E62C conjugates. (**D**) G65C conjugates.

6.3 Matrix screen

6.3.1 BC10 matrix raw data

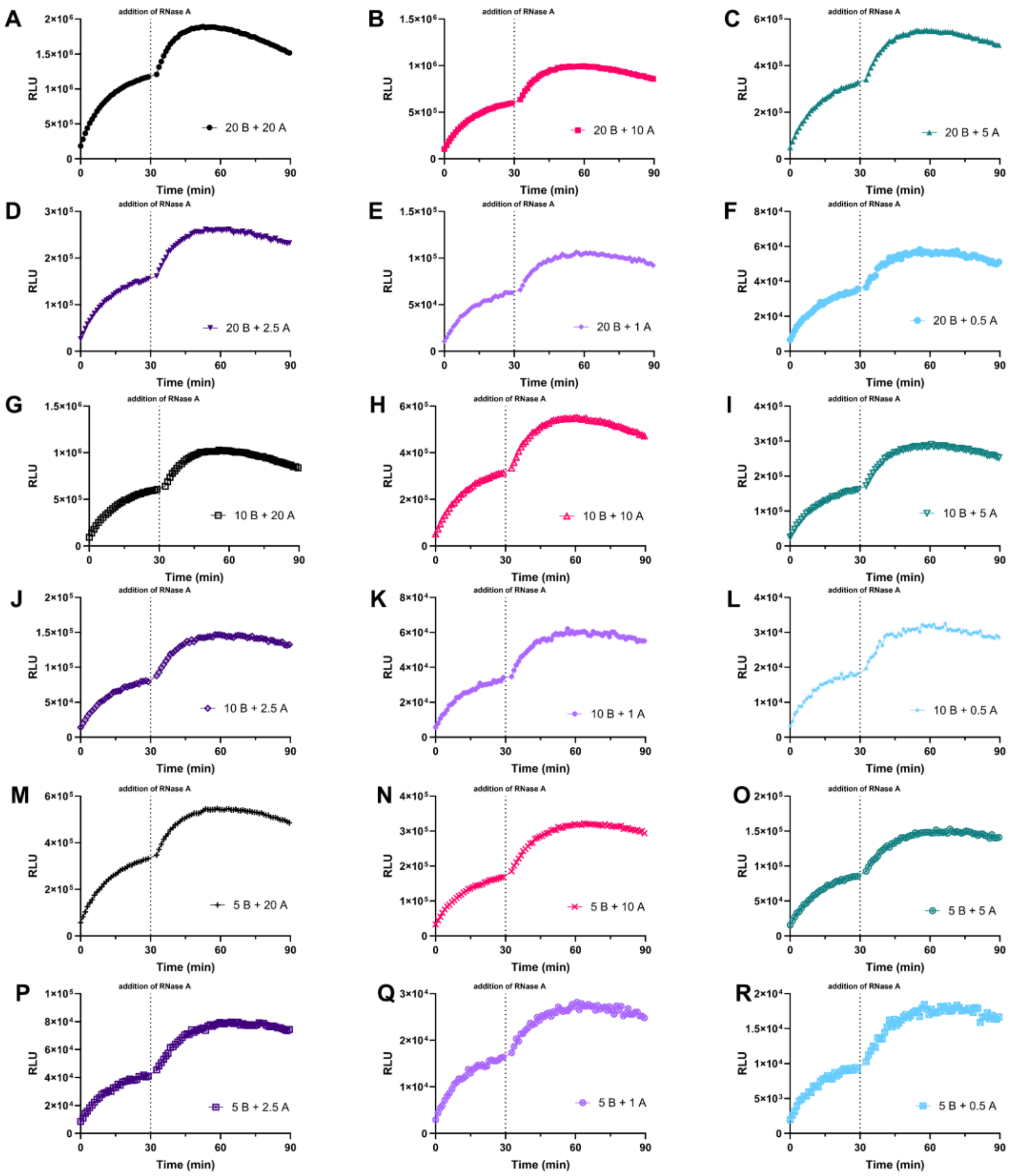

Figure S75: Raw luminescence data for **BC10** treated with RNase A at different concentrations of A protein (**BC10**) and B protein (BWT) part 1. (**A**) 20 nM B + 20 nM A. (**B**) 20 nM B + 10 nM A. (**C**) 20 nM B + 5 nM A. (**D**) 20 nM B + 2.5 nM A. (**E**) 20 nM B + 1 nM A. (**F**) 20 nM B + 0.5 nM A. (**G**) 10 nM B + 20 nM A. (**H**) 10 nM B + 10 nM A. (**I**) 10 nM B + 5 nM A. (**J**) 10 nM B + 2.5 nM A. (**K**) 10 nM B + 1 nM A. (**L**) 10 nM B + 0.5 nM A. (**M**) 5 nM B + 20 nM A. (**N**) 5 nM B + 10 nM A. (**O**) 5 nM B + 5 nM A. (**P**) 5 nM B + 2.5 nM A. (**Q**) 5 nM B + 1 nM A. (**R**) 5 nM B + 0.5 nM A.

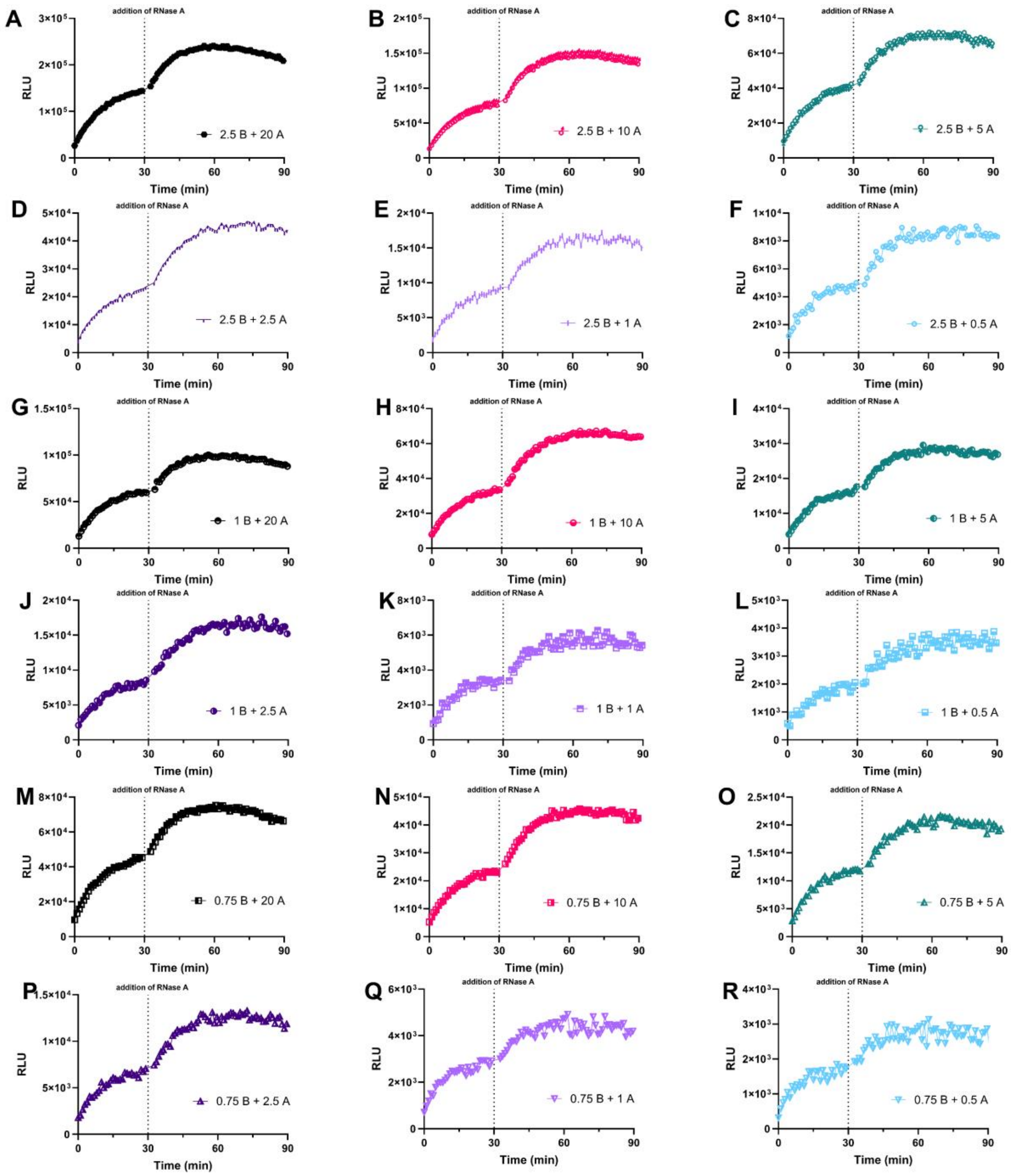

Figure S76: Raw luminescence data for **BC10** treated with RNase A at different concentrations of A protein (**BC10**) and B protein (BWT) part 2. (**A**) 2.5 nM B + 20 nM A. (**B**) 2.5 nM B + 10 nM A. (**C**) 2.5 nM B + 5 nM A. (**D**) 2.5 nM B + 2.5 nM A. (**E**) 2.5 nM B + 1 nM A. (**F**) 2.5 nM B + 0.5 nM A. (**G**) 1 nM B + 20 nM A. (**H**) 1 nM B + 10 nM A. (**I**) 1 nM B + 5 nM A. (**J**) 1 nM B + 2.5 nM A. (**K**) 1 nM B + 1 nM A. (**L**) 1 nM B + 0.5 nM A. (**M**) 0.75 nM B + 20 nM A. (**N**) 0.75 nM B + 10 nM A. (**O**) 0.75 nM B + 5 nM A. (**P**) 0.75 nM B + 2.5 nM A. (**Q**) 0.75 nM B + 1 nM A. (**R**) 0.75 nM B + 0.5 nM A.

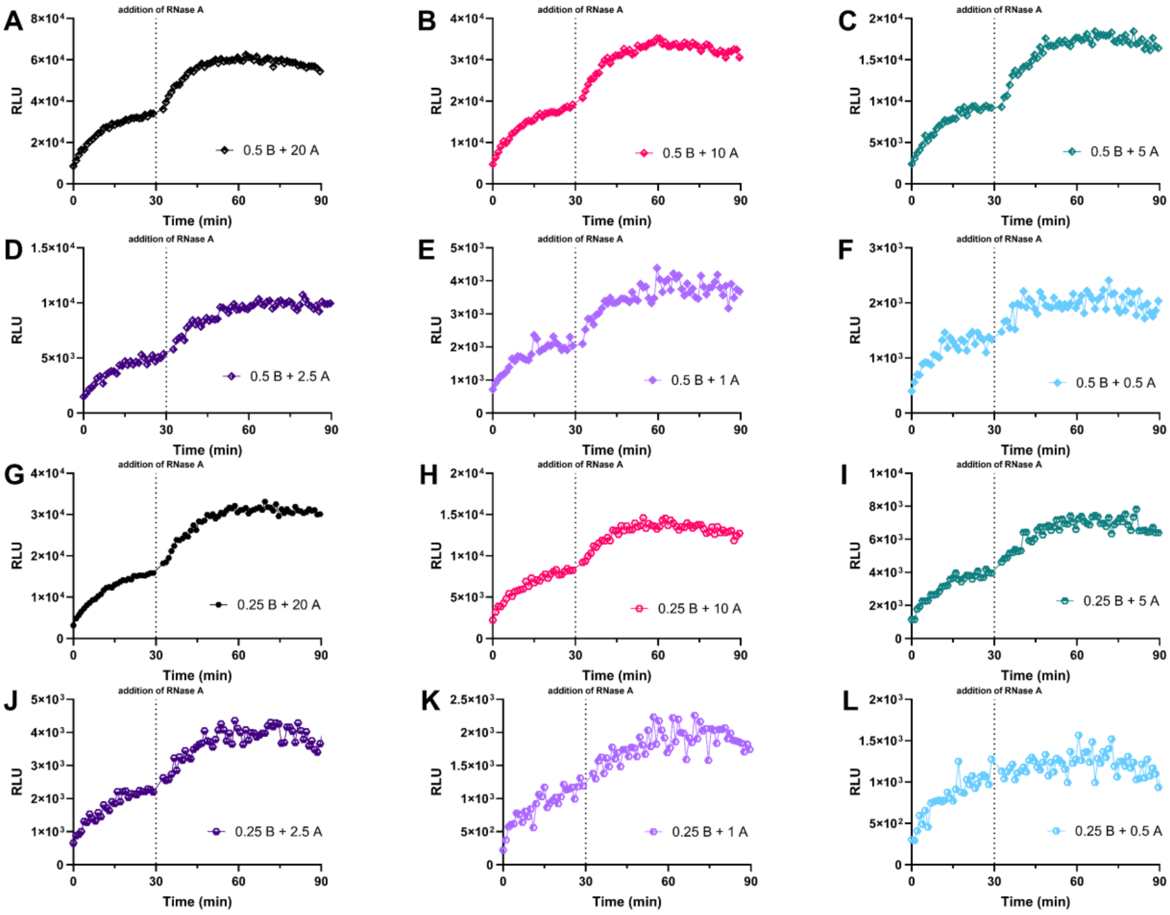

Figure S77: Raw luminescence data for **BC10** treated with RNase A at different concentrations of A protein (**BC10**) and B protein (BWT) part 3. (**A**) 0.5 nM B + 20 nM A. (**B**) 0.5 nM B + 10 nM A. (**C**) 0.5 nM B + 5 nM A. (**D**) 0.5 nM B + 2.5 nM A. (**E**) 0.5 nM B + 1 nM A. (**F**) 0.5 nM B + 0.5 nM A. (**G**) 0.25 nM B + 20 nM A. (**H**) 0.25 nM B + 10 nM A. (**I**) 0.25 nM B + 5 nM A. (**J**) 0.25 nM B + 2.5 nM A. (**K**) 0.25 nM B + 1 nM A. (**L**) 0.25 nM B + 0.5 nM A.

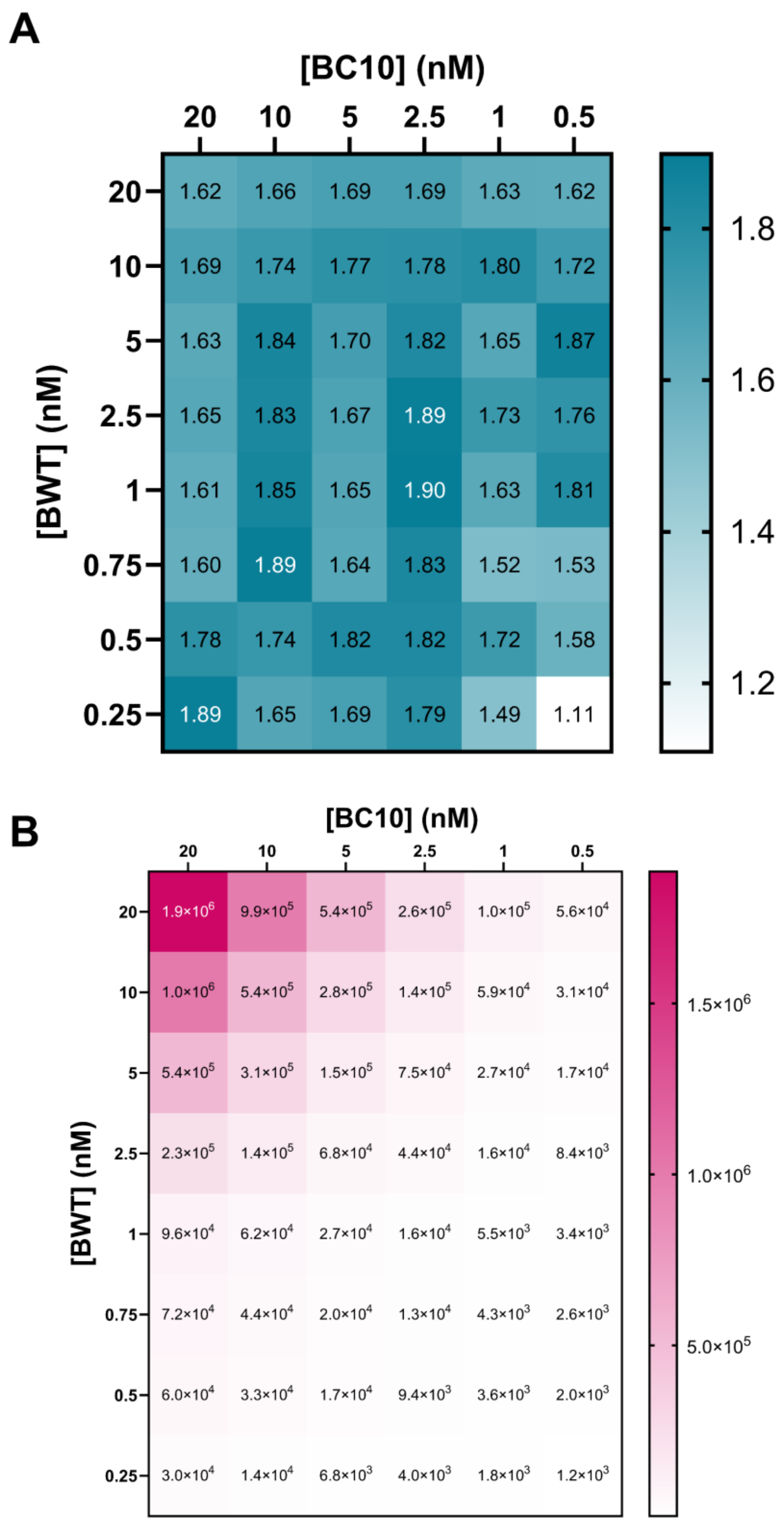

Figure S78: Luminescence data for **BC10** treated with RNase A at different concentrations of A protein (**BC10**) and B protein (BWT). (**A**) Signal-to-baseline (S/B) change after addition of target. Signal measured at baseline and at time = 60 minutes. (**B**) Maximum signal.

6.3.2 BC18 matrix screen

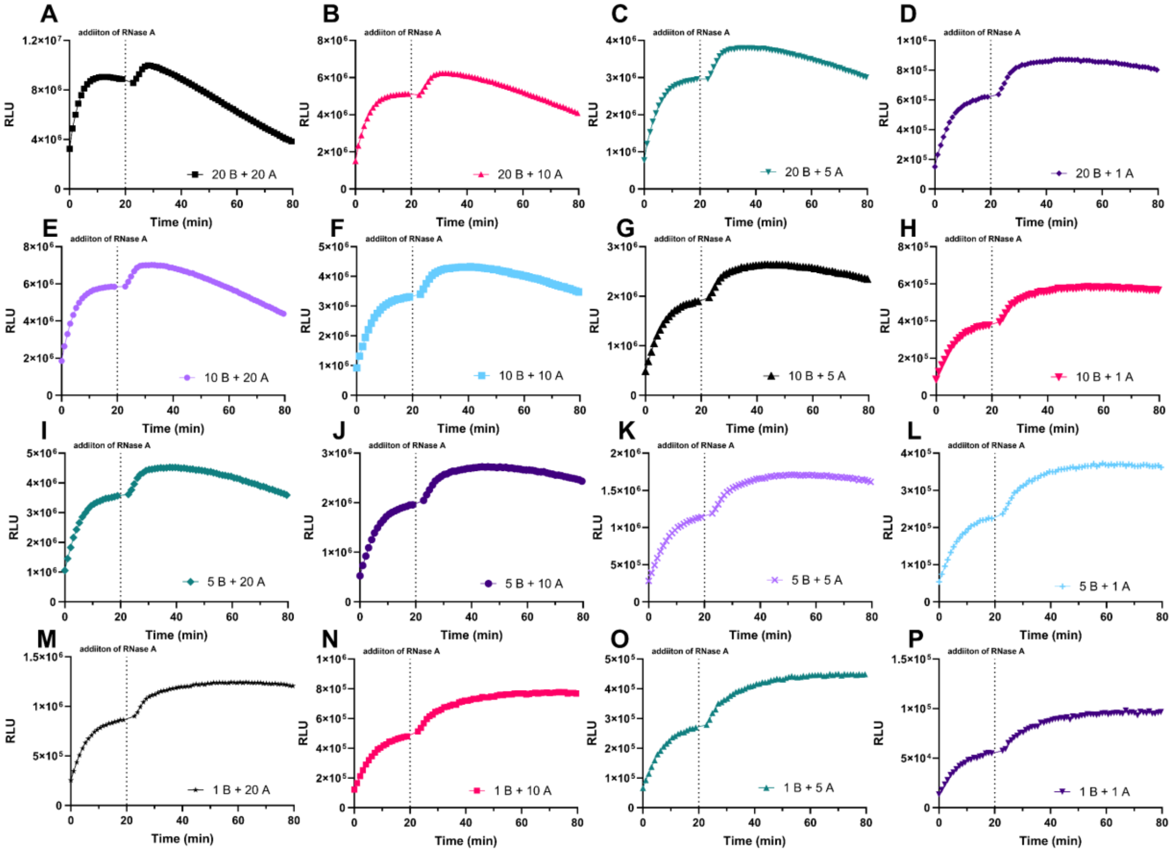

Figure S79: Raw luminescence data for **BC18** treated with RNase A at different concentrations of A protein (**BC18**) and B protein (BH-BWT). (**A**) 20 nM B + 20 nM A. (**B**) 20 nM B + 10 nM A. (**C**) 20 nM B + 5 nM A. (**D**) 20 nM B + 1 nM A. (**E**) 10 nM B + 20 nM A. (**F**) 10 nM B + 10 nM A. (**G**) 10 nM B + 5 nM A. (**H**) 10 nM B + 1 nM A. (**I**) 5 nM B + 20 nM A. (**J**) 5 nM B + 10 nM A. (**K**) 5 nM B + 5 nM A. (**L**) 5 nM B + 1 nM A. (**M**) 1 nM B + 20 nM A. (**N**) 1 nM B + 10 nM A. (**O**) 1 nM B + 5 nM A. (**P**) 1 nM B + 1 nM A.

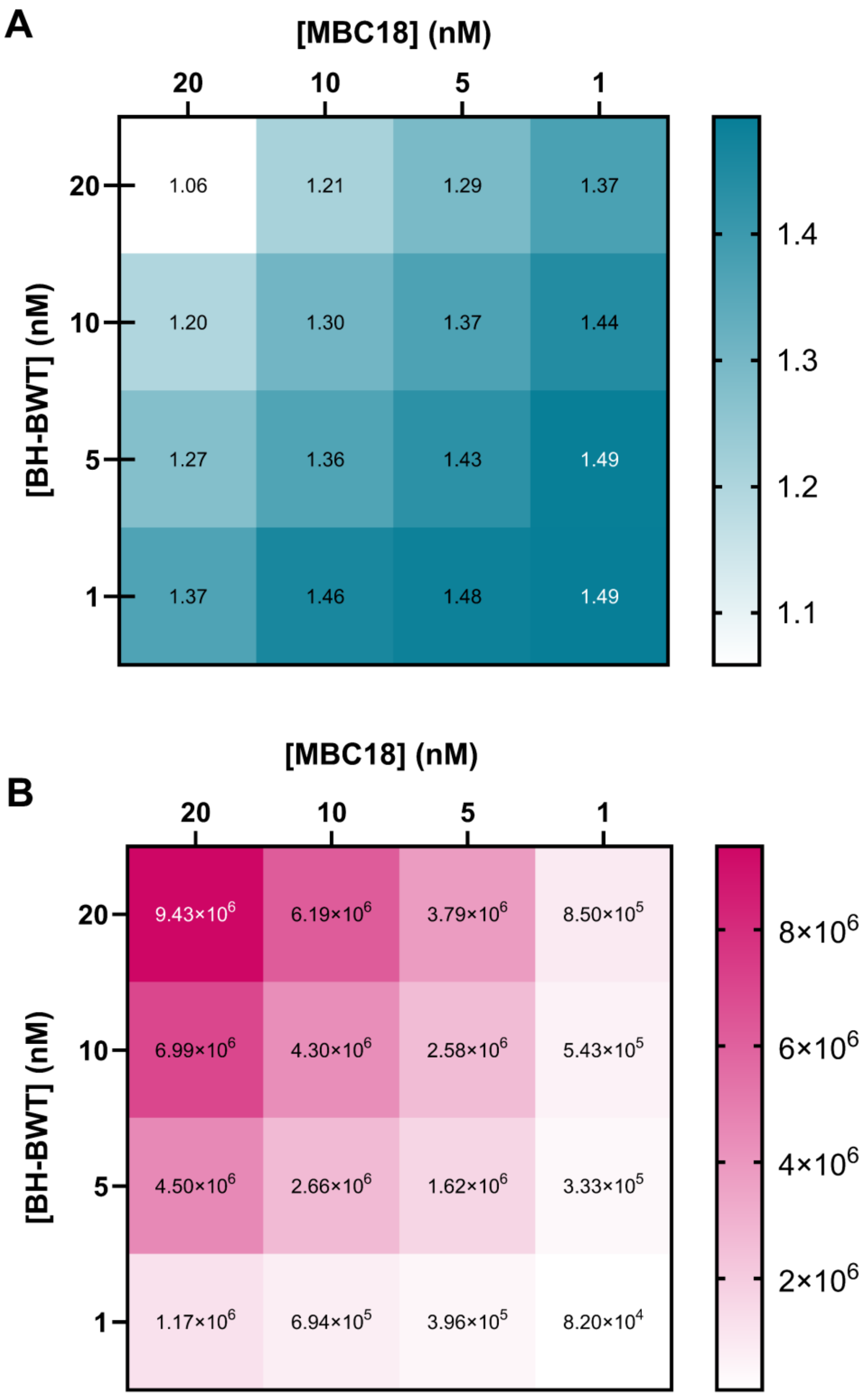

Figure S80: Luminescence data for **BC18** treated with RNase A at different concentrations of A protein (**BC18**) and B protein (BH-BWT). (**A**) Signal-to-baseline (S/B) change after addition of target. Signal measured at baseline and at time = 80 minutes. (**B**) Maximum signal.

6.4 Sensor Limit of Detection (LOD) for target RNA

6.4.1 LOD using 2.5 nM BC10 and 2.5 nM BWT

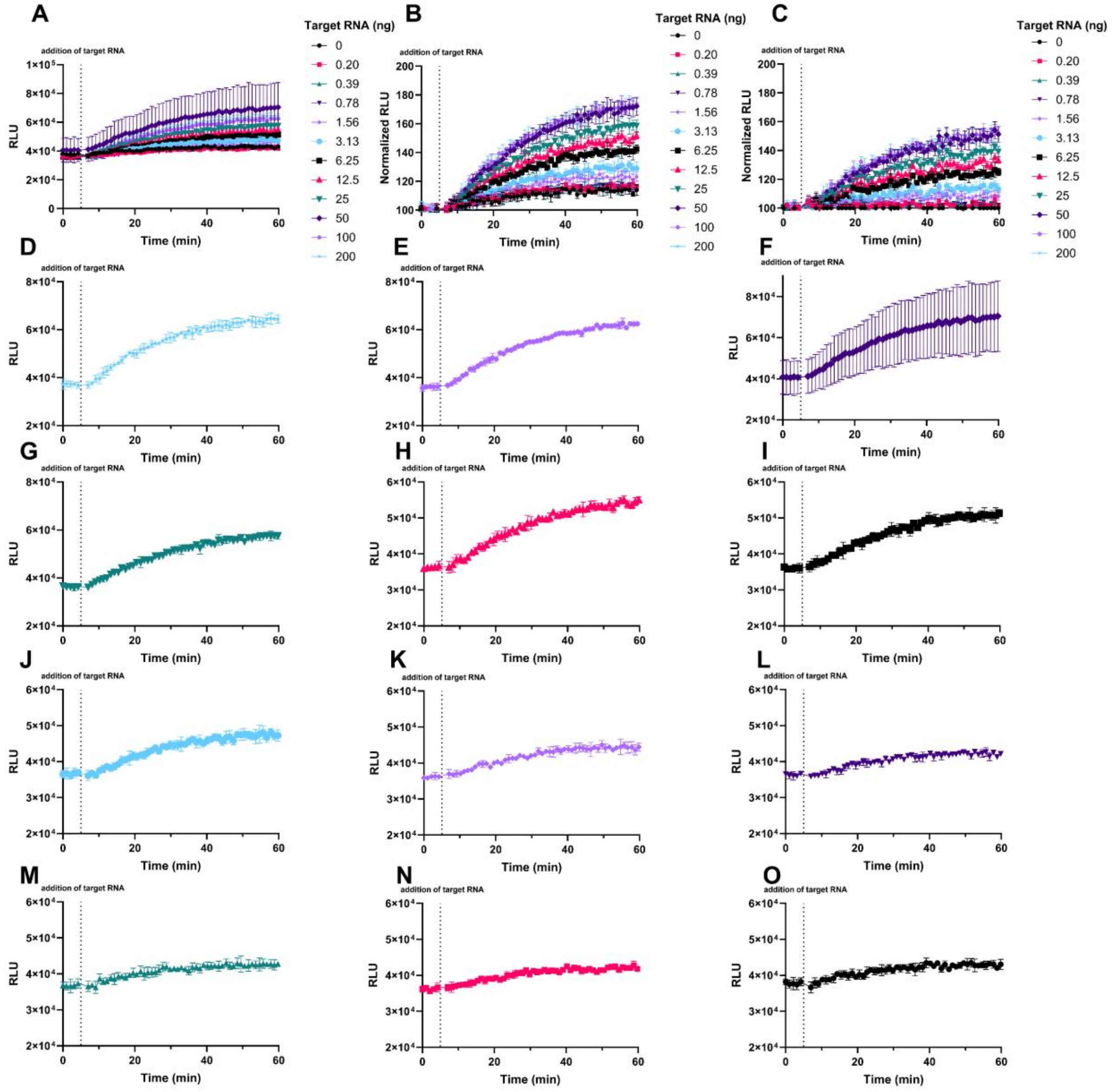

Figure S81: Luminescence data for **BC10** (2.5 nM) and BWT (2.5 nM) treated with Cas13 activated by various amounts of target RNA. (**A**) Combined raw signal. (**B**) Data normalized to baseline. Baseline is defined as the average signal before the addition of target. (**C**) Data normalized to baseline and to the control sample (0 ng of RNA added). (**D**) 200 ng target RNA. (**E**) 100 ng target RNA. (**F**) 50 ng target RNA. (**G**) 25 ng target RNA. (**H**) 12.5 ng target RNA. (**I**) 6.25 ng target RNA. (**J**) 3.13 ng target RNA. (**K**) 1.56 ng target RNA. (**L**) 0.78 ng target RNA. (**M**) 0.39 ng target RNA. (**N**) 0.20 ng target RNA. (**O**) 0 ng target RNA.

6.4.2 LOD using 10 nM BC10 and 5 nM BWT

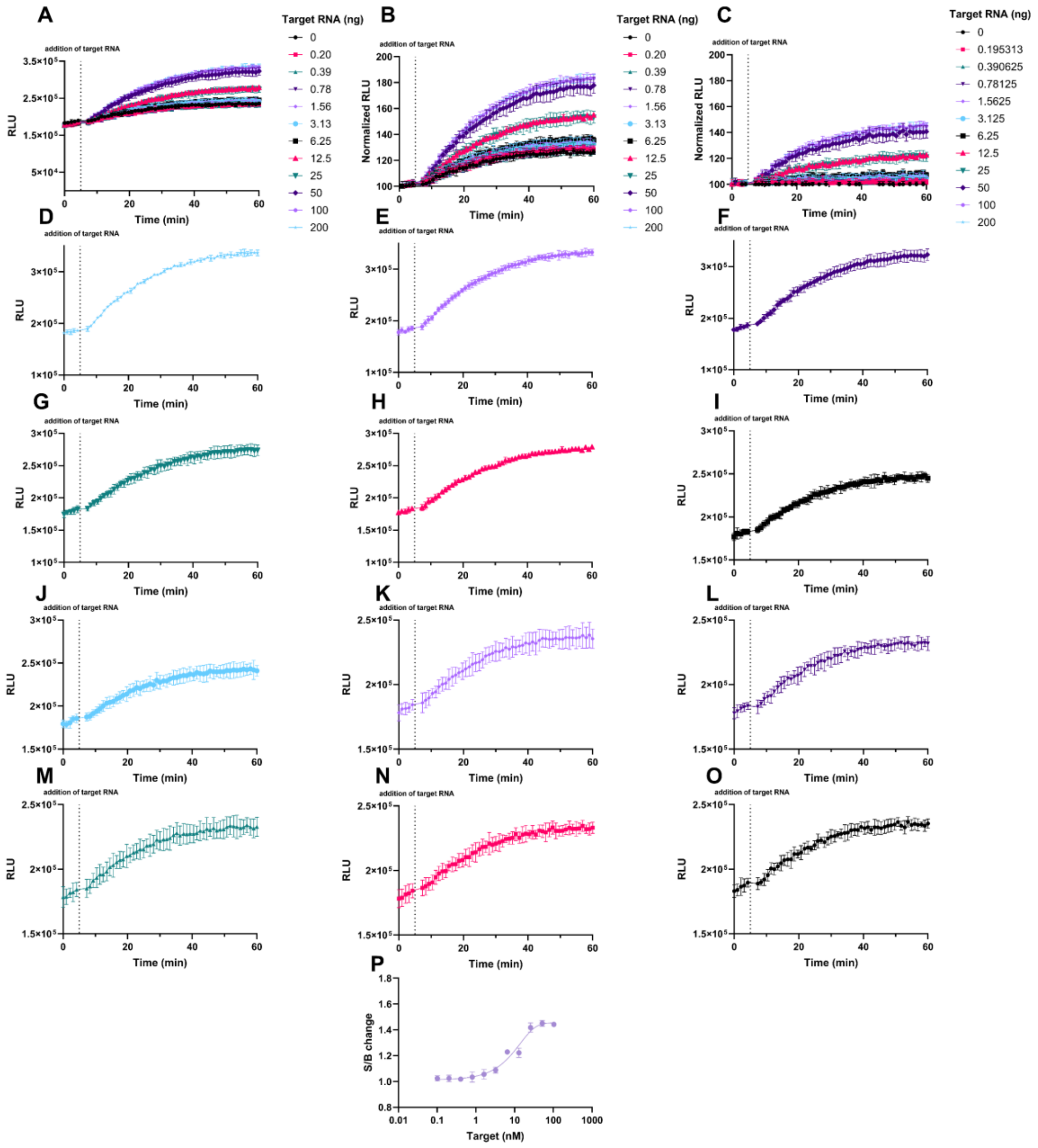

Figure S82: Luminescence data for **BC10** (10 nM) and BWT (5 nM) treated with Cas13 activated by various amounts of target RNA. (**A**) Combined raw signal. (**B**) Data normalized to baseline. Baseline is defined as the average signal before the addition of target. (**C**) Data normalized to baseline and to the control sample (0 ng of RNA added). (**D**) 200 ng target RNA. (**E**) 100 ng target RNA. (**F**) 50 ng target RNA. (**G**) 25 ng target RNA. (**H**) 12.5 ng target RNA. (**I**) 6.25 ng target RNA. (**J**) 3.13 ng target RNA. (**K**) 1.56 ng target RNA. (**L**) 0.78 ng target RNA. (**M**) 0.39 ng target RNA. (**N**) 0.20 ng target RNA. (**O**) 0 ng target RNA. (**P**) Signal-to-baseline (S/B) change versus target RNA concentration. LOD = 2.84 nM.

6.4.3 LOD using 10 nM BC10 and 0.75 nM BWT

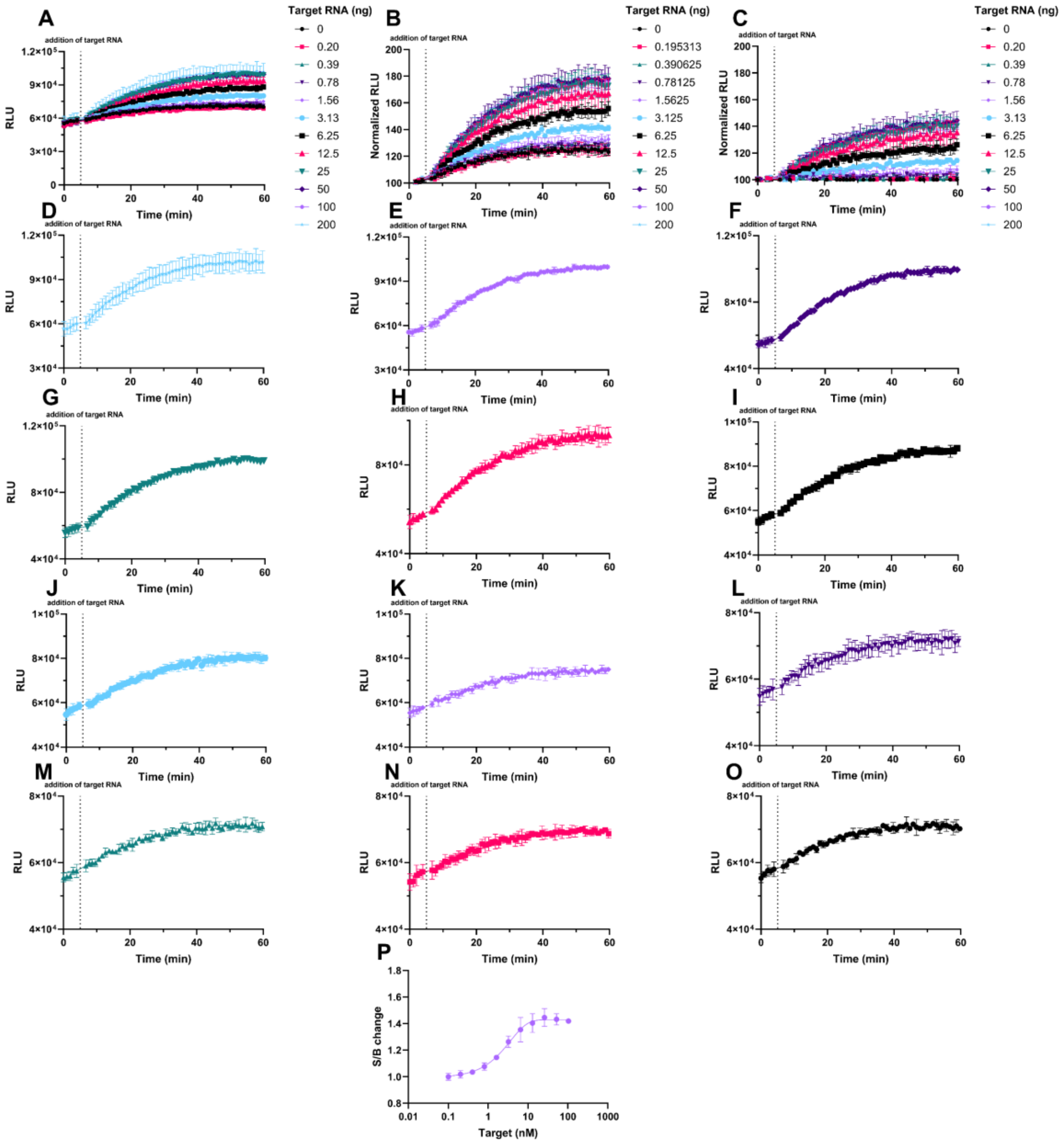

Figure S83: Luminescence data for **BC10** (10 nM) and BWT (0.75 nM) treated with Cas13 activated by various amounts of target RNA. (**A**) Combined raw signal. (**B**) Data normalized to baseline. Baseline is defined as the average signal before the addition of target. (**C**) Data normalized to baseline and to the control sample (0 ng of RNA added). (**D**) 200 ng target RNA. (**E**) 100 ng target RNA. (**F**) 50 ng target RNA. (**G**) 25 ng target RNA. (**H**) 12.5 ng target RNA. (**I**) 6.25 ng target RNA. (**J**) 3.13 ng target RNA. (**K**) 1.56 ng target RNA. (**L**) 0.78 ng target RNA. (**M**) 0.39 ng target RNA. (**N**) 0.20 ng target RNA. (**O**) 0 ng target RNA. (**P**) Signal-to-baseline (S/B) change versus target RNA concentration. LOD = 2.26 nM.

6.5 Viral transcript selectivity

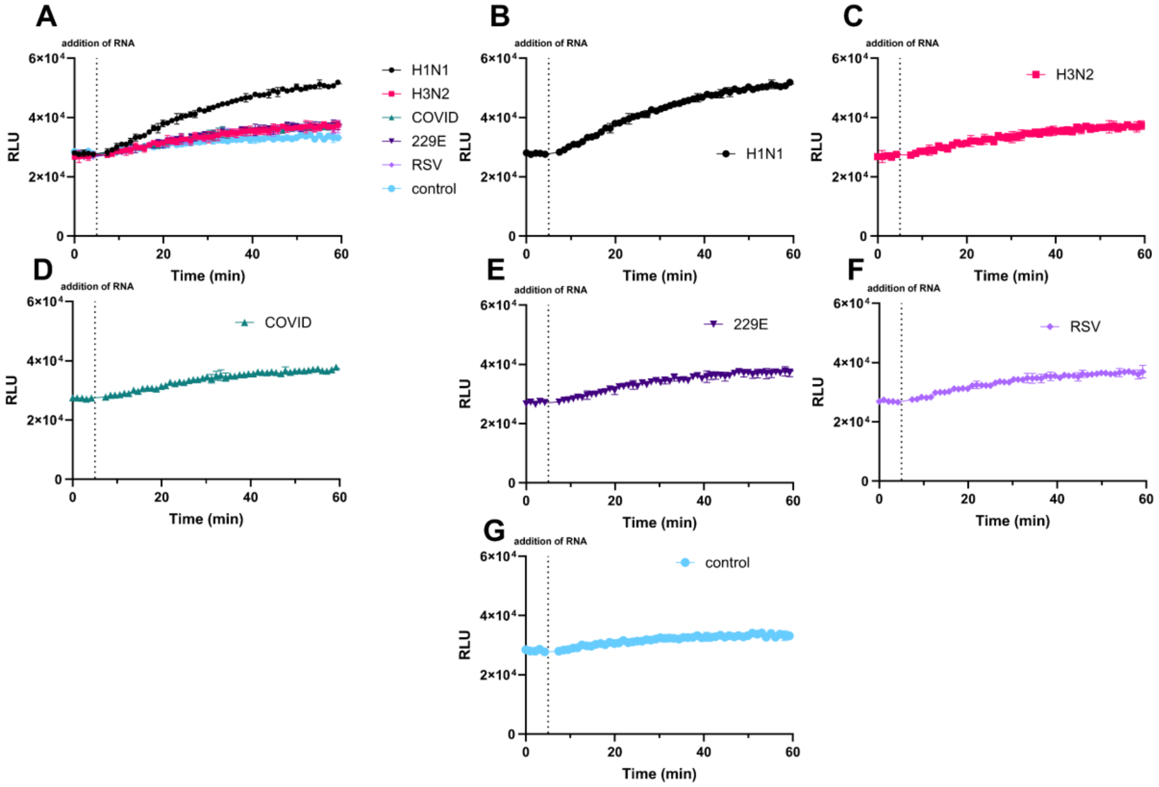

Figure S84: Raw luminescence data for **BC10** treated with Cas13 and various viral transcripts. N = 3 for all sets. (**A**) Combined data sets. (**B**) H1N1 NP transcript. (**C**) H3N2 NP transcript. (**D**) COVID NP transcript. (**E**) hCoV-229E NP transcript. (**F**) RSV NP transcript. (**G**) control (water).

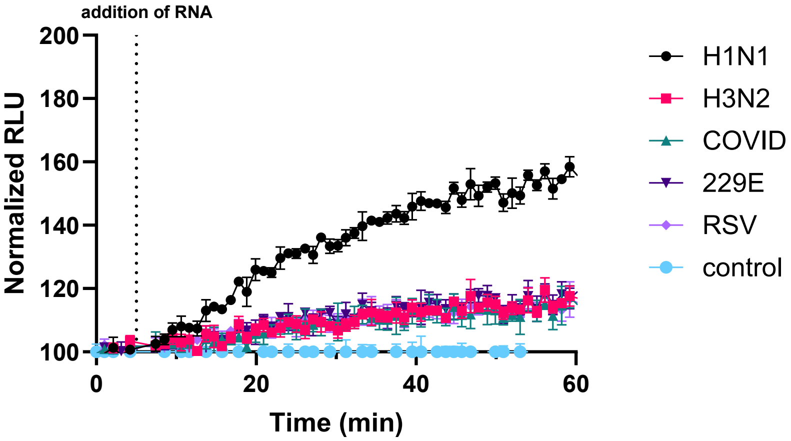

Figure S85: Normalized luminescence data for **BC10** treated with Cas13 and various viral transcripts. N = 3 for all sets. Data normalized to baseline and control signal. Baseline is defined as the average signal before the addition of RNA.

6.6 Sensor efficacy in serum

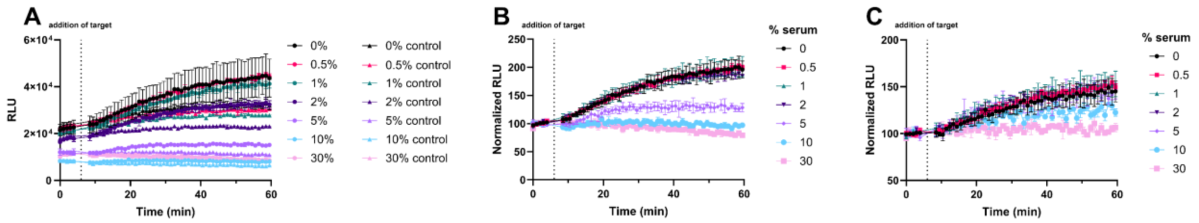

Figure S86: Luminescence data for **BC10** treated with Cas13 in various percentages of serum. N = 3 for all sets. (**A**) Combined raw signal. Control indicates samples without the addition of target RNA. (**B**) Data normalized to baseline. Baseline is defined as the average signal before the addition of target. (**C**) Data normalized to baseline and to respective control signals.

6.7 Long-term luminescence of BC10

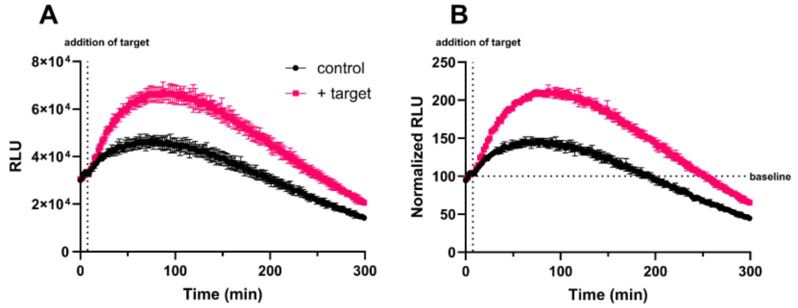

Figure S87: Long term luminescence data for **BC10** and BWT treated with or without Cas13. (**A**) Raw luminescence data. (**B**) Data normalized to baseline. Baseline is defined as the average signal before the addition of target RNA. (**C**) Data normalized to baseline and control signal. "Read" indicates the time where luminescence is evaluated for fold change in all other studies.

6.8 Substrate solvent preference for sensor

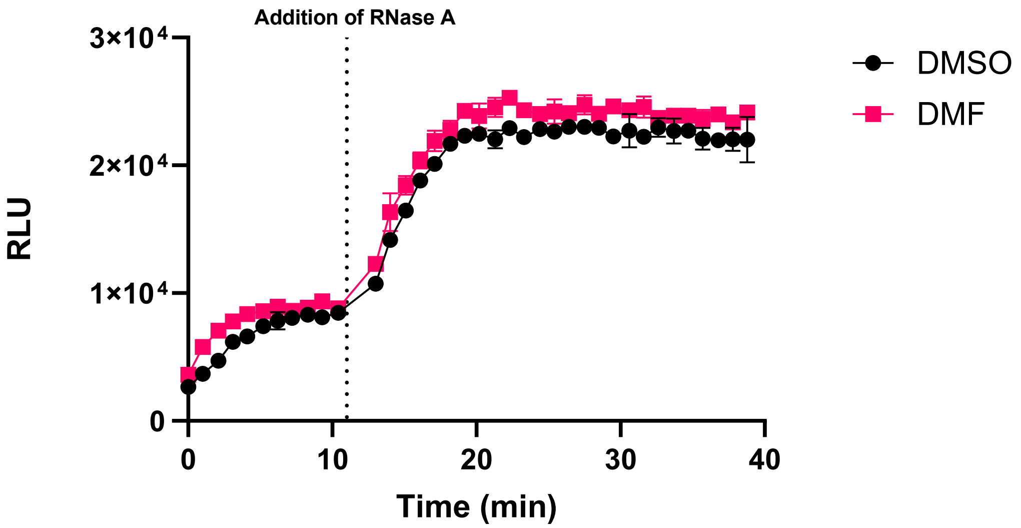

Figure S88: Raw luminescence data for **BC10** and BWT treated with the LuxSit Pro Substrate in 1% DMSO or 1% DMF. N = 2 for both conditions.

6.9 Effect of RNase inhibitor on sensor signal

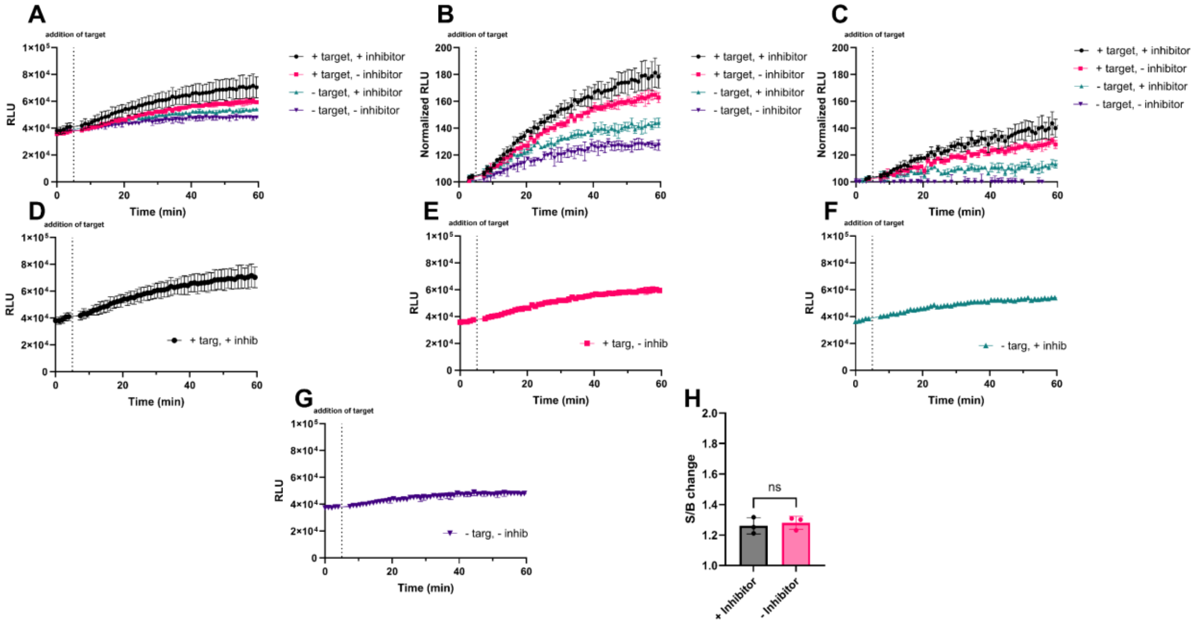

Figure S89: Luminescence data for **BC10** (2.5 nM) and BWT (2.5 nM) treated with Cas13 with or without RNase Inhibitor present. N = 3 for all sets. (**A**) Combined raw signal. (**B**) Data normalized to baseline. Baseline is defined as the average signal before the addition of target. (**C**) Data normalized to baseline to the control sample (-target, -inhibitor). (**D**) +target, +inhibitor. (**E**) +target, -inhibitor. (**F**) -target, +inhibitor. (**G**) -target, -inhibitor. (**H**) S/B change of +target, +inhibitor sample relative to -target, +inhibitor and corresponding change of +target, -inhibitor relative to -target, -inhibitor. There was no statistical difference between the means ($t(4) = 0.5090$, $p = 0.7786$)).

7. Analytical HPLC

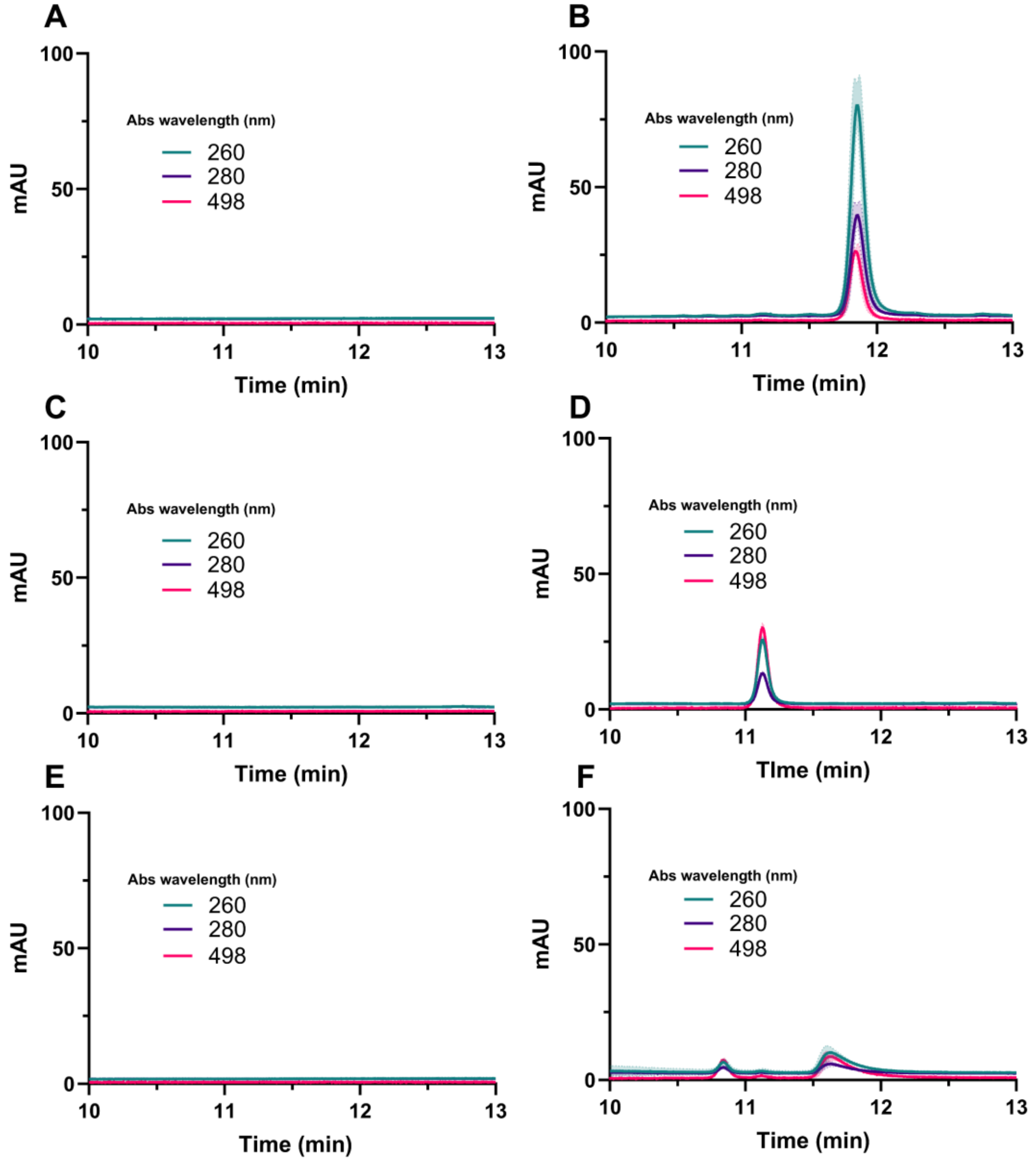

Figure S90: Analytical HPLC chromatogram traces of **conjRNA2** untreated or treated with RNase A or Cas13. N = 3 for panels A, C, and F. N = 2 for panels B, D, and E. Error is shown by shaded regions. (**A**) Water control. (**B**) **ConjRNA2** in water. (**C**) Cas13 without **conjRNA2**. (**D**) Cas13 with **conjRNA2**. (**E**) RNase A without **conjRNA2**. (**F**) RNase A with **conjRNA2**.

Single Mass Analysis
Tolerance = 1500.0 PPM / DBE: min = -1.5, max = 50.0
Element prediction: Off
Number of isotope peaks used for i-FIT = 3

Monoisotopic Mass, Odd and Even Electron Ions
1173 formula(e) evaluated with 1 results within limits (up to 50 closest results for each mass)
Elements Used:
C: 0-54 H: 0-60 N: 0-15 O: 0-24 P: 1-3

12-Nov-2024 09:39:40
GB_MCanzano_MCAS_1 LC neg 3287 (7.419) AM (Cen,4, 80.00, Ht,10000.0,0.00,0.00); Cm (3197:3381)
1: TOF MS ES-
1.87e+004

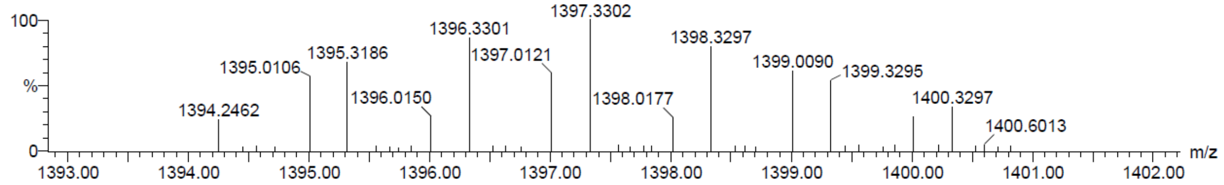

Minimum:				-1.5				
Maximum:		5.0	1500.0	50.0				
Mass	Calc. Mass	mDa	PPM	DBE	i-FIT	Norm	Conf(%)	Formula
1395.3186	1395.3148	3.8	2.7	34.0	195.8	n/a	n/a	C54 H60 N15 O24 P3

Figure S91: 5’ degradation product mass spectrometry results of **conjRNA2** treated with Cas13.

8. NMR

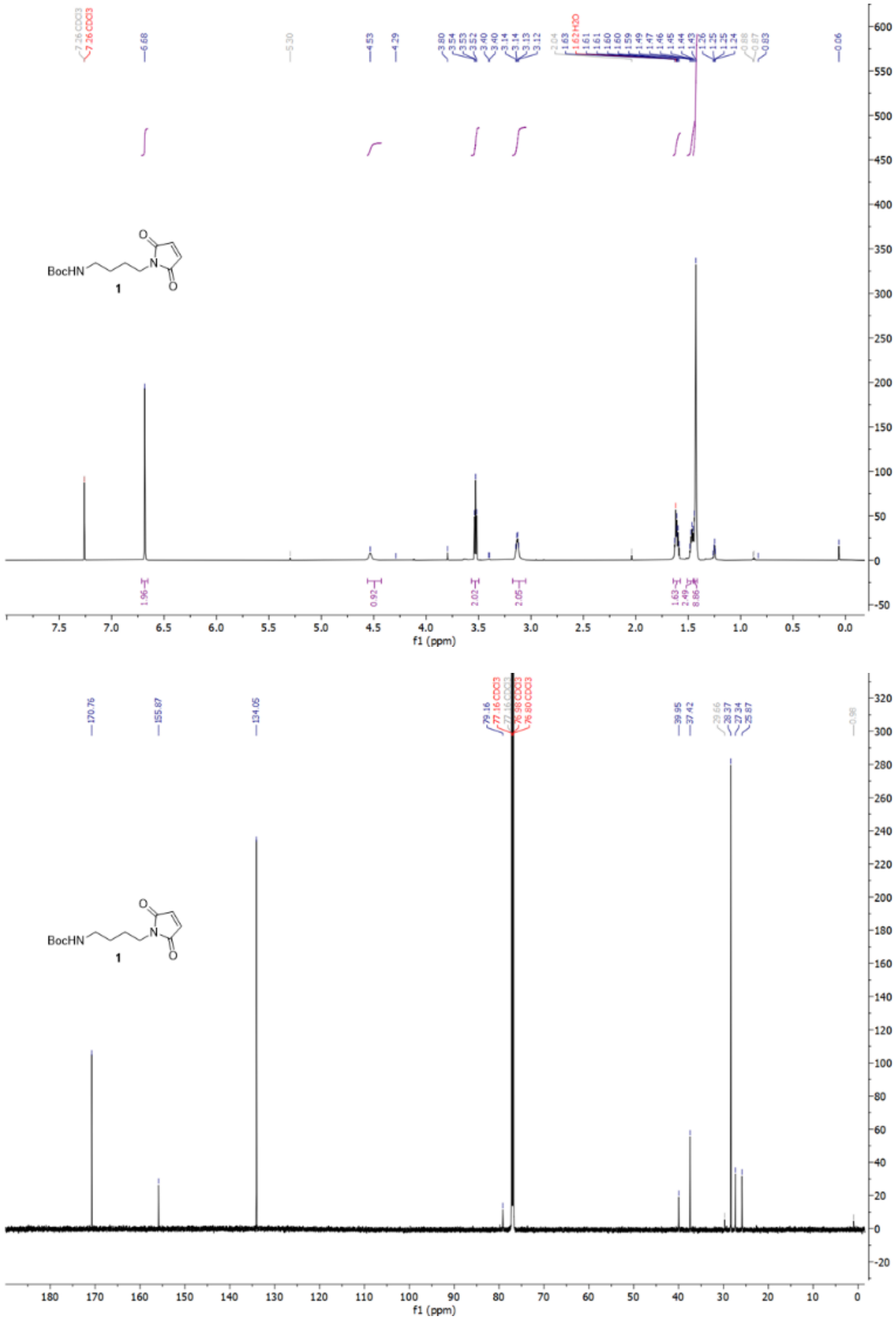

Figure S92: ^{1}H-NMR and ^{13}C-NMR for compound **1**.

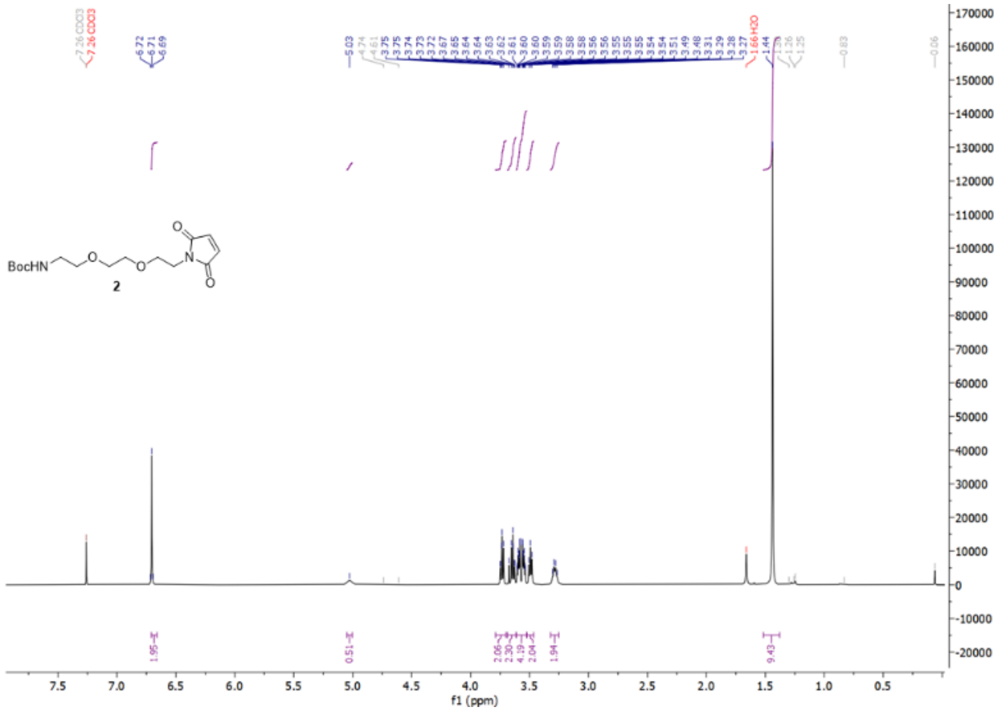

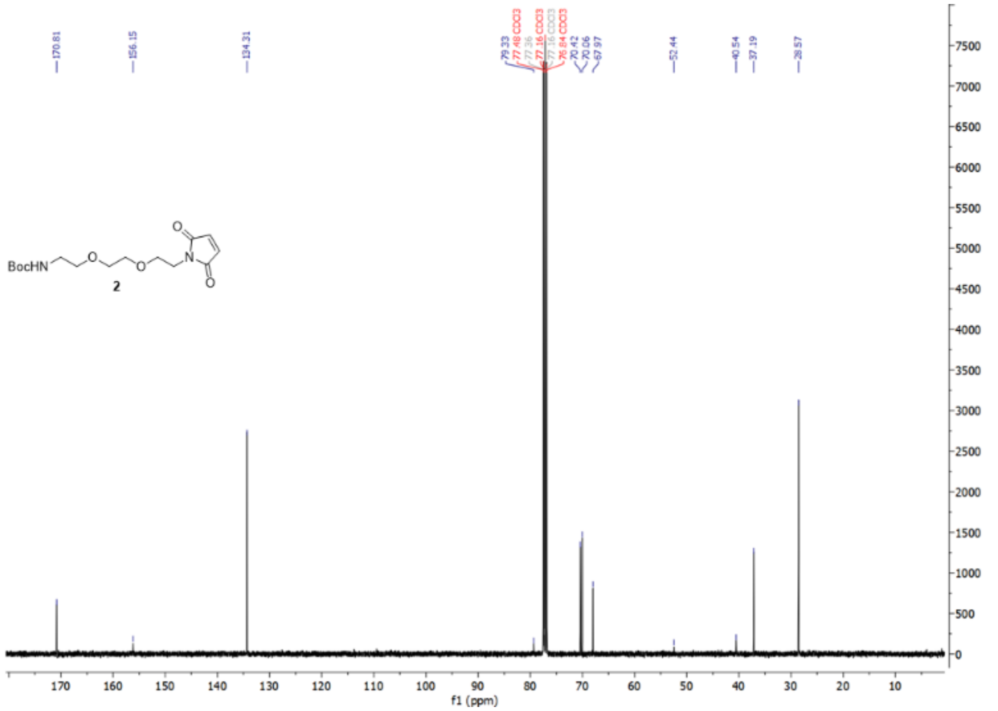

Figure S93: ^{1}H-NMR and ^{13}C-NMR for compound **2**.

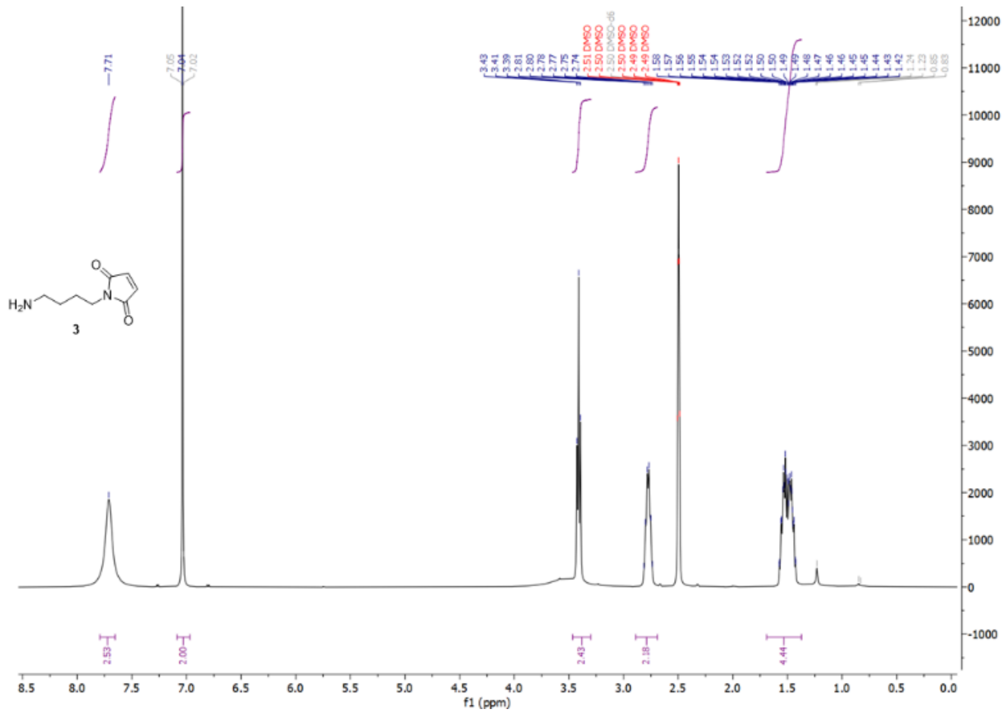

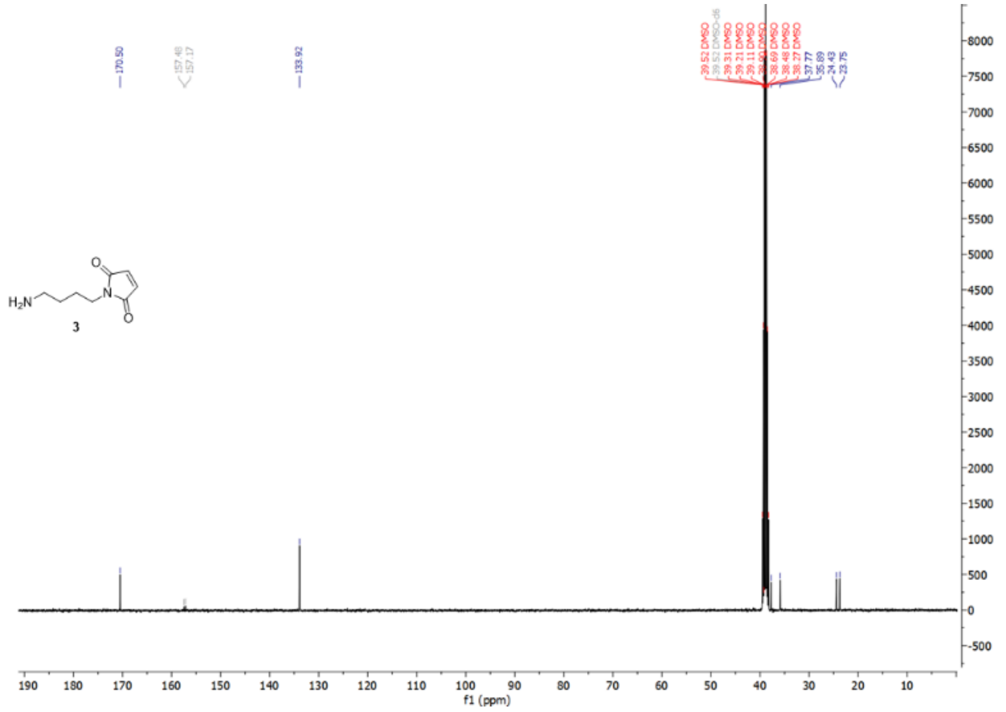

Figure S94: ^{1}H-NMR and ^{13}C-NMR for compound **3**.

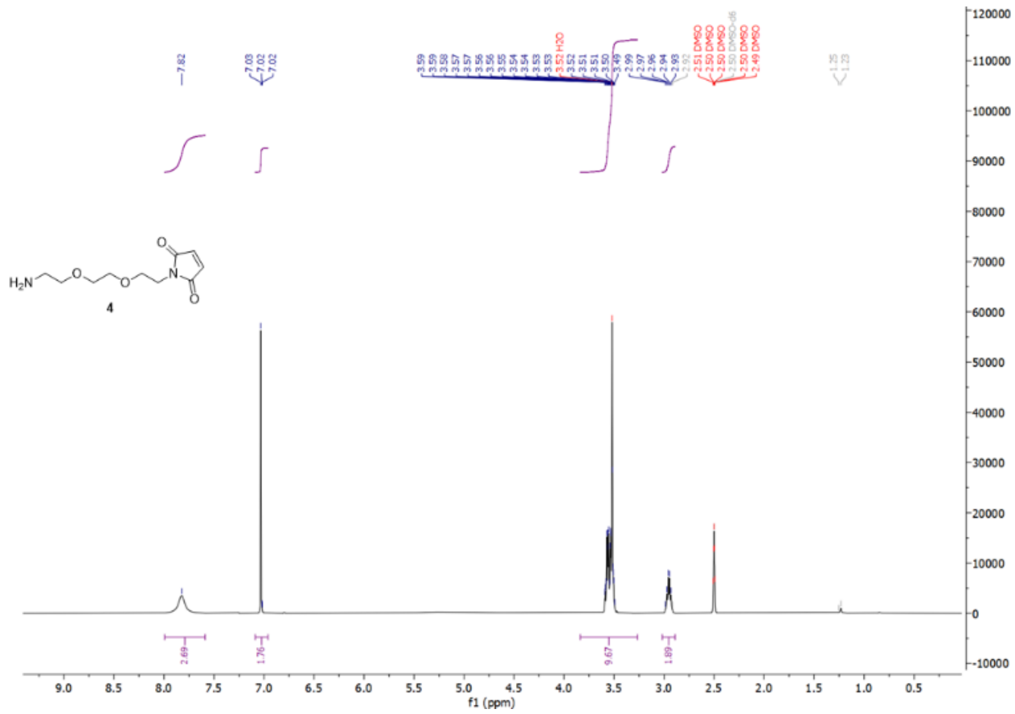

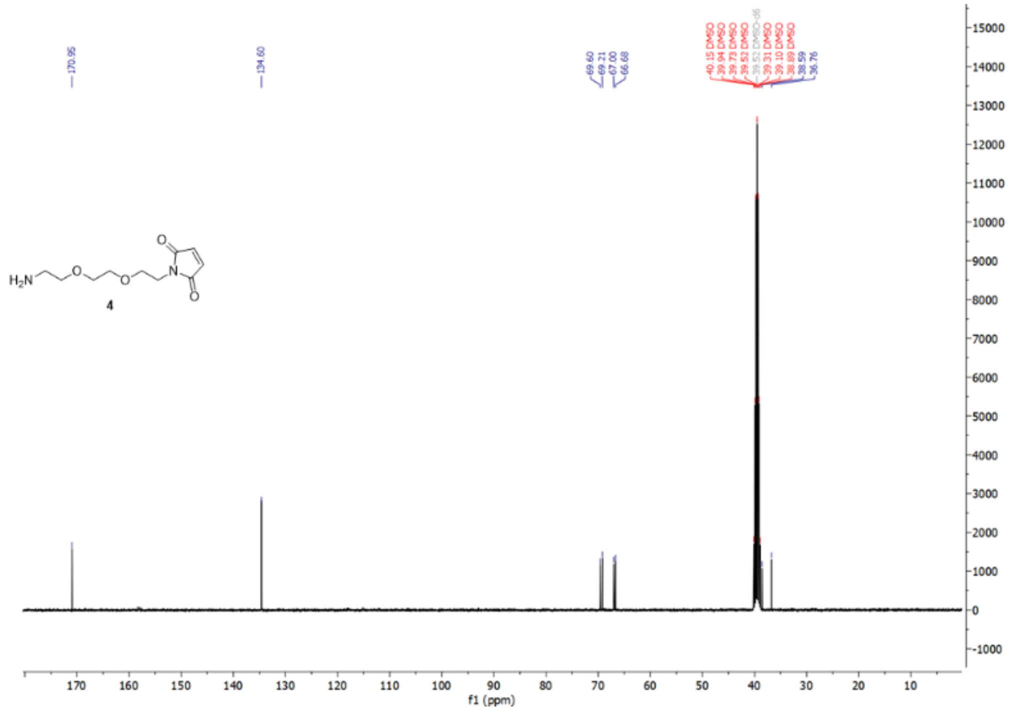

Figure S95: ^{1}H-NMR and ^{13}C-NMR for compound **4**.

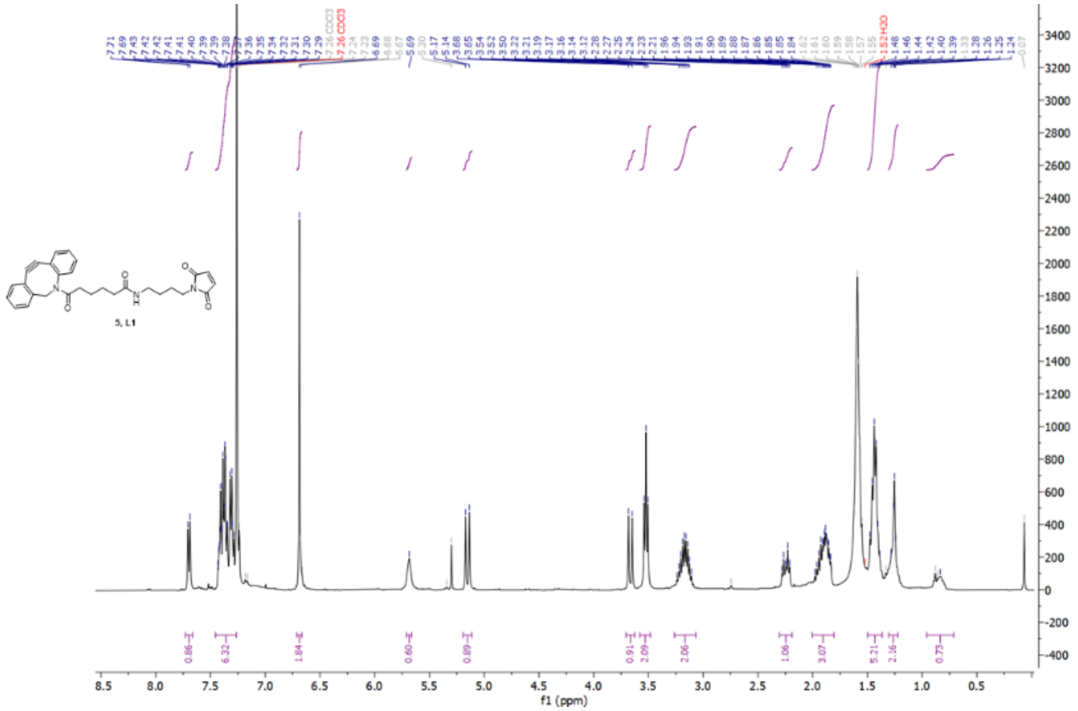

Figure S96: ^{1}H-NMR for compound **5**, linker 1 (**L1**).

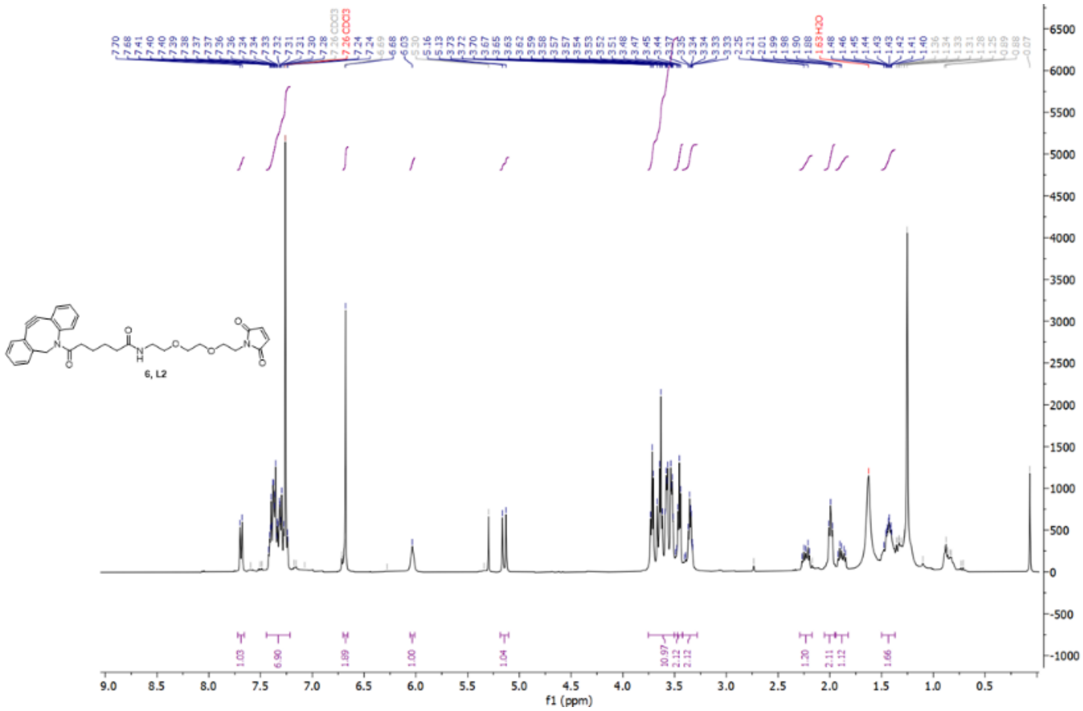

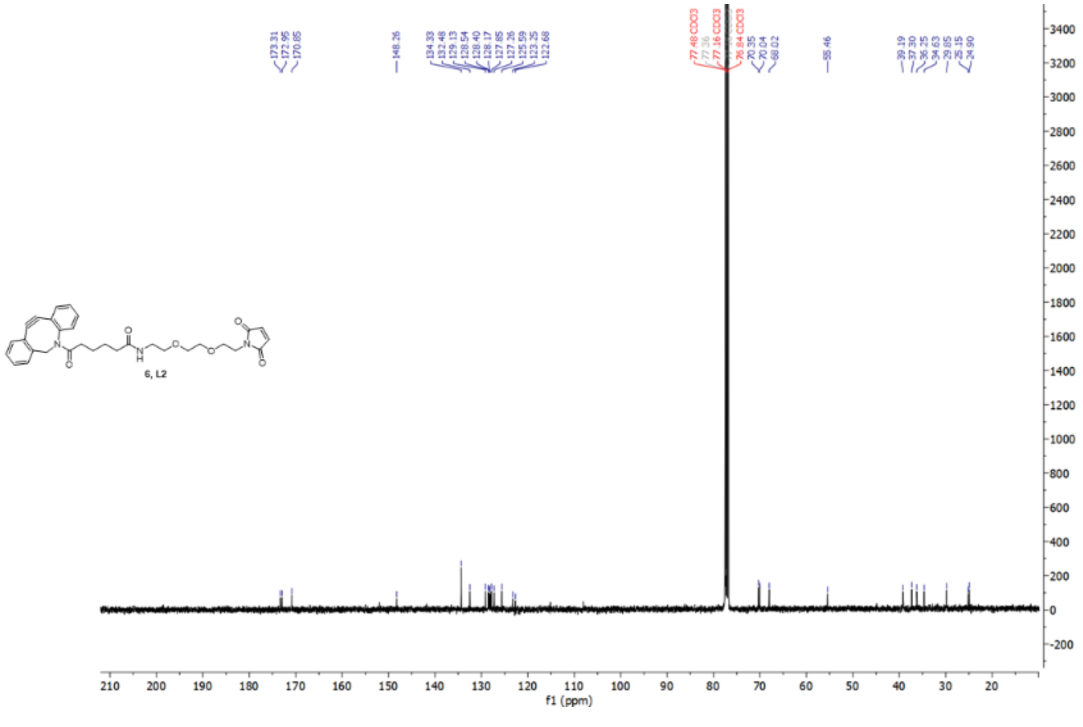

Figure S97: ^{1}H-NMR and ^{13}C-NMR for compound **6**, linker 2 (**L2**).

9. References

(1) Marty, M. T.; Baldwin, A. J.; Marklund, E. G.; Hochberg, G. K. A.; Benesch, J. L. P.; Robinson, C. V. Bayesian Deconvolution of Mass and Ion Mobility Spectra: From Binary Interactions to Polydisperse Ensembles. *Anal. Chem.* **2015**, *87* (8), 4370–4376. https://doi.org/10.1021/acs.analchem.5b00140.

(2) Conrad, T.; Plumbom, I.; Alcobendas, M.; Vidal, R.; Sauer, S. Maximizing Transcription of Nucleic Acids with Efficient T7 Promoters. *Commun. Biol.* **2020**, *3* (1), 1–8. https://doi.org/10.1038/s42003-020-01167-x.

(3) Sayers, E. W.; Beck, J.; Bolton, E. E.; Brister, J. R.; Chan, J.; Comeau, D. C.; Connor, R.; DiCuccio, M.; Farrell, C. M.; Feldgarden, M.; Fine, A. M.; Funk, K.; Hatcher, E.; Hoeppner, M.; Kane, M.; Kannan, S.; Katz, K. S.; Kelly, C.; Klimke, W.; Kim, S.; Kimchi, A.; Landrum, M.; Lathrop, S.; Lu, Z.; Malheiro, A.; Marchler-Bauer, A.; Murphy, T. D.; Phan, L.; Prasad, A. B.; Pujar, S.; Sawyer, A.; Schmieder, E.; Schneider, V. A.; Schoch, C. L.; Sharma, S.; Thibaud-Nissen, F.; Trawick, B. W.; Venkatapathi, T.; Wang, J.; Pruitt, K. D.; Sherry, S. T. Database Resources of the National Center for Biotechnology Information. *Nucleic Acids Res.* **2024**, *52* (D1), D33–D43. https://doi.org/10.1093/nar/gkad1044.

(4) Guo, X.; Rahman, J. A.; Wessels, H.-H.; Méndez-Mancilla, A.; Haro, D.; Chen, X.; Sanjana, N. E. Transcriptome-Wide Cas13 Guide RNA Design for Model Organisms and Viral RNA Pathogens. *Cell Genomics* **2021**, *1* (1), 100001. https://doi.org/10.1016/j.xgen.2021.100001.

(5) Wessels, H.-H.; Méndez-Mancilla, A.; Guo, X.; Legut, M.; Daniloski, Z.; Sanjana, N. E. Massively Parallel Cas13 Screens Reveal Principles for Guide RNA Design. *Nat. Biotechnol.* **2020**, *38* (6), 722–727. https://doi.org/10.1038/s41587-020-0456-9.

(6) Labun, K.; Montague, T. G.; Krause, M.; Torres Cleuren, Y. N.; Tjeldnes, H.; Valen, E. CHOPCHOP v3: Expanding the CRISPR Web Toolbox beyond Genome Editing. *Nucleic Acids Res.* **2019**, *47* (W1), W171–W174. https://doi.org/10.1093/nar/gkz365.

(7) Gootenberg, J. S.; Abudayyeh, O. O.; Lee, J. W.; Essletzbichler, P.; Dy, A. J.; Joung, J.; Verdine, V.; Donghia, N.; Daringer, N. M.; Freije, C. A.; Myhrvold, C.; Bhattacharyya, R. P.; Livny, J.; Regev, A.; Koonin, E. V.; Hung, D. T.; Sabeti, P. C.; Collins, J. J.; Zhang, F. Nucleic Acid Detection with CRISPR-Cas13a/C2c2. *Science* **2017**, *356* (6336), 438–442. https://doi.org/10.1126/science.aam9321.
